# Extracellular Antibody Drug Conjugates Exploiting the Proximity of Two Proteins

**DOI:** 10.1038/mt.2016.119

**Published:** 2016-07-19

**Authors:** David J Marshall, Scott S Harried, John L Murphy, Chad A Hall, Mohammed S Shekhani, Christophe Pain, Conner A Lyons, Antonella Chillemi, Fabio Malavasi, Homer L Pearce, Jon S Thorson, James R Prudent

**Affiliations:** 1 Centrose, Madison, Wisconsin, USA; 2 Laboratory of Immunogenetics, Department of Medical Sciences, University of Torino, Torino, Italy; 3 Center for Pharmaceutical Research and Innovation, University of Kentucky College of Pharmacy, Lexington, Kentucky, USA

## Abstract

The human Na^+^/K^+^-ATPase (NKA) is a plasma membrane ion pump that uses ATP to help maintain the resting potential of all human cells. Inhibition of the NKA leads to cell swelling and death. The results of this investigation show that on cancer cells, the NKA either comes in close proximity to, associate with or complexes to important cancer-related proteins, and thus can be targeted with a new type of precision therapy called the extracellular drug conjugate or EDC. The EDCs reported here exhibit EC_50_ values in the low to mid-picomolar range, and signal to noise ratios > 1,000:1, both of which are dependent on the cell surface expression of the NKA and corresponding cancer-related target. We demonstrate that a potent small molecule inhibitor of the NKA can be covalently attached to antibodies targeting CD20, CD38, CD56, CD147, or dysadherin, to create a series of selective and powerful EDCs that kill cancer cells extracellularly by a mechanism resembling necrosis. This is therefore a framework for the development of a new type of precision therapy wherein exquisite selectivity is achieved for targeting extracellular disease-related proteins.

## Introduction

In mammals, the Na^+^/K^+^-ATPase is a prominent example of an active cell surface ATPase ion pump that is responsible for maintaining transmembrane concentration gradients of both sodium and potassium.^1,2,3,4,5,6,7^ This ion pump consists of three membrane-spanning subunits (α, β, and γ) each comprising multiple isoforms.^8,9^ Of the three subunits, only the α-subunit actively pumps ions in an ATP-dependent manner, resides predominantly inside the cell, and is inhibited by cardiac glycoside (CG) binding.^10^ Though it is not known why CGs evolved, it is known that they bind a deep extracellular pocket within the α-subunit with high affinity and specificity.^11,12,13,14^ Extensive clinical studies have led to the approval and wide clinical use of certain CGs for the treatment of heart failure.^15^

Beyond ion trafficking, additional biological activities attributed to CGs have been reviewed elsewhere.^16,17,18,19^ With regard to their antiproliferative activities, CGs have intrigued yet puzzled scientists since they were first discovered.^20,21,22,23^ Reasons for the intrigue include their nanomolar effective concentrations (EC_50_) and their abilities to act on cancers that are metastatic, hypoxic, cytoprotective, and drug resistant.^24^ Yet after years of research and multiple clinical trials, no statistically significant clinical benefit in the treatment of cancer has been demonstrated.^25^ A major reason for treatment failure is the narrow therapeutic index (TI) of the CG class of drugs.

We set out to determine whether the negative effects of CGs elicited on normal tissues could be minimized by the precise targeting of CGs to NKA α-subunit specific protein-protein interactions. One protein known to interact with the α-subunit and be overexpressed on the cells of many metastatic cancers is dysadherin (DYS) a gamma subunit of the NKA (also known as a FXYD family protein).^26^ Hence, we constructed a new type of antibody drug conjugate (ADC) that targets extracellular protein-protein interactions and termed these, extracellular drug conjugates or EDCs. After demonstrating increases in potency and specificity with the initial EDC-DYS conjugate, several other EDCs were constructed with other antibodies specific to important cancer related proteins (CD20, CD38, CD147, CD56). Here, we present a thorough discussion and characterization of these EDCs and their therapeutic potential.

## Results

### EDC construction (CG, mAb, and linker) and the effect of linker length

**Figure 1:**
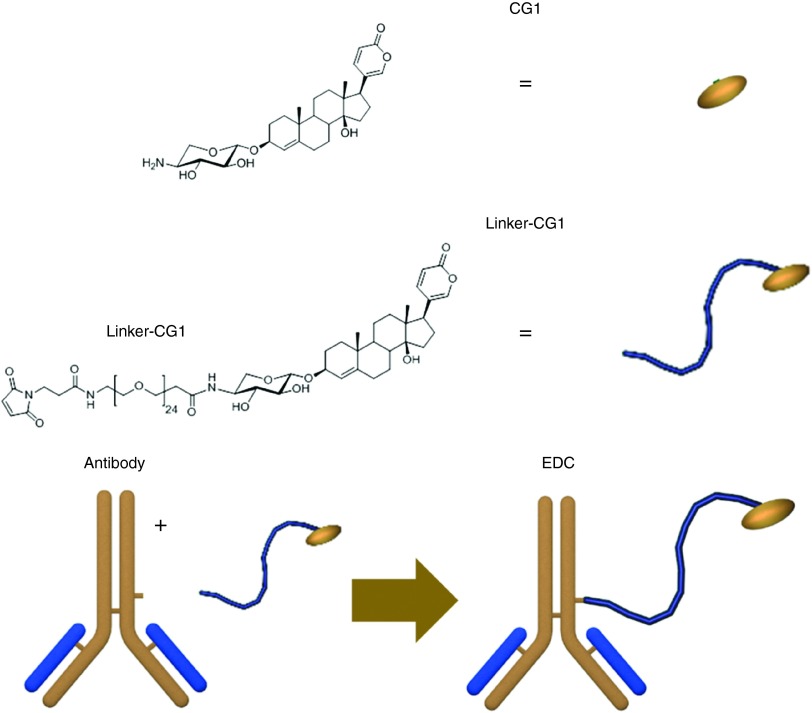
**EDC parts and construction schematic**. Basic components of the EDCs described in this study are: CG1 (the drug), the Linker-CG1, mAb and the EDC. **CG1** was first synthesized and covalently attached to a bifunctional linker via NHS coupling to make **Linker-CG1**. After antibody hinge region disulfides are reduced, Linker-CG1 is added to form the **EDC** which was then filtered to remove unbound Linker-CG1. All steps and characterization of Linker-CG1 and its intermediates are described in detail in **Supplementary Data**.

Previous data regarding CGs highlighted the importance of a six-member α-pyrone ring, a 14β-OH group, and a C-4 double bond.^27^ For conjugation purposes, previous data also suggested that amines within the sugar moiety maintain pharmacological properties of CGs.^28^ Applying this knowledge, we produced a library of differentially amino-glycosylated CGs and evaluated their activities. One of the most active CGs in the library, scillarenin β-L-aminoxyloside was designated CG1 and used to construct the extracellular antibody drug conjugates (EDCs) discussed throughout this study (**Figure 1**).

The nine monoclonal antibodies (mAbs) discussed in this study are specific to targets that are: (i) commonly known to associate with the NKA and considered a metastatic cancer marker (dysadherin)^26^; (ii) previously suggested to associate with the NKA and cancer related (CD56, CD147)^29,30^; (iii) suggested by the results shown in this study to associate with the NKA and current cancer antibody drug targets (CD20, CD38)^31,32^; (iv) or suggested by the results in this study not to associate with the NKA (*i.e*., fibroblast-activating protein (FAP)^33^ and HER2/neu (HER2)); or (v) not expressed on any cell surface (*i.e.*, fluorescein or peptide (CONTROLS)).

**Figure 2:**
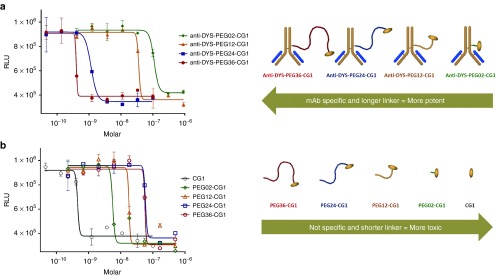
**Effect of linker length on EDC activity**. (**a**) Dose response curves of A549 cells treated with EDCs constructed with the anti-dysadherin (DYS) antibody linked to CG1 via four different polyethylene glycol (PEG) linkers with 2 (green diamond), 12 (gold triangle), 24 (blue square), or 36 (red circle) repeating units spanning different lengths (27, 56, 105, 144 angstroms respectively as measured by bond distances). The figure to the right of the graph represent the 4 EDCs in order of activity from higher activity to lower activity (left to right). (**b**) Dose response curves of A549 cells treated with CG1 (black open circle) and 4 the different Linker-CG1s (polyethylene glycol (PEG) polymers with 2 (open green diamond), 12 (open gold triangle), 24 (open blue square), or 36 (open red circle) repeating units spanning different lengths (27, 56, 105, 144 angstroms respectively as measured by bond distances). The figure to the right of the graph represent the four Linker-CG1s in order of activity from higher activity to lower activity (left to right). Based on linker length, notice how the activities of the EDCs are reversed when compared to the Linker-CG1s.

To select an EDC linker, we conjectured that flexibility and length would be critical. We therefore used four distinct bifunctional polyethylene glycol linkers (PEGs) of varying lengths (27 to 144 Å as measured by bond distances) to conjugate anti-dysadherin (DYS) and anti-CD147 (CD147) mAbs to CG1. The activities of these eight EDCs along with the four linker-CG1 molecules alone (devoid mAb) were analyzed *in vitro* for growth inhibition activity (**Figure 2** (DYS) and **Supplementary Figures S1A** (CD147) and **S1B** (Linker-CG1s alone) and **Supplementary Tables S1A** and **S1B**). In general, EDCs with longer linkers exhibited greater activity (**Figure 2a**) while linker-CG1 molecules with no targeting antibody exhibited the opposite effect (**Figure 2b**). Large inflection points of activity were observed for the DYS conjugates having linker lengths between 63 and 105 Å and CD147 conjugates having linker lengths between 27 and 63 Å. When linker-CG1s were attached to antibodies that did not recognize any cell surface protein (EDC-CONTROL), their activities were poor and resembled linker-CG1s alone or EDCs with short linkers (**Supplementary** **Figure S1c** and **Supplementary Table S2**). These results clearly indicated that the targeting antibody and the long linkers were both critical to obtaining maximal EDC potency and specificity and thus, throughout the rest of the study, we constructed and tested EDCs using CG1 covalently attached to targeting antibodies via linkers spanning at least 105 Å.

### The effects of EDC on cell growth

**Figure 3:**
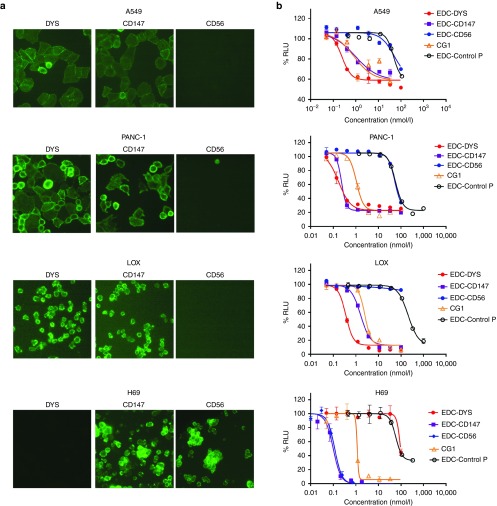
**Antibody target expression is required for EDC activity**. (**a**) Immunofluorescence staining images of live A549, PANC-1, LOX, and H69 cells stained with antibodies specific to; dysadherin (DYS), CD147, and CD56. (**b**) Dose response curves for cell lines in **a** treated with EDC-DYS, EDC-CD147, EDC-CD56, EDC-Control P, and free CG1.

EDCs were constructed with mAbs specific to the nine distinct targets, and their effects on a diverse array of cell types were evaluated (**Supplementary Table S2**). First, the presence (or absence) of the antibody target on the surface of live cells was assessed via antibody fluorescent staining of unfixed, live cells (**Figure 3** and **Supplementary Table S2**). Next, cell sensitivity to the EDCs was assessed at concentrations ranging from 1 to 200,000 pmol/l by monitoring cell viability (**Figure 3** and **Supplementary Table S2**). In instances where the antibody target was expected or experimentally hypothesized to interact with the NKA, the resulting data indicated strong EDC activity and specificity, exemplified by the picomolar EC_50_ values observed for EDC-DYS, EDC-CD147, and EDC-CD56. In these examples, cancer cells expressing the monoclonal antibody targets were particularly sensitive to the corresponding EDC (EC_50_ values between 80 and 440 pmol/l), while cancer cells not expressing the targets were relatively resistant (EC_50_ values > 50,000 pmol/l). Furthermore, control conjugates (*e.g.*, EDC-CONTROL 1 and 2) constructed with antibodies that do not stain cells exhibited background levels of activity (EC_50_ > 100,000 pmol/l). As predicted, all human cell types examined were sensitive to the free unconjugated form of CG1 (EC_50_ between 700 and 3,100 pmol/l). These results suggest that unlike CG1, in order to achieve subnanomolar EDC activity, cell surface antibody target expression was indispensable. It should be noted that when CG1 was mixed but not covalently attached to a targeting monoclonal antibody and compared to CG1 alone, no significant change in cell cytotoxicity was ever observed.

Two of the EDCs synthesized (EDC-CD20 and EDC-CD38) against targets not previously known to interact with the NKA also displayed potent activities (EC_50_ between 20 and 100 pmol/l). There was no activity above background observed for any of the cancer cells treated with EDC-FAP or EDC-HER2 (EC_50_ >100,000 pmol/l), even though the presence of both targets was confirmed via live cell antibody staining and CG1 sensitivity. Similar negative results were obtained with EDC using antibodies to CD19, CD22, CD29, CD47, CD71, CD92, CD166, CD221, CD243, and CD326 (data not shown). With the exception of the Rituximab (anti-CD20 antibody), none of the naked antibodies tested in this study yielded detectable direct cytotoxic effects. To determine if the conjugation of CG1 to rituximab was simply an additive effect, we compared EDC-CD20 to a mixture of CG1 and Rituximab on cells either sensitive to all three of just CG1 and EDC-CD20. The data shows that rituximab does not add to the cytotoxic effects of CG1 even on cells sensitive to Rituximab alone (**Supplementary Figure S1D**).

**Figure 4:**
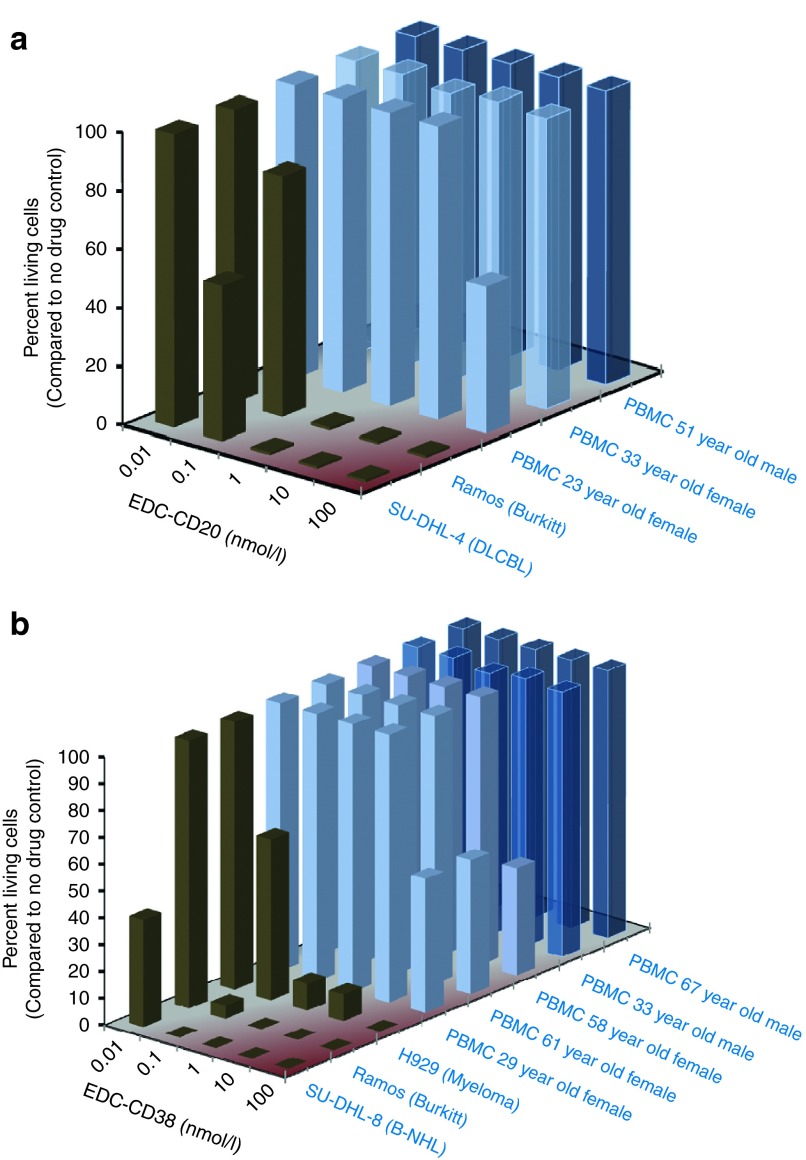
**Effects of EDC on cell viability**. (**a**) EDC-CD20. (**b**) EDC-CD38. Concentration sensitivity of cancer and normal cells to EDC were compared by determining the percent living cells after exposure using two different experimental protocols. Cancer cells were treated with EDC-CD20 for 72 hours and viability determined by CellTiter-Glo analysis. peripheral blood mononuclear cells were treated with EDC-CD20 for 20 hours, stained by annexin V and propidium iodide and analyzed by flow cytometry.

To test whether the active EDCs were toxic to normal human cells, peripheral blood mononuclear cells (PBMCs) collected from healthy individuals of varying age and sex were treated with EDC-DYS, EDC-CD147, EDC-CD20, EDC-CD38, or EDC-CONTROL. The effects were monitored by assaying apoptosis (**Figure 4** and **Supplementary Table S2**). In contrast to the results obtained with cancer cells, EDCs were considerably less cytotoxic to PBMCs (EC_50_ ≥ 100,000 pmol/l). Knowing that endothelial and epithelial cells express both dysadherin and CD147 on their surface, EDC-DYS, EDC-CD147, and EDC-CONTROL were added to cultured primary kidney endothelial cells and primary arterial epithelial cells. Again the active EDCs were considerably less toxic to the normal cells (EC_50_ > 200,000 pmol/l) than they were to cultured tumor cells (EC_50_ between 60 and 440 pmol/l). Taken together, these results indicate that EDCs are specific for the cells expressing the corresponding mAb target and may be capable of sensing distinct NKA interactions that occur upon tumor transformation.

### Mechanism of EDC-mediated cell death and lack of internalization

**Figure 5:**
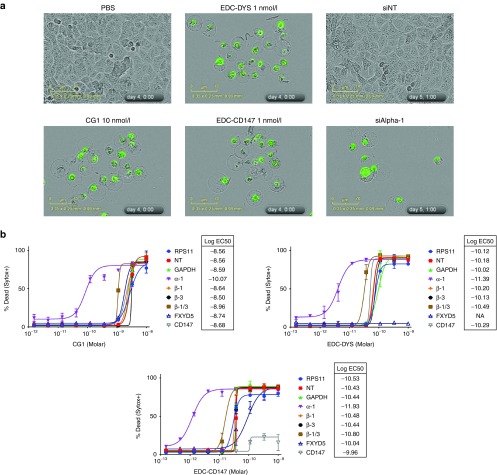
**Mechanism of action**. (**a**) Phase-contrast imaging of A549 cells, in the presence of the membrane impermeable nucleic acid dye Sytox-Green, treated with PBS, CG1, EDC-DYS, EDC-CD147, siRNA against ATP1A1, or nontargeting control siRNA (siNT). (**b**) Cell death dose response curves and EC50 values of A549 cells transfected with the indicated siRNAs and treated with CG1, EDC-DYS, or EDC-CD147. α and β refer to the subunits of the NKA. NA indicates no activity.

Cell morphology studies were conducted to identify and compare the type of cell death induced by EDCs. In the presence of the membrane impermeable dye Sytox Green, A549 cells were treated with EDC-DYS, EDC-CD147, siRNA against α-1 (ATP1A1) or two other CGs (oleandrin and digitoxin) and imaged with phase-contrast microscopy (**Figure 5a** and **Supplementary Figure S2**). The results show that under any of these conditions, cells began to swell within 12–24 hours (3–4 days for siAlpha-1) then partially detached from the substrate with outer membrane rupture occurring in 2–5 days as indicated by staining with the membrane impermeable Syto-Green dye. This swelling phenotype, termed oncosis, followed by membrane rupture is characteristic of necrotic cell death but distinct from apoptotic or autophagic death in which cells shrink and bleb or display cytoplasmic vacuolization, respectively.^34^ Consistently, western blot analysis for apoptosis (Caspase-3 and PARP cleavage) and autophagy (LC3B shift or p62 reduction) markers and immunofluorescence analysis of Annexin V binding, all remained unchanged (data not shown). Similar swelling and membrane rupture was observed when PANC-1, Ramos, SUDHL4 and SUDHL8 cells were treated with EDCs or free CGs (data not shown). These results indicate that like free CGs, EDCs induce necrotic cell death.

To determine whether EDCs internalize or not, we conducted a set of experiments using EDC-DYA and EDC-CD38. First, we observed the trafficking of EDC-DYS and free antibody DYS after addition to H460 cells, an adherent cell line found to be sensitive to EDC-DYS (EC50 = 260 picomolar). Following published procedures, after 3 and 20 hours of incubation, cells were fixed, permeablized and stained with goat anti-mouse IgG Dylight 488.^35^ Fluorescent images showed free antibody and EDC-DYS displaying a pattern of mostly surface staining (bright staining seen along boarders and junctions of the cells (**Supplementary Figure S5A**). We therefore concluded that the antibody surface receptor-dysadherin complexes were poorly internalized upon antibody or EDC-DYS binding. We next observed the trafficking of EDC-CD38 after addition to BF01 cells, a non-adherent cell line known to internalize unconjugated CD38 antibodies.^36^ Following a different published protocol, EDC-CD38 was observed only on the cell surface, even after 20 hours of incubation (**Supplementary Figure S5B**).

### EDC target specificity

We next set out to identify which subunits of the NKA were targeted by the EDCs using siRNA experiments. A549 and PANC1 cells were treated with siRNAs specific to dysadherin, CD147, and each of the four alpha and beta subunits of human NKA Cells were then treated with EDC-DYS, EDC-CD147, or unconjugated CG1 (**Figure 5b** and **Supplementary Figures S3A** and **S3B**). siRNAs against α-1 subunit, but not α-2/3/4 resulted in >10-fold change in the activities of all three treatments. Knockdown of any single β-subunit failed to affect the activities of the treatments while simultaneous knockdown of β-1 and β-3 resulted in a 3-fold change in their activities. Knockdown of dysadherin and CD147 expression resulted in resistance to the corresponding EDC yet yielded no effect on CG1 activity. Knockdown of each expressed isoforms, α-1, β-1, and β-3 and dysadherin was determined by western blot analysis (**Supplementary Figure S4**). Similar results were obtained with Panc1 cells (data not shown). These results suggest that like CG1, EDCs elicit their effects though the α-1 and β-1/3 subunits yet unlike CG1, they were also dependent on the expression of the antibody target.

### *In vivo* EDC efficacy

**Figure 6:**
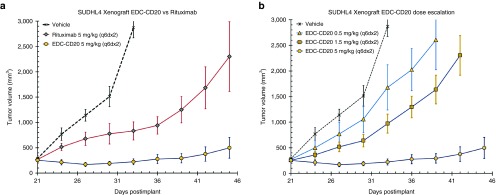
**EDC-CD20 has dose dependent antitumor activity that surpasses Rituximab in DLBCL tumor models.** Data are plotted as group mean tumor volumes post tumor implant, with standard error of the mean (SEM) which are indicated by vertical bars. (**a**) CB17SCID mice (*n* = 6 per group) bearing s.c. SU-DHL-4 xenograft tumors of an average size of 250 mm^3^ were treated with vehicle (dashed line) EDC-CD20 (circle) or Rituximab (diamond) at 5 mg/kg using the dosing schedule of q6dx2 via i.p. injection. (**b**) CB17SCID mice (*n* = 6 per group) bearing s.c. SU-DHL-4 xenograft tumors of an average size of 250 mm^3^ were treated with either vehicle (dashed line) or EDC-CD20 dosed at 0.5 mg/kg (triangle), 1.5 mg/kg (square), or 5 mg/kg (circle) using the dosing schedule of q6dx2 via i.p. injection.

Using human cell lines displaying *in vitro* EDC sensitivity, animal xenograft models were developed and used to evaluate the *in vivo* anticancer activity of three EDCs (**Figure 6** and **Supplementary Figure S6**). Three separate xenograft experiments were conducted to examine EDC-DYS:^1^ direct comparison with CG1,^2^ dose escalation, and^3^ EDC-DYS specificity. Experiments were also devised to directly compare the effects of EDC-CD38 to those of CHOP (a standard chemotherapy cocktail). Finally, a study was conducted to directly compare EDC-CD20 and Rituximab dose responsiveness.

Based on our prior dosing results, a single comparator study using mice bearing human A549 NSCLC tumors was conducted to demonstrate what required dosing would be necessary to obtain similar efficacy for CG1, EDC-DYS and paclitaxel. The results show that the response to EDC-DYS (5 mg/kg administered intravenously (i.v.) every 3 days four times (q3dx4)) was comparable to CG1 (20 mg/kg administered i.v. every 2 days five times (q2dx5)) and paclitaxel (15 mg/kg iv. every day five times (qdx5)) (**Supplementary Figure S6A**). Only the groups receiving CG1 or paclitaxel exhibited mean weight loss >10%. Importantly, each EDC in this report has a drug antibody ratio (DAR) of 4, thus 5 mg of an EDC equates to 0.07 mg of CG1. Therefore, in this study, five administrations of 20 mg of unconjugated CG1 or 100mgs/kg total should be compared to four administrations of 0.07 mg of EDC targeted CG1 or 0.28 mg/kg total. The findings therefore show that in order to achieve a similar response under optimal CG1 dosing, it required 350-fold fewer CG1 equivalents when CG1 was targeted via the EDC approach.

Dose escalation studies were based on the intraperitoneal injection (i.p.) of 1, 5, and 15 mg/kg of EDC-DYS following two distinct schedules into mice bearing human pancreatic cancer tumors (Panc1). Results were compared to a standard dosing regimen of gemcitabine (**Supplementary Figure S6B**). The results clearly show dose dependency, with 15 mg/kg on either a q3dx7 or qwkx3 dosing schedule yielding the strongest response (82 and 60% tumor growth inhibition, respectively). Both of these EDC-DYS dosing regimens outperformed gemcitabine (120 mg/kg q3dx4) showing 42% inhibition of tumor growth.

EDC-DYS specificity was directly compared to the unconjugated antibody (DYS-mAb) and EDC-CONTROL in mice bearing human B-cell tumors (Ramos) averaging 400 mm^3^ in size. Each preparation was administered at 10 mg/kg i.p. on a q5dx3 schedule (**Supplementary Figure S6C**). DYS-mAb and EDC-DYS dosing resulted in tumor growth inhibition of 40 and 87%, respectively. EDC-CONTROL had no effect on tumor growth. These findings suggest that EDC-DYS efficacy is dependent on the presence of both the CG1 and the targeting mAb.

The *in vivo* efficacy of EDC-CD38 was tested and directly compared to CHOP, a standard chemotherapy cocktail. EDC-CD38 (10 mg/kg at q5dx4 i.p.) and CHOP (CHO qdx1 i.p. and P qdx5 orally) were administered to mice bearing human Ramos tumors (**Supplementary Figure S6D**). The administration of EDC-CD38 yielded tumor growth inhibition of 93% as compared to 54% provided by CHOP, indicating that EDC-CD38 was active *in vivo* and the CD38-NKA interaction was preserved.

Finally, the antitumor effects of EDC-CD20 were compared to Rituximab, the standard of care for human B-cell lymphoma. Mice bearing SUDHL4 tumors were administered single 5 mg/kg doses of either EDC-CD20 or Rituximab (**Figure 6a**). EDC-CD20 resulted in a tumor growth inhibition of 99%, superior to the effects of Rituximab at 79%. Furthermore, only EDC-CD20 provided a complete eradication of the tumor burden (2/6 mice showing no palpable tumors on day 45). Finally, EDC-CD20 dose response was evaluated by dosing the models with 0.5, 1.5, or 5 mg/kg (**Figure 6b**). Like the results of the EDC-DYS, a dose response was observed for EDC-CD20.

## Discussion

Antibody-directed drug targeting was first proposed in 1970,^37^ and the idea of attaching potent drugs to antibodies such that they are directed to diseased cells remains largely unchanged.^38^ Reported here are the results of an effort to further develop the ADC paradigm. While EDCs are similar to ADCs in basic composition, their novelty lies in the ability to act exclusively on extracellular cell surface proteins, requiring neither internalization nor breakdown of the conjugate. Several of the experiments conducted in this study yielded data in support of this assertion. First, during the construction of the initial EDCs, data demonstrated that linker length factored significantly and specifically to EDC activity. Second, antibodies to proteins either known to interact or hypothesized to interact with the NKA yielded potent EDCs. Third, the active EDCs never exhibited activity above background when tested on cell lines not expressing their antibody's target. This was true even after all cell lines were determined to be sensitive to unconjugated free drug CG1. Studies to further investigate the putative plasma membrane associated protein-protein interactions are ongoing. The results also indicate that EDCs do not require internalization or breakdown. First, all active EDCs target two extracellular regions of membrane bound proteins and therefore should not necessitate internalization. Second, since antibodies specific to poorly-internalizing targets such as CD20 can render potent EDCs,^31,39^ internalization of the antibody is not required. Third, the data presented showed a strong correlation between EDC linker length and EDC activity (EDCs with longer linkers were more active). If EDCs required breakdown, one would presume that linker length would not greatly affect activity, especially given the data showing that linker-CG1 constructs (void the antibody) displayed more activity when the linkers were shorter. Forth, the data presented shows that neither EDC-DYS nor EDC-CD38 are actively internalized. Taken together, these findings support the notion that EDCs require neither internalization nor breakdown. This is an important finding because it could add additional targets to the current arsenal of safe and effective antibody drug conjugates as today's ADCs require internalization. Not requiring internalizing may also be beneficial in the fight against cancer as many drug resistant pathways involve the upregulation of ATP-binding cassette (ABC) transporters, which include plasma membrane pumps that extrude toxins and drugs out of the cell. A noninternalizing EDC should therefore not be affected by such pathways.

The results also indicate that even when freshly prepared normal cell types expressing both EDC targets were tested, they were much less sensitive to EDC affects than cancer cells. This was never the case for unconjugated free drug CG1. One simple explanation for this would be that the EDC specific proteins simply do not interact or the interaction occurs less frequently on normal cells, a situation that could be due to surface protein expression levels, protein trafficking differences, lipid raft micro-domains or other cell surface character variations. Whatever the reason, it may provide a unique opportunity to selectively target cells existing in a diseased state and further increase the TI, which has hindered the clinical progress of CGs for indications beyond heart disease. This now needs to be confirmed in cancer patients.

The *in vivo* efficacy results provide support that EDCs act in a dose- and antibody-dependent fashion. Furthermore, responses achieved by EDC technology required 350-fold fewer CG1 equivalents to achieve results similar to unconjugated free drug CG1. The findings suggest that protein-protein interactions between the NKA and the other EDC-targeted proteins are maintained and readily accessible in *in vivo* models. The data also indicates that Rituximab's ability to bind CD20 can be used to target a potent inhibitor of the NKA with EDC technology. Finally, the data presented show that like cardiac glycosides, EDCs act by inhibiting the NKA, thus far an untapped mechanism of action for cancer patient therapy. This suggests that EDCs may be useful in treating cancers, especially multi-drug-resistant cancers. Efforts geared toward this possibility are ongoing.

Each of the five cell surface proteins targeted by the potent EDCs discussed have cancer-related functions. Expression of dysadherin, the NKA gamma 5 subunit, has previously been shown to be a prognostic indicator for late-stage metastatic cancers.^40^ CD20 is best known as being a target for Rituximab and other clinically approved antibody therapies. The CD38 target is gaining attention from recent approval of Daratumumab (Darzalex; Janssen Biotech, Horsham, PA), the first clinically approved antibody specific to human CD38 for the treatment of multiple myeloma. The CD56 target is a marker for natural killer cells, certain forms of cancer, is associated with cell migration and invasion, and is the target for the ADC known as IMGN901.^41,42^ Finally, CD147 has been linked to a number of important cancers and has been found to induce the expression of matrix metalloproteinases as well as the lactate transporters MCT1 and MCT4.^43^ Clearly more targets will be identified as this work progresses but the list so far shows great promise at targeting CGs and other molecules that act on extracellular membrane targets.

In conclusion, for the first time, the results of this study highlight a new and effective paradigm for antibody-directed targeting of small molecules. The new molecules highlighted are termed extracellular drug conjugates or EDCs because they do not require internalization or breakdown. The results also bring a new level of precision to cancer targeting showing that EDCs depends on the proximity effects of two targets and not simply the expression of those targets. The research presented here also provides a framework upon which future research initiatives can be delineated and structured with the end goal being the development of precision therapies capable of selectively targeting and safely treating diseased tissues.

## Materials and Methods

***Reagents and antibodies.*** Reagents and solvents were used *sans* further purification unless otherwise specified Polyethylene glycol-derivatives (Quanta Biodesign, Plain City, OH), Proscillaridin-A (Molekula, Gillingham, UK), *N*-phthalimido-tetracetyl-glucosamine (Carbosynth, Berkshire, UK), *p*-nitrophenyl phosphate (Pierce Biotechnology, Waltham, MA), and tri(2-carboxyethyl)phosphine hydrochloride (Hampton Research, Aliso Viejo, CA). Antibodies used: Mouse IgG1 α-dysadherin clone NCC-M53 (National Cancer Center, Tokyo, Japan), mouse IgG1 α-EMMPRIN clone 8D12 (eBiosciences, San Diego, CA), mouse IgG1 α-NCAM clone HCD56 (Biolegend, San Diego, CA), mouse IgG1 α-CD29 clone MEM-101A (Biolegend), mouse IgG1 α-CD47 clone CC2C6 (Biolegend), mouse/human chimeric IgG1 α-CD20 Rituximab (Genentech, South San Francisco, CA), IgG1 α-CD38 clone SUN4B7 (F. Malavasi, Torino, Italy). Human IgG Protein-A purified from normal serum (Innovative Research, Novi, MI) was used make EDC-Control. Antibodies used for western blotting were; ab175213, ab181602, ab76020, ab2873, and ab137055 (Abcam, Cambridge, MA).

***Synthesis of CG1 antibody conjugates.*** **A**ntibody conjugates were prepared by reduction-alkylation of antibody inter-chain disulfides. Briefly, between 1–10 mg/ml of antibody in dulbecco's phosphate-buffered saline (DPBS) was reduced in the presence of 1 mmol/l diethylenetriaminepentaacetic acid (DTPA) and 8 molar equivalents of tris(2-carboxyethyl) phosphine (TCEP) at 37 °C for 2 hours. These mixtures were then cooled in an ice bath, at which time 9.6 molar equivalents of a maleimide activated CG PEG compound (CG1 PEG2, CG1 PEG12, CG1 PEG24, or CG1 PEG36) was added and allowed to react for 30 minutes on ice. Residual maleimide groups were then quenched by the addition of a 1.5 equivalent excess of L-cysteine (based upon maleimide), which was allowed to react for 30 minutes at room temperature (RT). Resulting antibody conjugates were separated from Cys-capped reagents by forced dialysis using Amicon Ultra 30,000 molecular weight cut-off (MWCO) centrifugal filters (Millipore) and DPBS buffer exchange (3×). Conjugates were stored (4 ^°^C) in DPBS at concentrations ranging from 1–10 mg/ml.

***DAR calculation.*** Drug antibody ratios were determined by measuring absorbance of conjugates, antibodies (Ab) and free drug (drug) at both 280 and 299 nm. Next, the following constants were determined; [Constant Ab] = A299Ab/A280Ab; [Constant Drug] = A299drug/A280drug. Next, absorbance of antibody drug conjugate was measured [A280 and A299]. Drug loading = drug concentration/antibody concentration. Using the following, drug loading was determined. A280Ab = A280 – (A299 – [Constant Ab] × A280)/([Constant drug] – [Constant Ab]). A299drug = A299 – [Constant Ab] × A280Ab. Antibody concentrations = A280Ab/204,000 M-1cm-1 (varies depending on antibody). Drug concentration = A299drug/5623 M-1cm-1. A299drug = drug component. A280Ab = antibody component.

***Cell lines.*** A375, A549, BF01, FaDu, H520, H69, HT29, MRC-9, PANC-1, Ramos, RPMI-8226, SU-DHL-4, SU-DHL-8, U937 (ATCC), and LOX-IMVI (NCI) cells were grown in ATCC/NCI recommended media supplemented with 50 µg/ml gentamycin (GIBCO). HUAEC and HREpC cell lines were maintained in Endothelial Cell Growth Medium 2 and Renal Epithelial Cell Growth Medium 2 (PromoCell GmbH). All cells were grown at 37 °C in a humidified atmosphere with 5% CO_2_.

***PBMC testing.*** PBMC from healthy donors were immediately plated (2 × 10^5^/ml) in Roswell Park Memorial Institute (RPMI) 1640 medium plus 10% fetal calf serum (FCS) and treated with EDC for 20 hours. Cells were then collected and stained with fluorescein isothiocyanate (FITC)-conjugated annexin-V (BioLegend) and propidium iodide (Invitrogen) for 15 minutes at room temperature. Apoptosis was measured using a FACSCanto flow cytometer (Becton-Dickinson) and evaluated with the FlowJo software.

***Fluorescent antibody staining.*** Cells were stained with 1 µg/ml of primary antibodies and 5 µg/ml goat anti-mouse IgG, DyLight 488 (Pierce Biotechnology) in DPBS with 1.5% fetal bovine serum (FBS) for 30 minutes, washed twice, and imaged using a Nikon Diaphot-TMD inverted fluorescence microscope. PBMC from healthy donors were plated (2 × 10^5^/ml) in RPMI 1640 10% FCS and treated with EDC for 20 hours, collected, and stained with FITC-conjugated Annexin-V (BioLegend) and propidium iodide (Invitrogen) for 15 minutes at room temperature. Apoptosis was measured using a FACSCanto flow cytometer (Becton-Dickinson) and evaluated with the FlowJo software. Early apopstosis was defined as Annexin V-positive, PI-negative and late apoptosis as Annexin V-positive, PI-positive.

***Cell proliferation/cytotoxicity assays.*** For Cell Titer-Glo assays, cells were treated 24 hours after plating and Cell Titer-Glo Luminescent Cell Viability Assay (Promega) was performed 72 hours later. Luminescence measurements were performed using a Wallac Victor^2^ Model 1420-041 assay plate reader (Perkin Elmer, Gaithersburg, MD). EC_50_ values for each test agent were determined with GraphPad Prism 5 software (GraphPad).

***Cell morphology analysis.*** A549 cells (~ 1,500) were plated in 96-well plates in full media containing 125 nmol/l Sytox Green, treated as indicated with EDCs/steroidal glycosides after 24 hours, and imaged by phase-contrast with an Incucyte Zoom (Essen Biosciences) using a 20× objective. Images were adjusted using Incucyte Zoom software (Essen Biosciences).

***siRNA combination studies.*** A549 cells (~370) were reverse transfected in 384-well plates with 0.2, 0.12, or 0.04 picomoles of total Dharmacon Smartpool siRNAs and 0.06 μl of RNAiMax transfection reagent per well in full media containing 125 nmol/l Sytox Green if needed for analsysis. After 24 hours, EDCs/steroidal glycosides were added and cells were imaged over a period of 5 days using the Incucyte Zoom (Essen Biosciences) or after 72 hours Cell Titer-Glo Luminescent Cell Viability Assay was performed. Percent confluence at 5 days was determined using Incucyte Zoom Software (Essen Biosciences). At the end of 5 days, Triton X100 (1% final concentration) was added to each well and plates were rescanned to determine the total number of cells per well. Percent dead was calculated by dividing the number of Sytox Green-positive cells before Triton X100 addition the total number per well. Percent confluence and percent dead data were analyzed and EC50 curves were plotted using GraphPad Prism 5 software (GraphPad).

***EDC internalization studies.*** Experiments were performed following Polson *et al*.^35^ Briefly, H460 cells (large cell lung cancer) were seeded into 96-well, clear bottom, tissue culture treated plates at a density of 10,000 cells/well in 100 µl media (RPMI 1640 with 10% fetal bovine serum and 50 µg/ml Gentamicin)/well. Cells were incubated at 37 °C and 7% CO2 for 24 hours. NCC-M53 and EDC-DYS were diluted in media, with and without protease inhibitors (10 µg/ml leupeptin, and 5 µmol/l pepstatin), to a final concentration of 2 µg/ml added to wells (100 µl/well) at time points −21 or −3 hours. At time 0 hours, media was removed by aspiration and the wells were washed 1× with DPBS + 1.5% FBS. Cells were then fixed with 4% formaldehyde in phosphate buffered saline (PBS) for 20 minutes at room temperature and permeabilized with 0.1% Triton X-100 in PBS for 15 minutes at room temperature. For controls, cells that did not receive antibody during culture were stained with M53 or M53-PEG24-CEN09-106 after fixation and permeabilization. Cells were then washed twice with DPBS + 1.5% FBS. Goat anti-mouse IgG Dylight 488 conjugate (Thermo) secondary was diluted to 5 µg/ml in DPBS + 1.5% FBS and added to all wells at 100 µl/well and incubated for 30 minutes at room temperature. Cells were again washed, and then viewed with Nikon Diaphot TMD inverted trinocular fluorescence phase contrast microscope with Ph2 20X DL objective. Pictures were captured using an Olympus E-450 Digital SLR camera, and the brightness of fluorescence images were adjusted using GIMP 2.6 (GNU Image Manipulation Program, The GIMP Development Team (International)).

***Xenograft studies*** *General.* EDCs were diluted in sterile saline for injection and dosed as indicated. Paclitaxel (LC laboratories) was dissolved as a 15 mg/ml in ethanol/Cremophor EL (1:1) stock solution, and stored at −80 °C. This stock solution was diluted 1.5 mg/ml in saline just before injection at 10 µl per gram of mouse body weight. Gemcitabine (LC laboratories) in saline stored at room temperature and dosed at 120 mg/kg. CHOP consists of a single intraperitoneal injection of 30 mg/kg cyclophosphamide (Sigma-Aldrich), 2.475 mg/kg doxorubicin (LC Laboratories), and 0.375 mg/kg vincristine (LC Laboratories), in addition to oral dosing of 0.15 mg/kg prednisone (Sigma-Aldrich) once a day for 5 days. Tumor sizes were calculated using the formula: Tumor volume (mm^3^) = (w2 × l)/2 where w = width and l = length in mm of a given tumor.

*Non–small-cell-lung cancer xenograft.* A549 tumor line was maintained by serial subcutaneous transplantation in athymic nude mice. A549 tumor fragments (~ 1 to 2 mm^3^ each) were implanted subcutaneously via trocar into the left flank of female HRLN nu/nu mice of 8–12 weeks old (Harlan Laboratories). Animals were randomized (six mice per group; four groups) and treatment initiated when tumors reached an average volume of 100 mm^3^. Tumor-bearing mice were dosed as shown in **Figure 4**. Every 4 to 7 days tumor volume was assessed for each group using calibrated vernier calipers and results were plotted against time from day of first dose.

*Pancreatic cancer xenograft.* PANC-1 tumor cells were maintained by serial subcutaneous transplantation in athymic nude mice, and PANC-1 tumor fragments (1 mm^3^) were implanted subcutaneously into the right flank of female HRLN nu/nu mice of 8–12 weeks old (Harlan Laboratories). Mice bearing tumors of 90–130 mm^3^ were randomized by tumor size and segregated into nine groups (*n* = 10 mice/group). Group mean tumor volumes ranged from 106–112 mm^3^ and individual tumor volumes from 63–172 mm^3^. Tumor-bearing mice were dosed as shown in **Figure 3**. Twice per week tumor volume was assessed for each group using calibrated vernier calipers and results were plotted against time from day of first dose.

*B-cell lymphoma xenograft.* Ramos and SU-DHL-4 cells were washed, suspended in Dulbecco's PBS (Hyclone), and inoculated subcutaneously (1 × 10^7^ cells suspended in 0.2 ml per mouse) into the flanks of female SHO mice 28 to 35 days old (Ramos) and CB17SCID mice 36–42 days old (SU-DHL-4) (Charles River Laboratories). When mean tumor size reached ~250 mm^3^, mice were divided into groups (five mice/group- Ramos; six mice/group- SU-DHL-4) with the same mean tumor size and dosed by intraperitoneal injection.

*General analytical chemistry.* ^1^H-NMR and ^13^C-NMR analyses were performed using a Varian Mercury Plus 300 MHz NMR spectrometer (Department of Chemistry, UW-Madison). ESI-MS spectra were obtained using an AB Sciex QTRAP, and ESI-HRMS spectra were generated with an Agilent LC/MSD TOF instrument. All samples (in methanol or 1:1 acetonitrile-water) were injected into the electrospray source at a rate of 30 µl/minute. MALDI-MS spectra were obtained with an ABI Sciex 4800 TOF/TOF mass spectrometer using α-cyano-4-hydroxycinnamic acid as an ionization matrix (Biotechnology Center of the UW-Madison). high performance liquid chromatography (HPLC) analyses were performed on a Waters modular system consisting of a Delta 600 fluid handler, a 600 controller, a 2777 auto sampler, and a 2996 PDA detector. For analytical HPLC, a Gemini C18 5 µm, 110 Å, 250 × 4.6 mm column was used with a 100 µl injection loop. Semi-preparative HPLC was performed on the same system via manual injection with a 500 µl injection loop and Gemini C18 5 µm, 110, 250 × 10.0 mm column. TLC analysis was performed using EMD glass-backed TLC plates precoated with a 0.25 mm layer of silica gel 60 F_254_ (EMD Chemicals, Gibbstown, NJ). TLC protocols were developed based on one or more of the following detection techniques: (i) UV at 254 nm, (ii) iodine vapors, and (iii) a solution of 2.5 g phosphomolybdic acid, 1.0 g ceric sulfate, 6.0 ml sulfuric acid, and 94 ml water, followed by heating. Flash chromatography was performed with a Biotage SP4 flash chromatography instrument (Charlottesville, VA) using Biotage cartridges. Absorbance values for the antibodies and **CG1** were determined on a Beckman DU530 UV/VIS spectrophotometer.

*Synthesis of CG1 and PEG derivatives: 1-*O*-allyl-α-D-arabinopyranoside 8.* D-Arabinose **7** (100 g, 666 mmol) and Na_2_SO_4_ (100 g, 703 mmol) were suspended in allyl alcohol (1 l, 14.65 mol) at RT. To this suspension was added concentrated H_2_SO_4_ (10.4 ml, 187 mmol). The mixture was stirred overnight at 85 °C. The solids were removed by filtration and washed with allyl alcohol (2 × 300 ml). The combined filtrate was concentrated *in vacuo*. The crude material was purified by flash chromatography (silica gel, CH_2_Cl_2_ to CH_2_Cl_2_-MeOH 80:20) to give **8** as an off-white solid (75 g, 60%).

*1-O-allyl-2,3-di-O-benzoyl-α-D-arabinopyranoside 9.* To a solution of **8** (75 g, 394 mmol) in pyridine (800 ml) at 0 °C was added benzoyl chloride (101 ml, 867 mmol) over 2 hours. The reaction mixture was stirred at RT for 16 hours. The solvent was removed *in vacuo*. The crude material was purified by flash chromatography (silica gel, hexanes-EtOAc 8:2 to 6:4) to give **9** as a thick oil (45 g, 29 %).

*1-O-allyl-2,3-di-O-benzoyl-4-O-trifluromethanesulfonyl-α-D-arabinopyranoside 10.* To a solution of **9** (20.0 g, 50 mmol) in dry CH_2_Cl_2_ (100 ml) and pyridine (16.2 ml, 200 mmol) at 0 °C was added triflic anhydride (10.6 ml, 62.7 mmol). The reaction mixture was stirred at 0 °C for 15 minutes. CH_2_Cl_2_ (500 ml) was added to the solution and the organic layer was washed with cold 1N HCl (200 ml), saturated NaHCO_3_ (200 ml) and brine (200 ml). Organics were then dried (Na_2_SO_4_), and concentrated *in vacuo* to give crude **10** as a yellow oil. The crude material was carried on without further purification.

*1-O-allyl-2,3-di-O-benzoyl-4-deoxy-4-azido-α-L-xylopyranoside 11.* Crude **10** (26.6 g, 50.1 mmol) was dissolved in *N,N*-dimethylacetamide (100 ml) and sodium azide (6.37 g, 98 mmol) was added to the solution. The mixture was stirred overnight at RT. The solvent was removed *in vacuo*, and the residue was dissolved in CH_2_Cl_2_ (750 ml). The organic layer was washed with water (2 × 200 ml) and brine (200 ml), dried (Na_2_SO_4_) and concentrated *in vacuo*. The crude material was purified by flash chromatography (silica gel, hexanes:EtOAc 8:2 to 6:4) to give **11** as a thick colorless oil (17.5 g, 84 %).

*2,3-Di-O-benzoyl-4-deoxy-4-azido-L-xylopyranoside 12.* To a solution of **11** (15.7 g, 37.1 mmol) in CH_2_Cl_2_-MeOH (100 ml, 90:10) under argon was added PdCl_2_ (0.66 g, 3.7 mmol). The reaction mixture was stirred overnight at RT, filtered through a pad of celite and concentrated. The crude material was purified by flash chromatography (silica gel, hexanes-EtOAc 8:2 to 5:5) to give **12** as a thick colorless oil and as 2:1 mixture of α/β (11.1 g, 78 %).

*2,3-di-O-benzoyl-4-deoxy-4-azido-α-L-xylopyranoside 1-trichloroacetimidate 13.* To a solution of **12** (11.1 g, 28.9 mmol) in dry CH_2_Cl_2_ (180 ml) under argon was added trichloroacetonitrile (29 ml, 289.5 mmol) at 0 °C, followed by DBU (0.8 ml, 5.2 mmol). The reaction mixture was stirred at 0 °C for 1 hour. The solvent was removed *in vacuo*. The crude material was purified by flash chromatography (silica gel, hexanes-EtOAc 9:1 to 8:2) to give **13** as a yellow oil (14.04 g, 92%).

*Scillarenin 14.* To a solution of proscillaridin A (3 g, 5.6 mmol) in ethanol (60 ml) at 40 °C was added sodium acetate buffer (170 ml, 0.02 M, pH 4.0), followed by naringinase (1.03 g, Sigma-Aldrich, Cat# N1385). The reaction mixture was stirred overnight, diluted with ethanol (200 ml), filtered through a pad of Celite and the filtrate was concentrated *in vacuo*. The residue was purified by flash chromatography (silica gel, CH_2_Cl_2_-MeOH 98:2 to 90:10) to give **14** as an off-white solid (1.9 g, 90%).

*Scillarenin-2',3'-di-O-benzoyl-4'-deoxy-4'-azido-β-L-xylopyranoside 15.* A solution of **13** (14.04 g, 26.6 mmol) in dry CH_2_Cl_2_ (100 ml) was added to a suspension of activated 4 Å molecular sieves (2 g) in dry CH_2_Cl_2_ (100 ml) under argon at 0 ^°^C. Scillarenin (**14,** 10.2 g, 26.6 mmol) was then added to the mixture. After 10 minutes of stirring at 0 °C, Zn(OTf)_2_ (0.96 g, 2.66 mmol) was added. The reaction mixture was stirred at 0 °C for 2 hours. The reaction was quenched with Et_3_N (3.7 ml, 26.6 mmol). The mixture was filtered and the solvent was removed *in vacuo*. The crude material was purified by flash chromatography (silica gel, hexanes-EtOAc 7:3 to 1:1) to give **15** as a white powder (15.93 g, 80%).

*Scillarenin-4'-deoxy-4'-azido-β-L-xylopyranoside 16.* A solution of **15** (15.66 g, 20.9 mmol) in MeOH-CH_2_Cl_2_ (590 ml, 75:25) was cooled to 0 °C. NaOMe (25% in MeOH, 2.1 ml, 10.3 mmol) was subsequently added and the reaction mixture stirred at RT for 1.5 hours. The reaction was quenched with acetic acid (0.6 ml, 10.3 mmol) and the crude material was purified by flash chromatography (silica gel, CH_2_Cl_2_-MeOH 98:2 to 90:10) to give **16** as a white powder (2.6 g, 23%).

*Scillarenin-4'-deoxy-4'-amino-β-L-xylopyranoside (CG1).* To a solution of **16** (2.6 g, 4.8 mmol) in THF-H_2_O (50 ml, 92:8) was added PPh_3_ (2.5 g, 9.6 mmol). The reaction mixture was stirred at 45 °C for 2.75 hours. The mixture was then added dropwise to Et_2_O at 0 °C. The resulting precipitate was filtered and washed with Et_2_O and dried in air to give **1** as a slightly yellow powder (2.0 g, 81)

*Compound CG1 PEG12.* To a solution of **1** (36 mg, 0.069 mmol) and maleimide-PEG_12_-NHS ester (50 mg, 0.058 mmol) in *N,N*-dimethylacetamide (1 ml) was added Et_3_N (0.08 ml, 0.577 mmol). The reaction mixture was stirred at RT for 1 hour and solvent subsequently removed *in vacuo*. The crude material was purified by flash chromatography (silica gel, CH_2_Cl_2_-MeOH 95:5 to 80:20) to give **3a** as a slightly yellow oil (30 mg, 41%).

*Compound CG1 PEG24.* To a solution of **1** (250 mg, 0.485 mmol) and maleimide-PEG_24_-NHS ester (676 mg, 0.485 mmol) in N,N-dimethylacetamide (1 mL) was added Et_3_N (0.26 mL, 1.45 mmol). The reaction mixture was stirred at RT for 1 hour. The solvent was removed *in vacuo*. The crude material was purified by HPLC.

**SUPPLEMENTARY MATERIAL** **Figure S1.** Effects of drug linker length. **Figure S2.** Time course of cell response to various treatments. **Figure S3.** Dose response curves for various siRNA treated A549 cells. **Figure S4.** Western blot analysis of proteins after siRNA treatments. **Figure S5.** Data showing EDC target complex does not internalize. **Figure S6.** Xenograft study analysis graphs. **Table S1.** Linker lengths optimization study. **Table S2.** mAb target presence and EDC EC50 values resulting from various treatments on cancerous and normal cell lines. **Supplementary Data**
