# Supplementary material for: Extracellular Antibody Drug Conjugates Exploiting the Proximity of Two Proteins
Source: Mol Ther. 2016 Jul 19;24(10):1760–70. doi: 10.1038/mt.2016.119 (PMC5112037; doi:10.1038/mt.2016.119)
Supplement: Supplementary Figures and Tables [file mt2016119x1.pdf]

Supplementary Figures and Tables

Extracellular Antibody Drug Conjugates Exploiting the Proximity of Two Proteins

David J. Marshall[1], Scott Harried[1], John Murphy[1], Chad Hall[1], Mohammed Saleh Shekhani[x], Christophe Pain[x], Conner Lyons[x], Jon S. Thorson[2], Antonella Chillemi[3], Fabio Malavasi[3], Homer L. Pearce[4], and James R. Prudent[1*]

[1] Centrose, 918 Deming Way, Madison, Wisconsin 53717, USA

[2] Center for Pharmaceutical Research and Innovation, University of Kentucky College of Pharmacy, 789 South Limestone Street, Lexington, KY 40536-0596

[3] Laboratory of Immunogenetics, Department of Medical Sciences, University of Torino, Torino, Italy.

E-mail: prudent@centrosepharma.com; Tel: (608) 836-0207

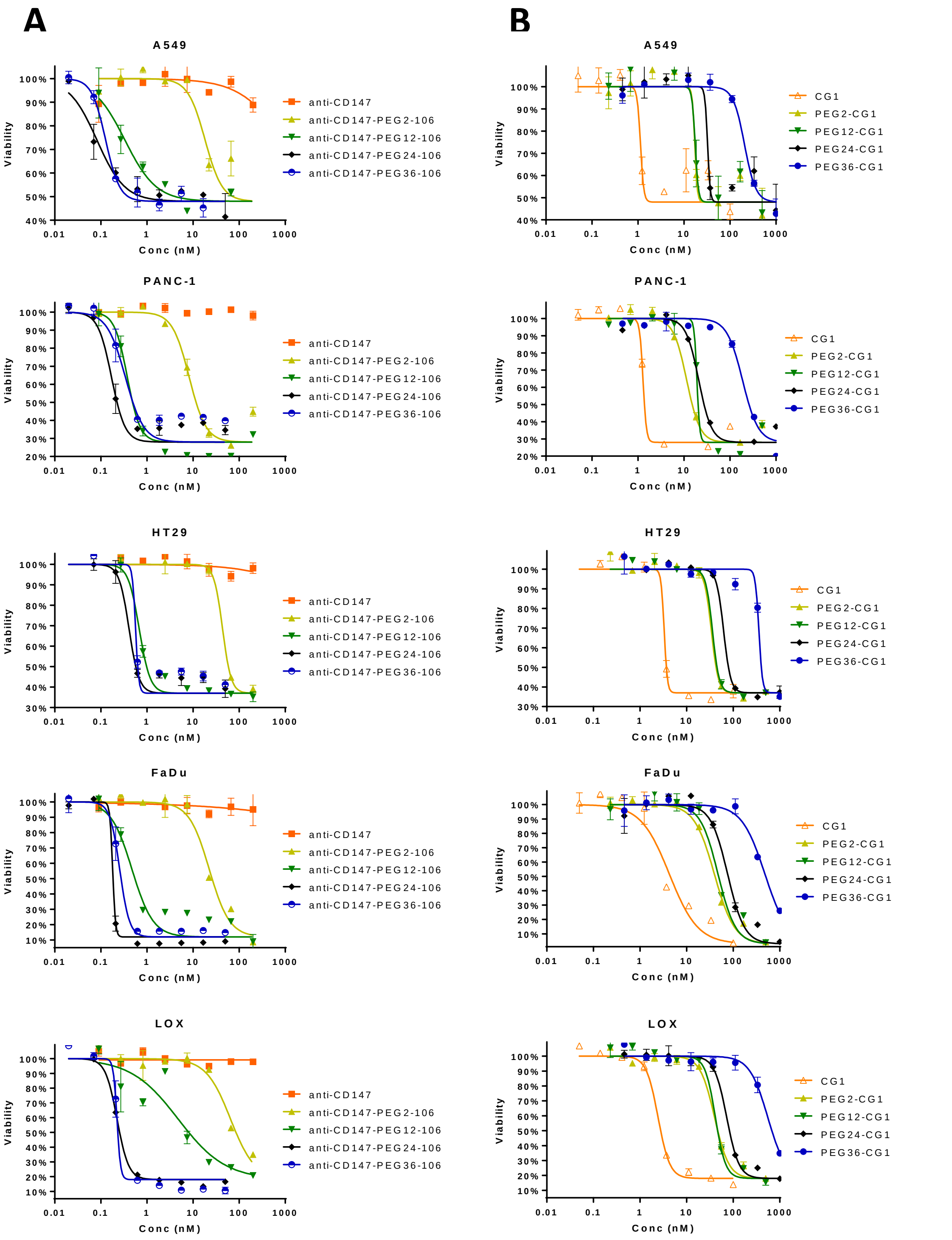

Supplemental Figure 1A and 1B. Effects of linker length on EDC-CD147 and CG1-Linker. Dose response curves of five different cancer cell types (A549/NSCLC, PANC-1/pancreatic, HT29/colon, FaDu/head and neck, and LOX/melanoma) treated with either (**A**) EDC-CD147s or (**B**) CG1 and CG1-Linkers, that only differ by linker length. Note that in panel A., EDCs possessing longer linkers are more active while in panel B., CG1 with longer linkers are less active.

C.

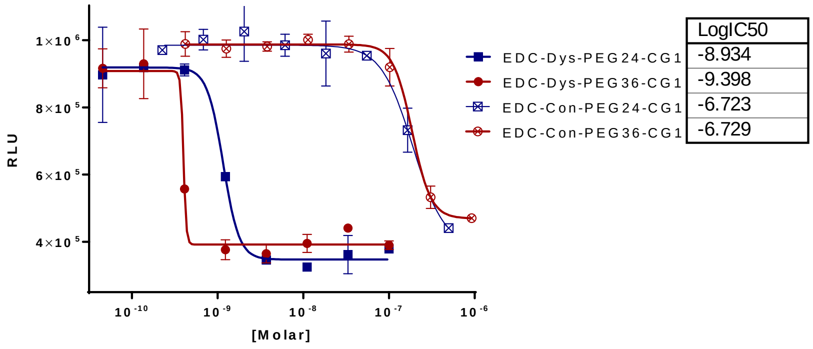

D.

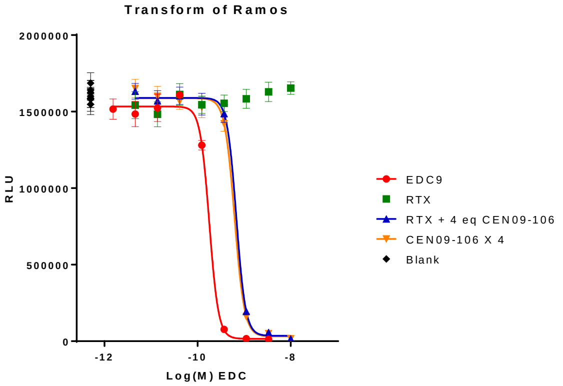

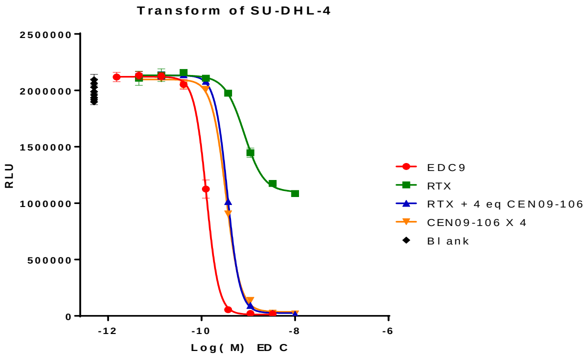

	IC50
EDC9	1.776e-010
RTX + 4 eq CEN09-106	6.765e-010
CEN09-106 X 4	6.194e-010

	IC50
EDC9	1.270e-010
RTX	8.174e-010
RTX + 4 eq CEN09-106	3.567e-010
CEN09-106 X 4	3.339e-010

Supplemental Figure 1C. Effects of linker length on EDC-DYS and EDC-CONTROL. Dose response curves and EC_{50} values of A549 cells treated with EDC-DYS or EDC-CONTROL that either possess PEG24 or PEG36 linkers. Notice that EDC-DYS's are considerably more active than the EDC-CONTROLs and that linker length does not change the activity of the CONTROLS.

Supplemental Figure 1D. Comparative Analysis of EDC-CD20 to CG1 plus Rituximab. Dose response curves and EC50 values of Ramos and SUDHL4 cells treated with EDC-CD20, Rituximab (RTX), CG1 or a combination of RTX and CG1. Notice that EDC-CD20 is more active than either CG1 alone or when it is combined with RTX. Also notice that RTX does not add to the cytotoxic effects of CG1 when the two are combines even when the cells are sensitive to RTX alone as in the case for SUDHL4 cells. Four equivalents of CG1 were used for the experiments since EDC-CD20 possesses a DAR =4.

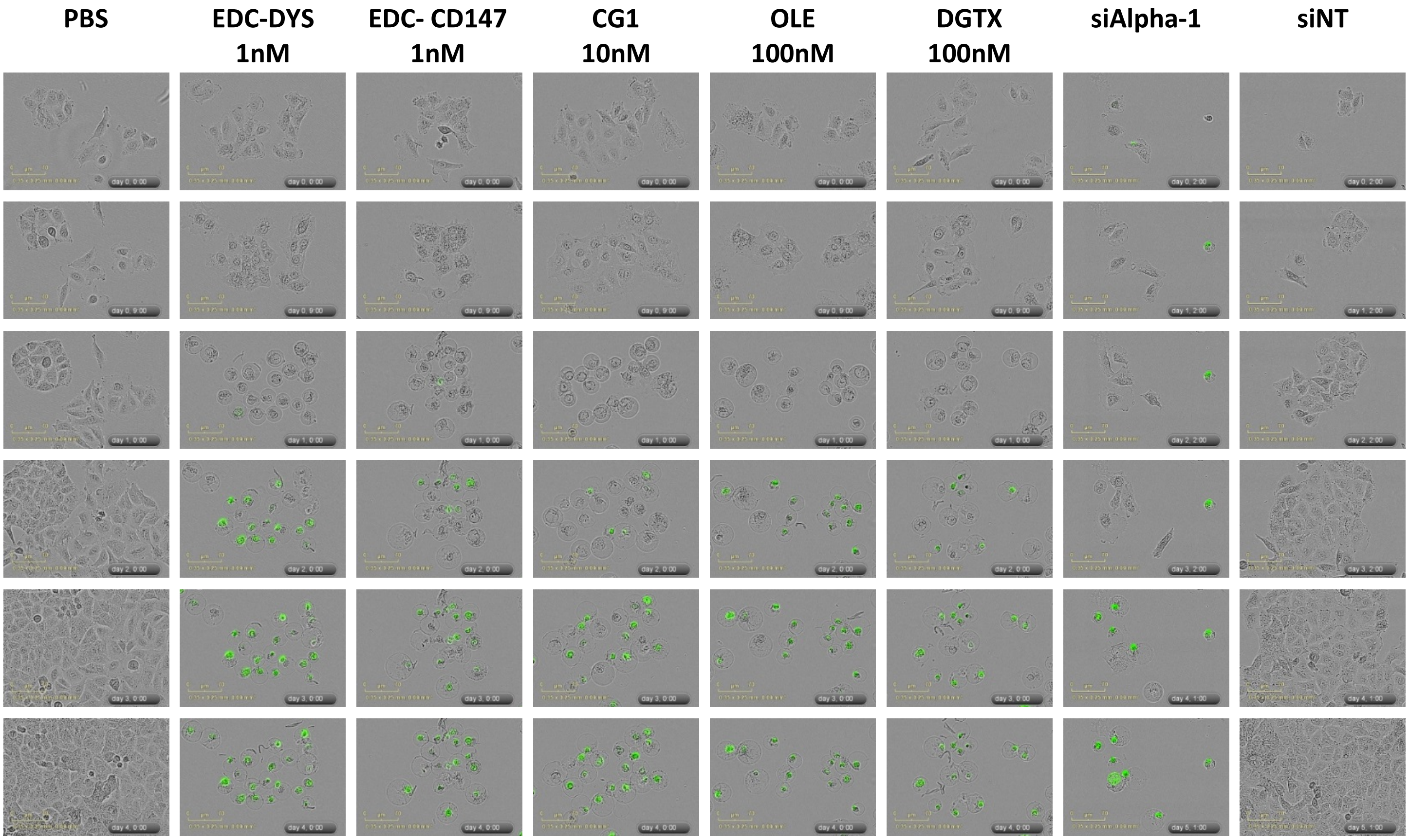

Supplemental Figure 2. **Time course of cell response to various treatments.** Phase contrast imaging of A549 cells in the presence of Sytox-Green that were untreated or treated as indicated during post treatment times of: 0 hours, 9 hours, 1 day, 2 days, 3 days, and 4 days.

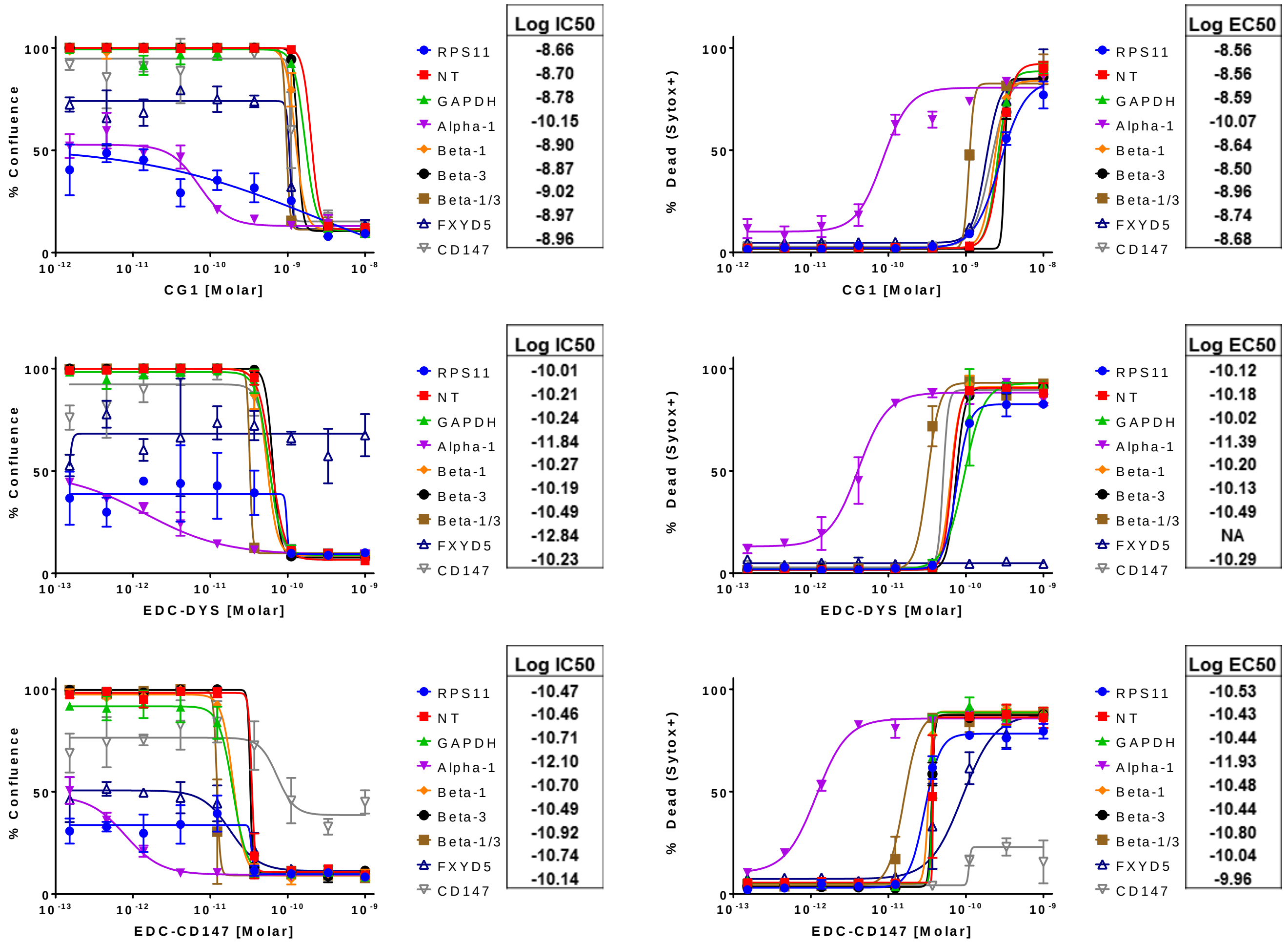

Supplemental Figure 3A. **Dose response curves of siRNA treated A549 cells.** Data shows either percent confluence (left) as determined by phase contrast microscopy or percent dead (right) as determined by fluorescent microscopy using Sytox Green. Graphs show EC_{50} values in log molar of siRNA transfected cells treated with either CG1, EDC-DYS, or EDC-CD147 as indicated. NA = no activity. NT= non-targeting siRNA control. To all cells, 0.2 picomoles of Dharmacon Smartpool siRNAs were added.

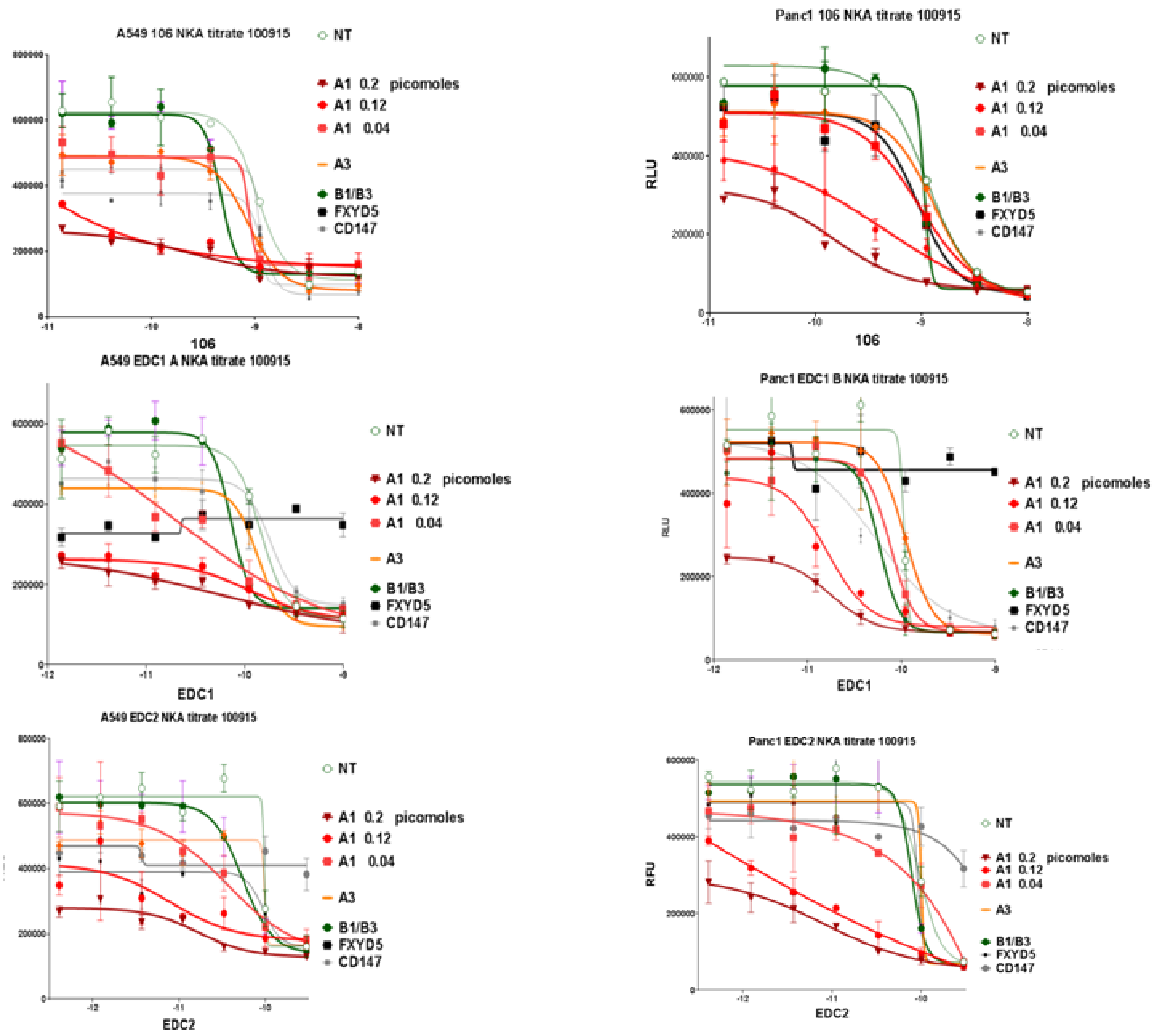

Supplemental Figure 3B. **Dose response curves of alpha1 siRNA treated A549 (Left) and PANC1 (Right) cells.** Graphs show the dose response of cells treated with either CG1, EDC1 or EDC2 in Relative Fluorescent Units (RLU) as determined by CellTitre Glo analysis 72 hours after dosing. Alpha1 specific siRNA pools were added in amounts indicated, 0.2, 0.12 or 0.04 picomoles and for all others, 0.2 picomoles were added. See: Supplementary Data Materials and Methods Section.

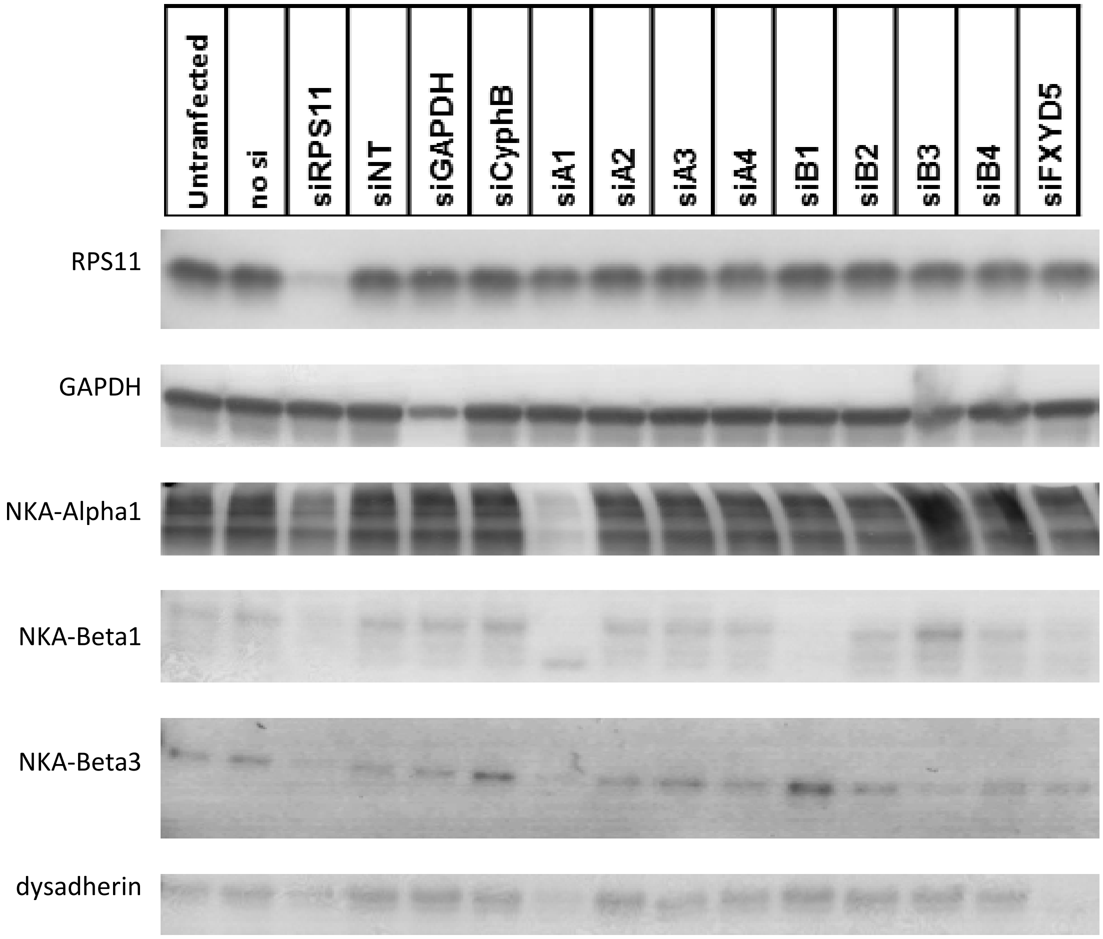

Supplemental Figure 4. **Western blot analysis of proteins after siRNA treatments.** Expression and knockdown efficiency of siRNAs in PANC1 cells after 48 hours post transfection are indicated by antibody staining indicated on left and siRNA specific target along the top. Note the requirement of A1 for full expression of B1, B3, and dysadherin and the apparent reciprocal regulation between B1 and B3. Also note diminished expression of some proteins in siRPS11, likely due to inhibition of global translation by this lethal siRNA.

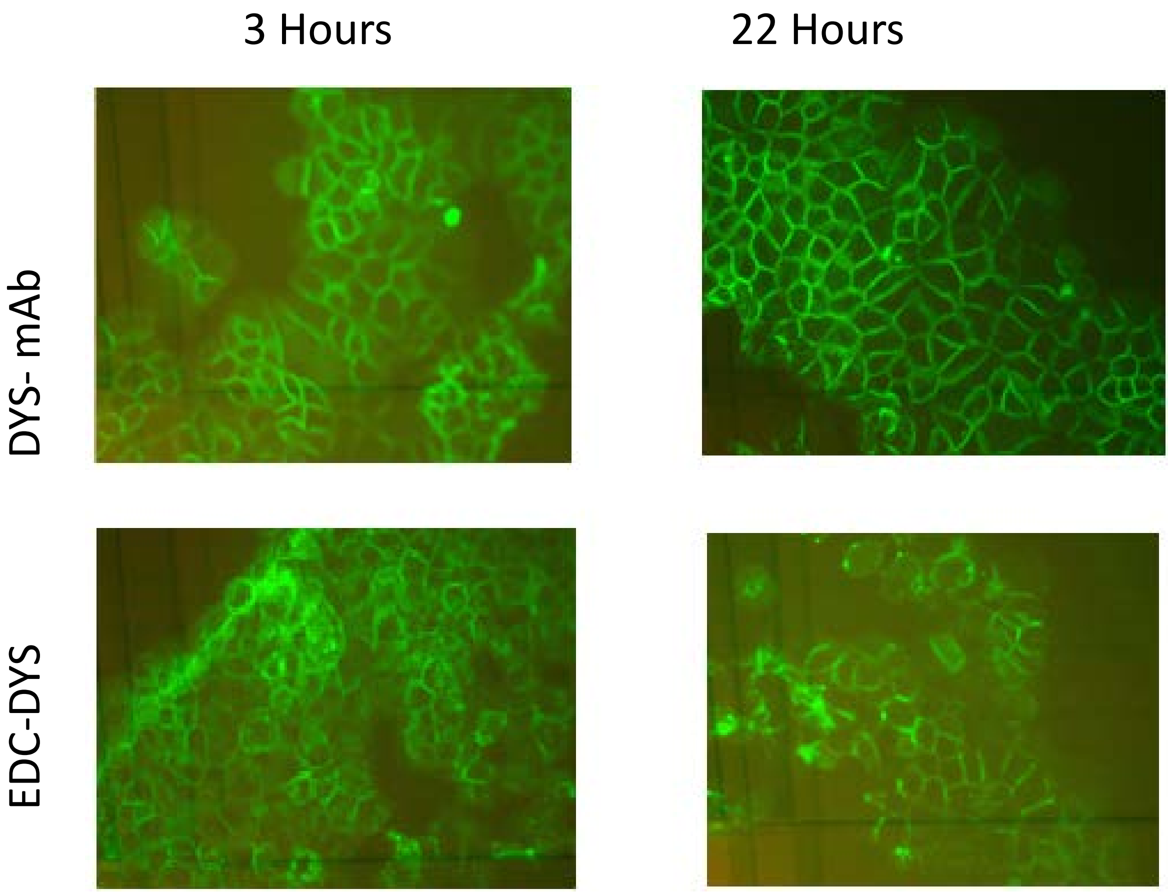

Supplement Figure 5A. Data showing EDC-DYS is poorly internalized. Cell images are shown post incubation of EDC-DYS and free anti-DYS antibody at time points indicated. In order to observe antibody localization, cells were fixed and permeablized and goat anti-mouse IgG Dylight® 488 conjugate was added. Fluorescent images show anti-DYS antibody and EDC-DYS displaying a pattern of surface staining (bright staining seen along boarders and junctions of the cells).

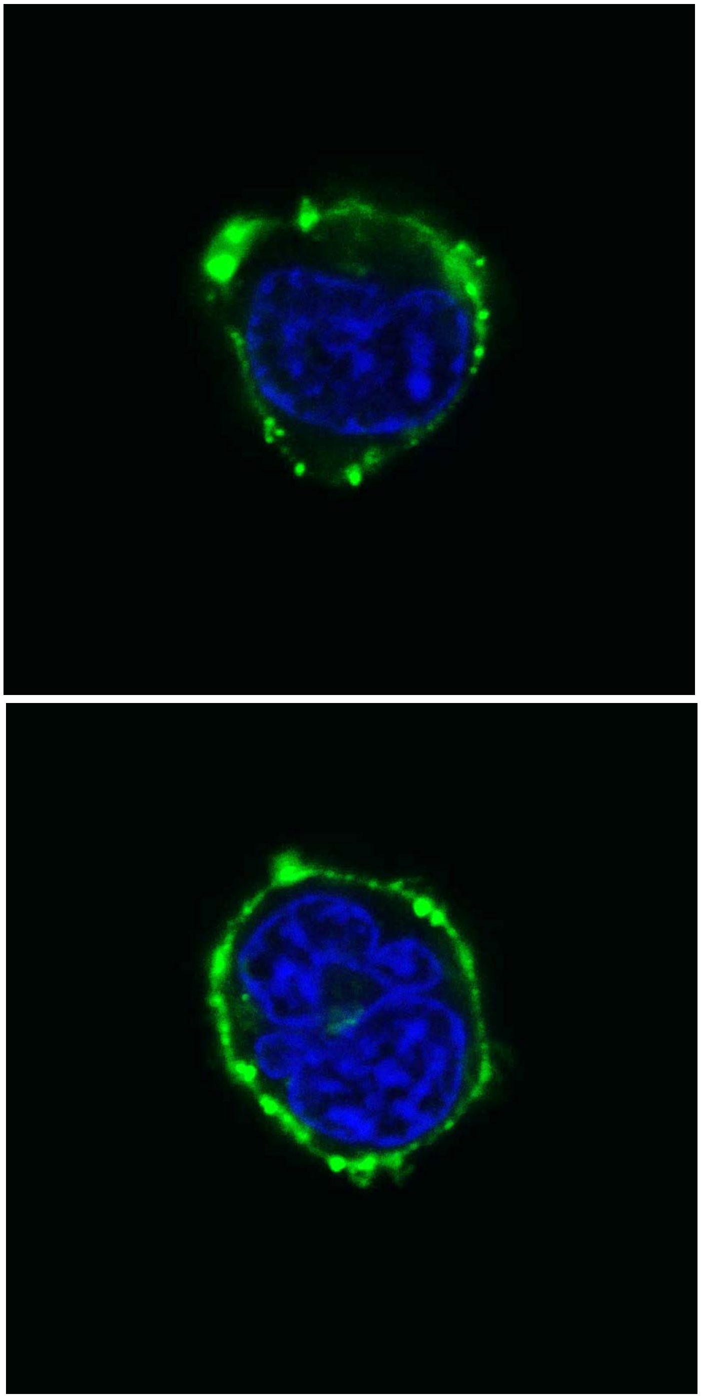

Supplement Figure 5B. Data showing EDC_CD38 is not internalized. Cell images are show EDC-CD38 localization (green) after 20 hours of incubation. In order to observe antibody localization, cells were incubated with EDC-38 and affinity-purified F(ab')2 fragment goat anti-mouse IgG (H+L) conjugated with Alexa Fluor 488. Fluorescent images show EDC-CD38 displaying a pattern of surface staining (bright green staining seen along boarders of the cells). DNA was visualized after permeabilizing the cells with 0.2 % Triton X-100 detergent (5 min at 4 °C) and staining with 4',6-diamidino-2-phenylindole (DAPI).

A.

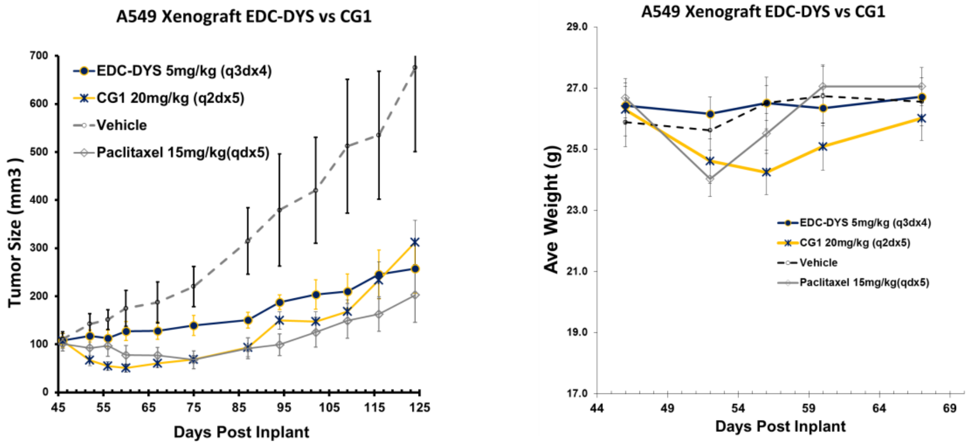

B.

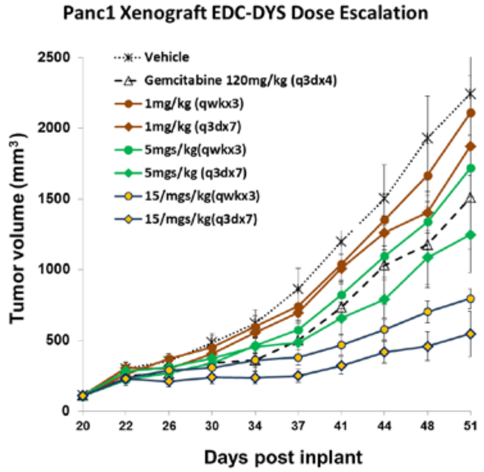

Supplemental Figures 6A and 6B. Xenograft study analysis graphs. Data plotted as group mean tumor volumes, with standard error of the mean (SEM) indicated with vertical bars. **A. (Left)** EDC-DYS comparison to an efficacious dose of CG1 and (Right) Group mean body weight during study. Athymic nude (Bal/cAnNHsd) mice (n = 7 per group) bearing s.c. A549 human tumors of an average size of $100mm^3$ were treated with EDC-DYS (5 mg/kg q3dx4, i.v.), CG1 (20 mg/kg q2dx5, i.v.), or paclitaxel (15 mg/kg qdx5, i.v.). Data are plotted as group mean tumor volumes, with SEM indicated. **B.** Athymic nude (Bal/cAnNHsd) mice (n = 10 per group) bearing s.c. PANC-1 human tumors of an average size of $100mm^3$ were treated with EDC-DYS at 1, 5, and 15 mg/kg administered q7dx2 or q3dx7 via i.v. injection. Data plotted as group mean tumor volumes, with SEM indicated. intraperitoneal injection of 30 mg/kg cyclophosphamide, 2.475 mg/kg doxorubicin, 0.375 mg/kg vincristine, and oral dosing of 0.15 mg/kg prednisone once a day for 5 days).

C.

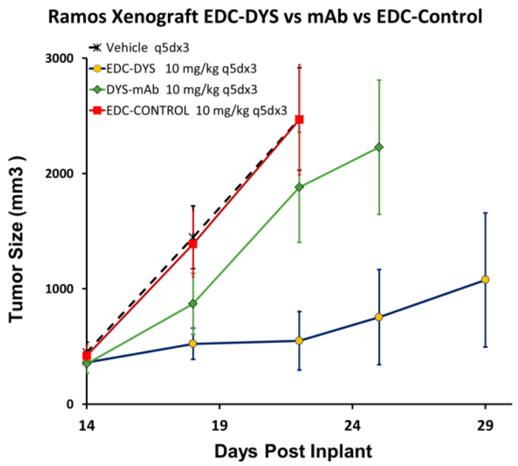

D.

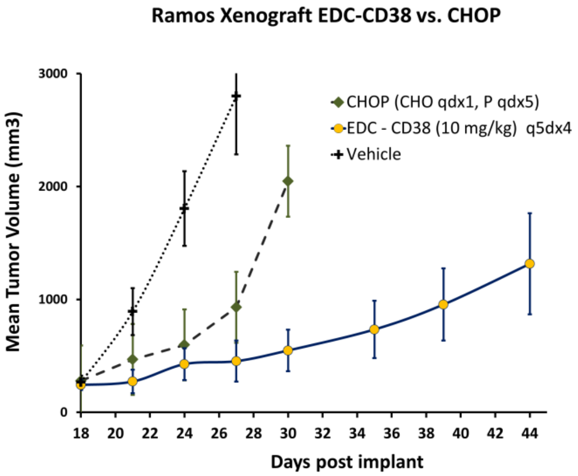

Supplemental Figures 6C and 6D. Xenograft study analysis graphs. Data plotted as group mean tumor volumes, with standard error of the mean (SEM) indicated with vertical bars. **C.** SCID Hairless Outbred (SHO) mice (n = 5 per group) bearing s.c. Ramos human tumors of an average size of 400mm^3 were treated with EDC-DYS, DYS-mAb, or EDC-Control administered q5dx3 via i.p. injection. Data plotted as group mean tumor volumes, with SEM indicated. **D.** SHO mice (n = 5 per group) bearing s.c. Ramos human tumors of an average size of 300mm^3 were treated with EDC-C38 at 10 mg/kg intraperitoneally q5dx4. CHOP (a single intraperitoneal injection of 30 mg/kg cyclophosphamide, 2.475 mg/kg doxorubicin, 0.375 mg/kg vincristine, and oral dosing of 0.15 mg/kg prednisone once a day for 5 days).

Supplemental Table

CELL LINE	A549
Tissue	NSCLC
DYS-PEG36-CG1 (144 Å)	0.40
DYS-PEG24-CG1 (105 Å)	1.20
DYS-PEG12-CG1 (63 Å)	42
DYS-PEG2-CG1 (27 Å)	>100
CG1	1.1
PEG2-CG1 (27 Å)	17
PEG12-CG1 (63 Å)	18.0
PEG24-CG1 (105 Å)	33.0
PEG36-CG1 (144 Å)	>200

Supplemental Table

CELL LINE	A549	PANC1	HT29	FaDu	LOX
Tissue	NSCLC	Pancreas	Colon	H&N	Melanoma
CD147-PEG36-CG1 (144 Å)	0.13	0.34	0.6	0.3	0.2
CD147-PEG24-CG1 (105 Å)	0.08	0.17	0.4	0.2	0.2
CD147-PEG12-CG1 (63 Å)	0.33	0.38	0.7	0.5	4.8
CD147-PEG2-CG1 (27 Å)	18	8	44	23	65
CG1	1.1	1.3	2.5	2.5	2.5
PEG2-CG1 (27 Å)	17	12	34	34	40
PEG12-CG1 (63 Å)	18.0	19	36.0	43.0	42.0
PEG24-CG1 (105 Å)	33.0	21.0	62.0	67.0	73.0
PEG36-CG1 (144 Å)	>200	>150	>300	>400	>500

Supplemental Tables 1A and 1B. Linker lengths optimization study. Activities of CG1 conjugates made with the 4 different linkers and either anti-DYS mAb (**A**) or anti-CD147 mAb (**B**) or no mAb (**A** and **B**). The top line shows the cell line designation used to derive the EC_{50} values with tissue type directly below. Below these are the experimentally derived EC_{50} value in picomoles per liter for that cell type employing the indicated conjugate, CG1 or linker-CG1. The > signs are used to show no activity was observed at the indicated highest level tested for that EDC. Linkers are indicated by PEGn where n=number of repeating polyethylene glycol units. Linker length is depicted in angstroms (Å) and was determined by adding all bond lengths from antibody thiol (-S-) to CG1 sugar amine (-N-).

	CELL LINE	A549	PANC1	Ramos	SUDHL4	SUDHL8	U937	RPMI-8226	LOX	A375	MRC-9	H520	H69	FaDu	HT29	HUAEC	HREpC	PBMC
	Type	NSCLC	Pancreas	Lymphoma	Lymphoma	Lymphoma	Lymphoma	Leukemia	Melanoma	Melanoma	Fibroblast	Lung	SCLC	H&N	Colon	Artery	Kidney	Blood
NKA Association	Cancer/Normal	C	C	C	C	C	C	C	C	C	C	C	C	C	C	N	N	N
	NKA	+	+	+	+	+	+	+	+	+	+	+	+	+	+	+	+	+
	CG1	0.7	1.1	1.1	1.0	1.7	2.1	2.0	2.5	1.5	1.1	2.0	1.0	3.1	2.9	2.0	2.0	2.0
Known POS	Dysadherin	+	+	+	+	+	+	+	+	+	+	–	–			+	+	+
	EDC-DYS	0.18	0.26	0.04	0.06	0.25	0.11	0.30	0.36	0.36	0.41	>50	>100			>50	>200	>200
Control NEG	Fluorescein	–	–	–	–	–	–	–	–	–	–	–	–	–	–	–	–	–
	EDC-CONTROL	>50	>50						>200	>200	>100	>100	>50	>200	>200			>200
Control NEG	Peptide	–	–	–	–	–	–	–	–	–	–	–	–	–	–	–	–	–
	EDC-CONTROL	>50	>50						>200	>200	>100	>100	>50	>200	>200	>200	>200	>200
Hypothesized POS	CD147	+	+	+	+		+		+	+	+	+	+	+	+	+	+	+
	EDC-CD147	0.20	0.15	0.03	0.02		0.03		0.20	0.23	0.17	0.06	0.08	0.18	0.41	>50	>200	>200
Hypothesized POS	CD56	–	–						–				++					
	EDC-CD56	>50	>50						>200				0.08					
Unknown POS	CD20	–	–	+	+	+/-	–	–										+
	EDC-CD20	>50	>50	0.10	0.05	>50	>50	>50										≥100
Unknown POS	CD38	+	–	+	+	+	+											+
	EDC-CD38	>50	>50	0.05	0.02	0.05	>50											≥100
Unknown NEG	HER2	+	+							–								
	EDC-HER2	>50	>50							>50								
Unknown NEG	Seprase (FAP)	–	–						+	–	+	–						
	EDC-FAP	>50	>50						>50	>50	>50	>50	>50					

Supplemental Table 2. mAb target presence and EDC EC_{50} values resulting from various treatments on cancerous and normal cell lines.

The top line shows the cell line designation with tissue type and state [Cancer (C) and Normal (N)] directly below. Below these are designations on whether the cell type expressed the EDC target (+/-) and the experimentally derived EC_{50} value in picomoles per liter for that cell type when either the indicated EDC or CG1 was added. The > signs are used to show no activity was observed at the indicated highest level tested for that EDC. The ≥ signs are used to show some activity was observed at the indicated highest level tested for that EDC but an EC_{50} could not be obtained.
