# Supplementary material for: Extracellular Antibody Drug Conjugates Exploiting the Proximity of Two Proteins
Source: Mol Ther. 2016 Jul 19;24(10):1760–70. doi: 10.1038/mt.2016.119 (PMC5112037; doi:10.1038/mt.2016.119)
Supplement: Supplementary Data [file mt2016119x2.pdf]

Supplementary data for:

# Extracellular Antibody Drug Conjugates Exploiting the Proximity of Two Proteins

David J. Marshall[1], Scott Harried[1], John Murphy[1], Chad Hall[1], Mohammed Saleh Shekhani[x], Christophe Pain[x], Conner Lyons[x], Jon S. Thorson[2], Antonella Chillemi[3], Fabio Malavasi[3], Homer L. Pearce[4], and James R. Prudent[1*]

[1] Centrose, 918 Deming Way, Madison, Wisconsin 53717, USA

[2] Center for Pharmaceutical Research and Innovation, University of Kentucky College of Pharmacy, 789 South Limestone Street, Lexington, KY 40536-0596

[3] Laboratory of Immunogenetics, Department of Medical Sciences, University of Torino, Torino, Italy.

E-mail: prudent@centrosepharma.com; Tel: (608) 836-0207

**Table of Contents**

1. Abbreviations
2. Materials and Methods
3. Synthesis of CG1 and PEG derivatives
4. Analytical data ($^1$H-NMR, $^{13}$C-NMR, MS and HPLC) CG1 and PEG derivatives and intermediates

## 1. Abreviations:

ACN: acetonitrile; BzCl: benzoyl chloride; calcd: calculated; DAR: drug antibody ratio; DBU: 1,8 diazabicyclo[5,4,0]undec-7-ene; DEA: diethanolamine; DPBS: Dulbecco's Phosphate-Buffered Saline; DTPA: diethylenetriamine pentaacetic acid; DMA: N,N-dimethylacetamide; ESI: electrospray ionization; FBS: Fetal Bovine Serum; HPLC: high performance liquid chromatography; MALDI: matrix assisted laser desorption ionization; NHS: N-hydroxysuccinimide ester; PEG: poly(ethylene glycol); PNPP: p-Nitrophenyl phosphate; $R_t$: retention time; RT: room temperature; TCEP: Tri(2-carboxyethyl) phosphine hydro-chloride; TFA: trifluoroacetic acid; $Tf_2O$: trifluoromethanesulfonic anhydride; THF: tetrahydrofurane; $Zn(OTf)_2$: zinc trifluoromethanesulfonate

## 2. Materials and Methods

**Reagents and antibodies:** Reagents and solvents were used *sans* further purification unless otherwise specified- Polyethylene glycol-derivatives (Quanta Biodesign), Proscillaridin-A (Molekula Ltd.), *N*-phthalimido-tetracetyl-glucosamine (Carbosynth), *p*-nitrophenyl phosphate (Pierce Biotechnology), and tri(2-carboxyethyl)phosphine hydrochloride (Hampton Research).

Antibodies used: Mouse IgG1 α-dysadherin clone NCC-M53 (National Cancer Center, Tokyo), mouse IgG1 α-EMMPRIN clone 8D12 (eBiosciences), mouse IgG1 α-NCAM clone HCD56 (Biolegend), mouse IgG1 α-CD29 clone MEM-101A (Biolegend), mouse IgG1 α-CD47 clone CC2C6 (Biolegend), mouse/human chimeric IgG1 α-CD20 Rituximab (Genentech), IgG1 α-CD38 clone SUN4B7 (A. Malavasi). Human IgG Protein-A purified from normal serum (Innovative Research) was used make EDC-Control. Antibodies used for western blotting were; ab175213, ab181602, ab76020, ab2873, and ab137055 (Abcam).

**Synthesis of CG1 antibody conjugates:** **A**ntibody conjugates were prepared by reduction-alkylation of antibody inter-chain disulfides. Briefly, between 1-10 mg/mL of antibody in DPBS was reduced in the presence of 1 mM DTPA and 8 molar equivalents of TCEP at 37°C for 2 hours. These mixtures were then cooled in an ice bath, at which time 9.6 molar equivalents of a maleimide activated CG PEG compound (CG1 PEG2, CG1 PEG12, CG1 PEG24 or CG1 PEG36) was added and allowed to react for 30 min on ice. Residual maleimide groups were then quenched by the addition of a 1.5 equivalent excess of L-cysteine (based upon maleimide), which was allowed to react for 30 minutes at RT. Resulting antibody conjugates were separated from Cys-capped reagents by forced dialysis using Amicon Ultra 30,000 MWCO centrifugal filters (Millipore) and DPBS buffer exchange (3X). Conjugates were stored ($4^oC$) in DPBS at concentrations ranging from 1-10 mg/mL.

**DAR calculation:**

Drug antibody ratios were determined by measuring absorbance of conjugates, antibodies (Ab) and free drug (drug) at both 280 nm and 299 nm. Next, the following constants were determined; [Constant Ab] = A299Ab / A280Ab; [Constant Drug] = A299drug / A280drug. Next, absorbance of antibody drug conjugate was measured [A280 and A299]. Drug loading = drug concentration / antibody concentration.

Using the following, drug loading was determined.

A280Ab = A280 - ( A299 – [Constant Ab] x A280) / ( [Constant drug] – [Constant Ab])

A299drug = A299-[Constant Ab] x A280Ab

Antibody concentrations = A280Ab / 204,000 M-1cm-1 (varies depending on antibody)

Drug concentration = A299drug / 5623 M-1cm-1

A299drug = drug component

A280Ab = antibody component

**Cell Lines**: A375, A549, BF01, FaDu, H520, H69, HT29, MRC-9, PANC-1, Ramos, RPMI-8226, SU-DHL-4, SU-DHL-8, U937 (ATCC), and LOX-IMVI (NCI) cells were grown in ATCC/NCI recommended media supplemented with 50 ug/mL gentamycin (GIBCO). HUAEC and HREpC cell lines were maintained in Endothelial Cell Growth Medium 2 and Renal Epithelial Cell Growth Medium 2 (PromoCell GmbH). All cells were grown at 37 °C in a humidified atmosphere with 5% $CO_2$.

**Peripheral Blood Mononuclear Cell (PBMC) Testing**: PBMC from healthy donors were immediately plated (2 x $10^5$/ml) in RPMI 10% FCS and treated with EDC for 20 hours. Cells were then collected and stained with FITC-conjugated annexin-V (BioLegend) and propidium iodide (Invitrogen) for 15 minutes at room temperature. Apoptosis was measured using a FACSCanto flow cytometer (Becton-Dickinson) and evaluated with the FlowJo software.

**Fluorescent Antibody Staining.** Cells were stained with 1 μg/ml of primary antibodies and 5 μg/ml goat anti-mouse IgG, DyLight 488 (Pierce Biotechnology) in DPBS with 1.5% FBS for 30 min, washed twice, and imaged using a Nikon Diaphot-TMD inverted fluorescence microscope.
PBMC from healthy donors were plated (2 x $10^5$/ml) in RPMI 10% FCS and treated with EDC for 20 hours, collected, and stained with FITC-conjugated Annexin-V (BioLegend) and propidium iodide (Invitrogen) for 15 minutes at room temperature. Apoptosis was measured using a FACSCanto flow cytometer (Becton-Dickinson) and evaluated with the FlowJo software. Early apopstosis was defined as Annexin V-positive, PI-negative and late apoptosis as Annexin V-positive, PI-positive.

**Cell proliferation/Cytotoxicity assays:** For Cell Titer-Glo assays, cells were treated 24 hrs after plating and Cell Titer-Glo Luminescent Cell Viability Assay (Promega) was performed 72 hrs later. Luminescence measurements were performed using a Wallac Victor$^2$ Model 1420-041 assay plate reader

(Perkin Elmer, Gaithersburg, MD). $EC_{50}$ values for each test agent were determined with GraphPad Prism 5 software (GraphPad).

**Cell morphology:** A549 cells (~ 1,500) were plated in 96 well plates in full media containing 125nM Sytox Green, treated as indicated with EDCs/steroidal glycosides after 24 hours, and imaged by phase-contrast with an Incucyte Zoom (Essen Biosciences) using a 20X objective. Images were adjusted using Incucyte Zoom software (Essen Biosciences).

**siRNA combination studies:** A549 cells (~ 370) were reverse transfected in 384 well plates with 0.2, 0.12 or 0.04 picomoles of total Dharmacon Smartpool siRNAs and 0.06 μL of RNAiMax transfection reagent per well in full media containing 125nM Sytox Green if needed for analsysis. After 24 h EDCs/steroidal glycosides were added and cells were imaged over a period of 5 days using the Incucyte Zoom (Essen Biosciences) or after 72 hours Cell Titer-Glo Luminescent Cell Viability Assay was performed. Percent confluence at 5 days was determined using Incucyte Zoom Software (Essen Biosciences). At the end of five days, Triton X100 (1% final conc.) was added to each well and plates were rescanned to determine the total number of cells per well. Percent dead was calculated by dividing the number of Sytox Green positive cells before Triton X100 addition the total number per well. Percent confluence and percent dead data were analyzed and EC50 curves were plotted using GraphPad Prism 5 software (GraphPad).

**EDC internalization studies:**

Experiments were performed following Polson et al. (Antibody-Drug Conjugates for the Treatment of Non–Hodgkin's Lymphoma: Target and Linker-Drug Selection. Cancer Res 2009; 69: (6): 2358-64). Briefly, H460 cells (large cell lung cancer) were seeded into 96-well, clear bottom, tissue culture treated plates at a density of 10,000 cells / well in 100 uL media (RPMI 1640 with 10% fetal bovine serum and

50 ug/mL Gentamicin) / well. Cells were incubated at 37C and 7% $CO_2$ for 24 hrs. NCC-M53 and EDC-DYS were diluted in media, with and without protease inhibitors (10 ug/mL leupeptin, and 5 uM pepstatin), to a final concentration of 2 ug/mL added to wells (100 uL/well) at time points -21 or -3 hrs. At time 0 hrs, media was removed by aspiration and the wells were washed 1X with DPBS + 1.5% FBS. Cells were then fixed with 4% formaldehyde in PBS for 20 min at room temp and permeabilized with 0.1% TritonX-100 in PBS for 15 min at room temp. For controls, cells that did not receive antibody during culture were stained with M53 or M53-PEG24-CEN09-106 after fixation and permeabilization. Cells were then washed twice with DPBS + 1.5% FBS. Goat anti-mouse IgG Dylight® 488 conjugate (Thermo) secondary was diluted to 5 ug/mL in DPBS + 1.5% FBS and added to all wells at 100 uL/well and incubated for 30 min at room temp. Cells were again washed, and then viewed with Nikon Diaphot TMD inverted trinocular fluorescence phase contrast microscope with Ph2 20X DL objective. Pictures were captured using an Olympus E-450 Digital SLR camera, and the brightness of fluorescence images were adjusted using GIMP 2.6 software.

**Xenograft Studies:**

**General:** EDCs were diluted in sterile saline for injection and dosed as indicated. Paclitaxel (LC laboratories) was dissolved as a 15 mg/ml in ethanol/Cremophor EL (1:1) stock solution, and stored at -80°C. This stock solution was diluted 1.5 mg/mL in saline just before injection at 10 uL per gram of mouse body weight. Gemcitabine (LC laboratories) in saline stored at room temp and dosed at 120 mg/kg. CHOP consists of a single intraperitoneal injection of 30 mg/kg cyclophosphamide (Sigma-Aldrich), 2.475 mg/kg doxorubicin (LC Laboratories), and 0.375 mg/kg vincristine (LC Laboratories), in addition to oral dosing of 0.15 mg/kg prednisone (Sigma-Aldrich) once a day for 5 days.

Tumor sizes were calculated using the formula: Tumor Volume ($mm^3$) = (w2 x l)/2 where w = width and l = length in mm of a given tumor.

**Non-small-cell-lung cancer xenograft:** A549 tumor line was maintained by serial subcutaneous transplantation in athymic nude mice. A549 tumor fragments (~ 1 to 2 $mm^3$ each) were implanted subcutaneously via trocar into the left flank of female HRLN nu/nu mice of 8-12 weeks old (Harlan Laboratories). Animals were randomized (6 mice per group; 4 groups) and treatment initiated when tumors reached an average volume of 100 $mm^3$. Tumor-bearing mice were dosed as shown in Figure 4. Every four to seven days tumor volume was assessed for each group using calibrated vernier calipers and results were plotted against time from day of first dose.

**Pancreatic Cancer xenograft:** PANC-1 tumor cells were maintained by serial subcutaneous transplantation in athymic nude mice, and PANC-1 tumor fragments (1 $mm^3$) were implanted subcutaneously into the right flank of female HRLN nu/nu mice of 8-12 weeks old (Harlan Laboratories). Mice bearing tumors of 90 – 130 $mm^3$ were randomized by tumor size and segregated into 9 groups (n = 10 mice / group). Group mean tumor volumes ranged from 106 –112 $mm^3$ and individual tumor volumes from 63 $mm^3$ – 172 $mm^3$. Tumor-bearing mice were dosed as shown in Figure 3. Twice per week tumor volume was assessed for each group using calibrated vernier calipers and results were plotted against time from day of first dose.

**B-cell lymphoma xenograft:** Ramos and SU-DHL-4 cells were washed, suspended in Dulbecco's PBS (Hyclone), and inoculated subcutaneously ($1 \times 10^7$ cells suspended in 0.2 mL per mouse) into the flanks of female SHO mice 28 to 35 days old (Ramos) and CB17SCID mice 36 to 42 days old (SU-DHL-4) (Charles River Laboratories). When mean tumor size reached ~ 250 $mm^3$ mice were divided into groups (5 mice/group- Ramos; 6 mice/group- SU-DHL-4) with the same mean tumor size and dosed by intraperitoneal injection.

**3. Synthesis of CG1 and PEG derivatives:**

**General Analytical Chemistry:** $^{1}$H-NMR and $^{13}$C-NMR analyses were performed using a Varian Mercury Plus 300 MHz NMR spectrometer (Dept. of Chemistry, UW-Madison). ESI-MS spectra were obtained using an AB Sciex QTRAP, and ESI-HRMS spectra were generated with an Agilent LC/MSD TOF instrument. All samples (in methanol or 1:1 acetonitrile-water) were injected into the electrospray source at a rate of 30 µL/min. MALDI-MS spectra were obtained with an ABI Sciex 4800 TOF/TOF mass spectrometer using α-cyano-4-hydroxycinnamic acid (CHCA) as an ionization matrix (Biotechnology Center of the UW-Madison). HPLC analyses were performed on a Waters modular system consisting of a Delta 600 fluid handler, a 600 controller, a 2777 auto sampler, and a 2996 PDA detector. For analytical HPLC, a Gemini C18 5 µm, 110 Å, 250 mm X 4.6 mm column was used with a 100 µL injection loop. Semi-preparative HPLC was performed on the same system via manual injection with a 500 µL injection loop and Gemini C18 5 µm, 110, 250 mm X 10.0 mm column. TLC analysis was performed using EMD glass-backed TLC plates pre-coated with a 0.25 mm layer of silica gel 60 $F_{254}$ (EMD Chemicals, Gibbstown, NJ). TLC protocols were developed based on one or more of the following detection techniques: (i) UV at 254 nm, (ii) iodine vapors, and (iii) a solution of 2.5 g phosphomolybdic acid, 1.0 g ceric sulfate, 6.0 mL sulfuric acid, and 94 mL water, followed by heating. Flash chromatography was performed with a Biotage SP4 flash chromatography instrument (Charlottesville, VA) using Biotage cartridges. Absorbance values for the antibodies and **CG1** were determined on a Beckman DU530 UV/VIS spectrophotometer.

## Overall Scheme of Synthesis

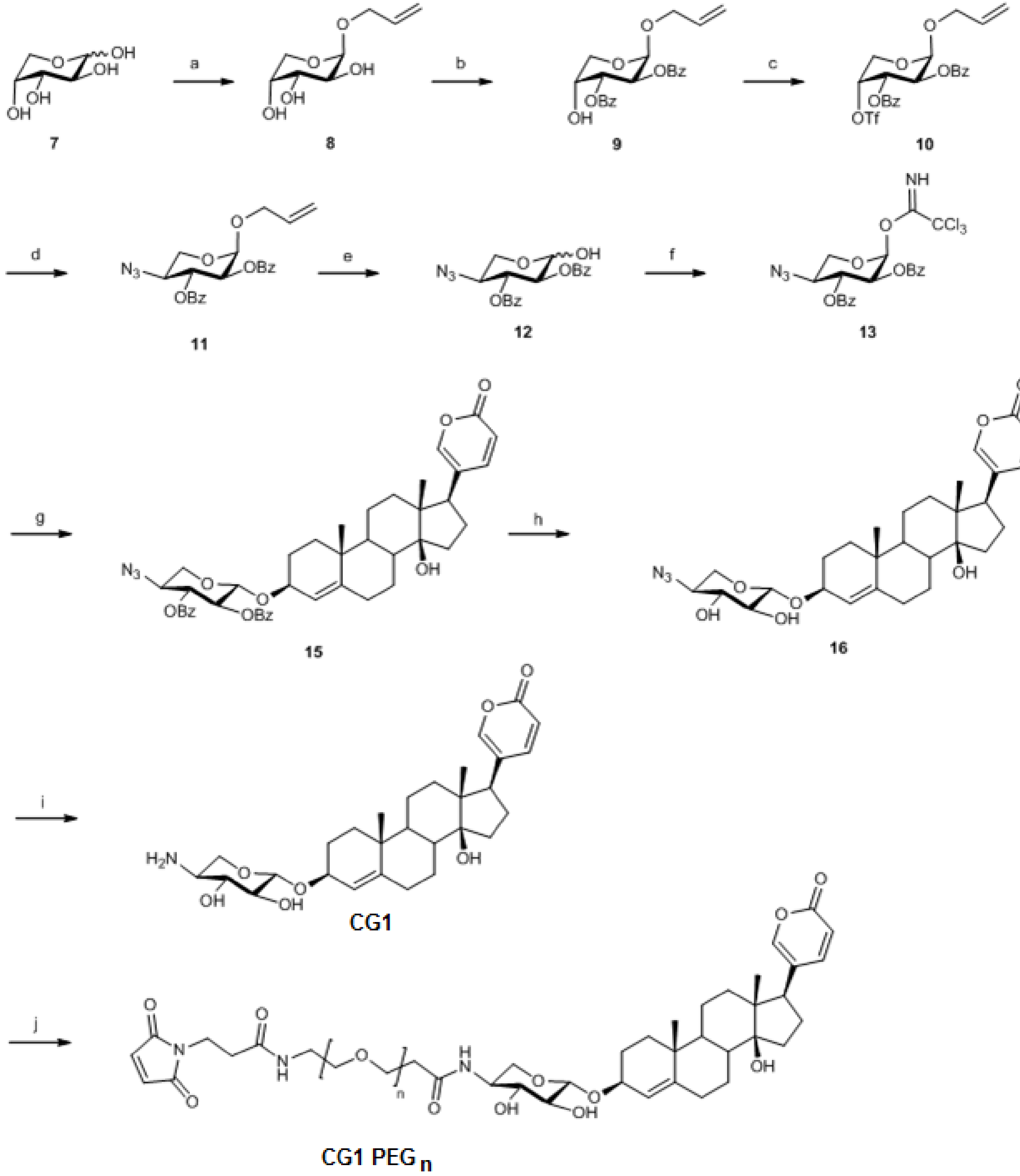


Conditions: (a) allyl alcohol, $Na_2SO_4$, conc $H_2SO_4$, 85 °C, 12 h, 60%. (b) BzCl, pyridine, RT, 18 h, 29%. (c) $Tf_2O$, pyridine, $CH_2Cl_2$, 0 °C, 15 min. (d) $NaN_3$, DMA, RT, overnight, 84% (2 steps). (e) $PdCl_2$, $CH_2Cl_2$, MeOH, RT, overnight, 78%. (f) $CCl_3CN$, DBU, $CH_2Cl_2$, 0 °C, 1 h, 92%. (g) Scillarenin (**14)**, $Zn(OTf)_2$, 4Å MS, $CH_2Cl_2$, 0 °C, 2 h, 80%. (h) NaOMe, MeOH, $CH_2Cl_2$, RT, 1.5 h, 23%, (i) $PPh_3$, THF, $H_2O$, 45 °C, 2.75 h, 81%, (j) Maleimide-$PEG_n$-NHS ester, $Et_3N$, DMA, RT, 1 h, **CG1PEG12** 41%, **CG1PEG24** 65%.

**1-*O*-allyl-α-D-arabinopyranoside (8):** D-Arabinose **7** (100 g, 666 mmol) and $Na_2SO_4$ (100 g, 703 mmol) were suspended in allyl alcohol (1L, 14.65 mol) at RT. To this suspension was added concentrated $H_2SO_4$ (10.4 mL, 187 mmol). The mixture was stirred overnight at 85 °C. The solids were removed by filtration and washed with allyl alcohol (2 x 300 mL). The combined filtrate was concentrated *in vacuo*. The crude material was purified by flash chromatography (silica gel, $CH_2Cl_2$ to $CH_2Cl_2$-MeOH 80:20) to give **8** as an off-white solid (75 g, 60%).

**1-*O*-allyl-2,3-di-*O*-benzoyl-α-D-arabinopyranoside (9):** To a solution of **8** (75 g, 394 mmol) in pyridine (800 mL) at 0 °C was added benzoyl chloride (101 mL, 867 mmol) over 2 hours. The reaction mixture was stirred at RT for 16 hours. The solvent was removed *in vacuo*. The crude material was purified by flash chromatography (silica gel, hexanes-EtOAc 8:2 to 6:4) to give **9** as a thick oil (45 g, 29 %). $^1$H-NMR (300 MHz, $CDCl_3$) $\delta$ 3.82 (dd, *J* = 2.0, 12.6 Hz, 1H, H-5), 4.01-4.10 (m, 2H, H-5), 4.22-4.29 (m, 2H), 4.37 (m, 1H, H-4), 5.13-5.34 (m, 1H), 5.29 (d, *J* = 3.4 Hz, 1H, H-1α), 5.67 (dd, *J* = 3.4, 10.6 Hz, 1H, H-2), 5.75 (dd, *J* = 3.1, 10.6 Hz, 1H, H-3), 5.79-5.92 (m, 1H), 7.35-7.42 (m, 4H), 7.47-7.56 (m, 2H), 7.98-8.01 (m, 4H).

**1-*O*-allyl-2,3-di-*O*-benzoyl-4-*O*-trifluromethanesulfonyl-α-D-arabinopyranoside (10):** To a solution of **9** (20.0 g, 50 mmol) in dry $CH_2Cl_2$ (100 mL) and pyridine (16.2 mL, 200 mmol) at 0 °C was added triflic anhydride (10.6 mL, 62.7 mmol). The reaction mixture was stirred at 0 °C for 15 min. $CH_2Cl_2$ (500 mL) was added to the solution and the organic layer was washed with cold 1N HCl (200 mL), saturated $NaHCO_3$ (200 mL) and brine (200 mL). Organics were then dried ($Na_2SO_4$), and concentrated *in vacuo* to give crude **10** as a yellow oil. The crude material was carried on without further purification.

**1-*O*-allyl-2,3-di-*O*-benzoyl-4-deoxy-4-azido-α-L-xylopyranoside (11):** Crude **10** (26.6 g, 50.1 mmol) was dissolved in *N,N*-dimethylacetamide (100 mL) and sodium azide (6.37 g, 98 mmol) was added to the solution. The mixture was stirred overnight at RT. The solvent was removed *in vacuo*, and the residue was dissolved in $CH_2Cl_2$ (750 mL). The organic layer was washed with water (2 x 200 mL) and brine (200 mL), dried ($Na_2SO_4$) and concentrated *in vacuo*. The crude material was purified by flash chromatography (silica gel, hexanes:EtOAc 8:2 to 6:4) to give **11** as a thick colorless oil (17.5 g, 84 %). $^1$H-NMR (300 MHz, $CDCl_3$) $\delta$ 3.73-3.93 (m, 3H, H-4, H-5, H-5), 3.98-4.05 (m, 1H), 4.20-4.27 (m, 1H), 5.12-5.17 (m, 1H), 5.17 (dd, *J* = 3.6, 10.0 Hz, 1H, H-2), 5.24 (d, *J* = 3.6 Hz, 1H, H-1), 5.26-5.32 (m, 1H), 5.76-5.89 (m, 1H), 5.91 (dd, *J* = 8.9, 10.0 Hz, 1H, H-3), 7.34-7.41 (m, 4H), 7.48-7.54 (m, 2H), 7.96-8.01 (m, 4H).

**2,3-Di-*O*-benzoyl-4-deoxy-4-azido-L-xylopyranoside (12):** To a solution of **11** (15.7 g, 37.1 mmol) in $CH_2Cl_2$-MeOH (100 mL, 90:10) under argon was added $PdCl_2$ (0.66 g, 3.7 mmol). The reaction mixture was stirred overnight at RT, filtered through a pad of celite and concentrated. The crude material was purified by flash chromatography (silica gel, hexanes-EtOAc 8:2 to 5:5) to give **12** as a thick colorless oil and as 2:1 mixture of α/β (11.1 g, 78 %). $^1$H-NMR (300 MHz, $CDCl_3$) $\delta$ 3.46 (dd, *J* = 10.6, 11.9 Hz, 0.5H, H-5*β*), 3.82-3.92 (m , 3.5H, H-4*α*, H-5*α*, H-5*α*, H-4*β*), 4.19 (dd, *J* = 5.4, 11.9 Hz, 0.5H, H-5*β*), 4.86 (d, *J* = 7.7 Hz, 0.5H, H-1*β*), 5.17 (dd, *J* = 3.5, 10.0 Hz, 1H, H-2*α*), 5.19 (dd, *J* = 7.7, 9.5 Hz, 0.5H, H-2*β*), 5.60 (dd, *J* = 9.5 Hz, 0.5H, H-3*β*), 5.62 (d, *J* = 3.3 Hz, 1H, H-1*α*), 5.93 (dd, *J* = 9.1, 9.8 Hz, 1H, H-3*α*), 7.32-7.41 (m, 6H), 7.46-7.55 (m, 3H), 7.94-8.02 (m, 6H); $^{13}$C-NMR (75 MHz, $CDCl_3$) $\delta$ 59.7, 59.7, 59.9, 64.0, 70.8, 72.4, 73.1, 74.1, 90.8 (C-1*α*), 96.4 (C-1*β*), 128.6, 128.6, 128.6, 128.8, 128.9, 129.0, 129.2, 129.9, 129.9, 130.0, 130.1, 133.5, 133.6, 133.7, 133.8, 165.7, 165.8, 166.1, 166.9.

**2,3-di-*O*-benzoyl-4-deoxy-4-azido-α-L-xylopyranoside 1-trichloroacetimidate (13):** To a solution of **12** (11.1 g, 28.9 mmol) in dry $CH_2Cl_2$ (180 mL) under argon was added trichloroacetonitrile (29 mL,

289.5 mmol) at 0 °C, followed by DBU (0.8 mL, 5.2 mmol). The reaction mixture was stirred at 0 °C for 1 hour. The solvent was removed *in vacuo*. The crude material was purified by flash chromatography (silica gel, hexanes-EtOAc 9:1 to 8:2) to give **13** as a yellow oil (14.04 g, 92%). $^{1}$H-NMR (300 MHz, $CDCl_3$) $\delta$ 3.92-4.11 (m, 3H, H-4, H-5, H-5), 5.43 (dd, $J$ = 3.5, 10.1 Hz, 1H, H-2), 5.98 (dd, $J$ = 9.7 Hz, 1H, H-3), 6.66 (d, $J$ = 3.5 Hz, 1H, H-1), 7.33-7.56 (m, 6H), 7.93-8.01 (m, 4H), 8.61 (s, 1H, NH); $^{13}$C-NMR (75 MHz, $CDCl_3$) $\delta$ 59.4, 62.2, 70.8, 70.8, 90.8, 93.6, 128.6, 128.6, 128.6, 129.0, 129.9, 130.0, 133.7, 133.7, 160.8, 165.6.

**Scillarenin (14):** To a solution of proscillaridin A (3 g, 5.6 mmol) in ethanol (60 ml) at 40 °C was added sodium acetate buffer (170 mL, 0.02 M, pH 4.0), followed by naringinase (1.03 g, Sigma-Aldrich, Cat# N1385). The reaction mixture was stirred overnight, diluted with ethanol (200 mL), filtered through a pad of Celite and the filtrate was concentrated *in vacuo*. The residue was purified by flash chromatography (silica gel, $CH_2Cl_2$-MeOH 98:2 to 90:10) to give **14** as an off-white solid (1.9 g, 90 %). $^{1}$H-NMR (300 MHz, $CD_3OD$) $\delta$ 0.74 (s, 3H), 0.99-1.25 (m, 5H), 1.25-1.55 (m, 6H), 1.60-1.79 (m, 4H), 1.85-2.24 (m, 6H), 2.53 (dd, $J$ = 6.5, 9.5 Hz, 1H), 4.06-4.12 (m, 1H), 5,28 (d, $J$ = 1.1 Hz, 1H), 6.27 (dd, $J$ = 0.8, 9.7 Hz, 1H), 7.39 (dd, $J$ = 0.9, 2.4 Hz, 1H), 7.98 (dd, $J$ = 2.6, 9.7 Hz, 1H).

**Scillarenin-2’,3’-di-*O*-benzoyl-4’-deoxy-4’-azido-β-L-xylopyranoside (15):** A solution of **13** (14.04 g, 26.6 mmol) in dry $CH_2Cl_2$ (100 mL) was added to a suspension of activated 4 Å molecular sieves (2 g) in dry $CH_2Cl_2$ (100 mL) under argon at 0 $^{o}$C. Scillarenin (**14,** 10.2 g, 26.6 mmol) was then added to the mixture. After 10 min of stirring at 0 °C, $Zn(OTf)_2$ (0.96 g, 2.66 mmol) was added. The reaction mixture was stirred at 0 °C for 2 hours. The reaction was quenched with $Et_3N$ (3.7 mL, 26.6 mmol). The mixture was filtered and the solvent was removed *in vacuo*. The crude material was purified by flash chromatography (silica gel, hexanes-EtOAc 7:3 to 1:1) to give **15** as a white powder (15.93 g, 80%). $^{1}$H-NMR (300 MHz, $CDCl_3$) $\delta$ 0.70 (s, 3H), 0.92 (s, 3H), 0.96-1.18 (m, 4H), 1.35-1.66 (m, 7H), 1.71-2.01 (m, 4H), 2.05-2.19 (m, 4 H), 2.41-2.46 (m, 1H), 3.49 (dd, $J$ = 11.9, 9.5 Hz, 1H, H-5’), 3.86 (ddd, $J$ = 4.9, 9.1, 9.2 Hz, 1H, H-4’), 4.17-4.25 (m, 2H, H-3, H-5’), 4.80 (d, $J$ = 6.8 Hz, 1H, H-1’), 5.28 (dd, $J$ = 6.8, 8.6 Hz, 1H, H-2’), 5.35 (s,1H), 5.51 (dd, $J$ = 8.7 Hz, 1H, H-3’), 6.24 (dd, $J$ = 0.6, 9.7 Hz, 1H), 7.21-7.22 (m, 1H), 7.35-7.41 (m, 4H), 7.49-7.55 (m, 2H), 7.82 (dd, $J$ = 2.6, 9.7 Hz, 1H), 7,94-7.99 (m, 4H); $^{13}$C-NMR (75 MHz, $CDCl_3$) $\delta$ 16.6, 19.0, 21.3, 25.7, 28.7, 28.8, 32.3, 32.8, 35.1, 37.5, 40.7, 42.8, 48.3, 50.1, 51.2, 59.1, 63.0, 71.5, 72.9, 76.0, 85.1, 99.9, 115.4, 121.6, 122.7, 128.5, 128.5, 129.1, 129.5, 129.8, 130.0, 133.3, 133.6, 146.8, 147.5, 148.7, 162.4, 165.2, 165.7.

**Scillarenin-4’-deoxy-4’-azido-β-L-xylopyranoside (16):** A solution of **15** (15.66 g, 20.9 mmol) in MeOH-$CH_2Cl_2$ (590 mL, 75:25) was cooled to 0 °C. NaOMe (25% in MeOH, 2.1 mL, 10.3 mmol) was subsequently added and the reaction mixture stirred at RT for 1.5 hours. The reaction was quenched with acetic acid (0.6 mL, 10.3 mmol) and the crude material was purified by flash chromatography (silica gel, $CH_2Cl_2$-MeOH 98:2 to 90:10) to give **16** as a white powder (2.6 g, 23%). $^{1}$H-NMR (300 MHz, $CD_3OD$) $\delta$ 0.61 (s, 3H), 0.87-1.03 (m, 5H), 1.10-1.69 (m, 10H), 1.78-2.10 (m, 6H), 2.36 (dd, $J$ = 6.4, 9.5 Hz, 1H), 3.02-3.09 (m, 1H, H-5’), 3.15-3.21 (m, 1H, H-2’), 3.35-3.39 (m, 2H, H-3’, H-4’), 3.84 (dd, $J$ = 4.5, 11.7 Hz, 1H, H-5’), 4.09-4.14 (m, 1H), 4.21 (d, $J$ = 7.5 Hz, 1H, H-1’), 5.27 (s, 1H), 6.16 (d, $J$ = 9.7 Hz, 1H), 7.16 (d, $J$ = 1.9 Hz, 1H), 7.8 (dd, $J$ = 2.5, 9.7 Hz, 1H); $^{13}$C-NMR (75 MHz, $CDCl_3$) $\delta$ 16.5, 18.7, 21.2, 25.5, 28.5, 28.7, 32.2, 32.3, 35.2, 37.4, 40.6, 42.3, 48.3, 50.1, 51.0, 61.1, 63.8, 73.7, 75.4, 75.5, 84.6, 102.0, 114.9, 121.6, 123.3, 147.5, 147.6, 148.6, 163.3.

**Scillarenin-4’-deoxy-4’-amino-β-L-xylopyranoside (CG1):** To a solution of **16** (2.6 g, 4.8 mmol) in THF-$H_2O$ (50 mL, 92:8) was added $PPh_3$ (2.5 g, 9.6 mmol). The reaction mixture was stirred at 45 °C for 2.75 hours. The mixture was then added dropwise to $Et_2O$ at 0 °C. The resulting precipitate was filtered and washed with $Et_2O$ and dried in air to give **1** as a slightly yellow powder (2.0 g, 81%). $R_t$ 12.70 min (Gemini C18, 5 µm, 4.6 mm x 250 mm, 10% to 95% ACN, 0.1% TFA, over 18 min, 1 mL.min$^{-1}$, 300 nm); $^{1}$H-NMR (300 MHz, $(CD_3)_2SO$) $\delta$ 0.63 (s, 3H), 0.90-0.99 (m, 5H), 1.12-1.30 (m,

2H), 1.35-1.71 (m, 8H), 1.79-1.91 (m, 2H), 2.02-2.10 (m, 4H), 2.43-2.47 (m, 1H), 2.52-2.57 (m, 1H, H-4'), 2.87-2.99 (m, 3H, H-2', H-3', H-5'), 3.64 (dd, $J$ = 4.9, 11.3 Hz, 1H, H-5'), 4.06-4.11 (m, 1H), 4.18 (d, $J$ = 7.0 Hz, 1H, H-1'), 4.25 (s, 1H), 5.27 (s, 1H), 6.29 (d, $J$ = 9.7, 0.6 Hz, 1H), 7.52 (d, $J$ = 1.6 Hz, 1H), 7.92 (dd, $J$ = 2.4, 9.9Hz, 1H); $^{13}$C-NMR (75 MHz, $(CD_3)_2SO$) $\delta$ 16.6, 18.5, 20.9, 25.4, 28.4, 28.4, 31.8, 32.0, 34.9, 37.0, 41.5, 47.9, 49.6, 49.9, 53.1, 66.6, 73.5, 73.6, 77.3, 83.1, 102.4, 114.2, 122.5, 122.6, 145.8, 147.3, 149.2, 161.3. ESI-MS (m/z): calcd for $C_{29}H_{41}NO_7$ $[M+H]^+$ 516.3, found 516.1.

**Compound CG1 PEG12:** To a solution of **1** (36 mg, 0.069 mmol) and maleimide-PEG$_{12}$-NHS ester (50 mg, 0.058 mmol) in *N,N*-dimethylacetamide (1 mL) was added $Et_3N$ (0.08 mL, 0.577 mmol). The reaction mixture was stirred at RT for 1 hour and solvent subsequently removed *in vacuo*. The crude material was purified by flash chromatography (silica gel, $CH_2Cl_2$-MeOH 95:5 to 80:20) to give **3a** as a slightly yellow oil (30 mg, 41%). $R_t$ 17.03 min (Gemini C18, 5 µm, 4.6 mm x 250 mm, 10% to 90% ACN, 0.1% TFA, over 18 min, 1 mL/min$^{-1}$, 300 nm). ESI-MS (m/z): calcd for $C_{63}H_{99}N_3O_{23}$ $[M+H]^+$ 1265.7, found 1266.6, $[M+NH_4]^+$ 1283.7, found 1283.6, $[M+Na]^+$ 1288.7, found 1288.6, $[M+K]^+$ 1304.6, found 1304.5.

**Compound CG1 PEG24:** To a solution of **1** (250 mg, 0.485 mmol) and maleimide-PEG$_{24}$-NHS ester (676 mg, 0.485 mmol) in N,N-dimethylacetamide (1 mL) was added $Et_3N$ (0.26 mL, 1.45 mmol). The reaction mixture was stirred at RT for 1 hour. The solvent was removed *in vacuo*. The crude material was purified by HPLC ($R_t$ 16.90 min, Gemini C18, 5 µm, 4.6 mm x 250 mm, 10% to 90% ACN, 0.1% TFA, over 18 min, 1 mL/min$^{-1}$, 300 nm) to give **3b** as a colorless oil (568 mg, 65%). ESI-MS (m/z): calcd for $C_{87}H_{147}N_3O_{35}$ $[M+H+Na]^{2+}$ 909.0, found 909.0, $[M+H+NH_4]^{2+}$ 906.5, found.906.5, $[M+H+K]^{2+}$ 917.0, found 917.5, $[M+NH_4+K]^{2+}$ 925.5, found 925.5, $[M+3H]^{3+}$ 599.0, found 598.9, $[M+2H+NH_4]^{3+}$ 604.7, found 604.7, $[M+H+2Na]^{3+}$: 613.7, found 613.7, $[M+H+Na+K]^{3+}$ 619.0, found 619.2, $[M+H+2K]^{3+}$ 624.3, found 624.6, $[M+NH_4+2K]^{3+}$ 630.0, found 629.9.

**4. Analytical data ($^{1}$H-NMR, $^{13}$C-NMR, MS and HPLC) for compounds CG1 their PEG derivatives and intermediates.**

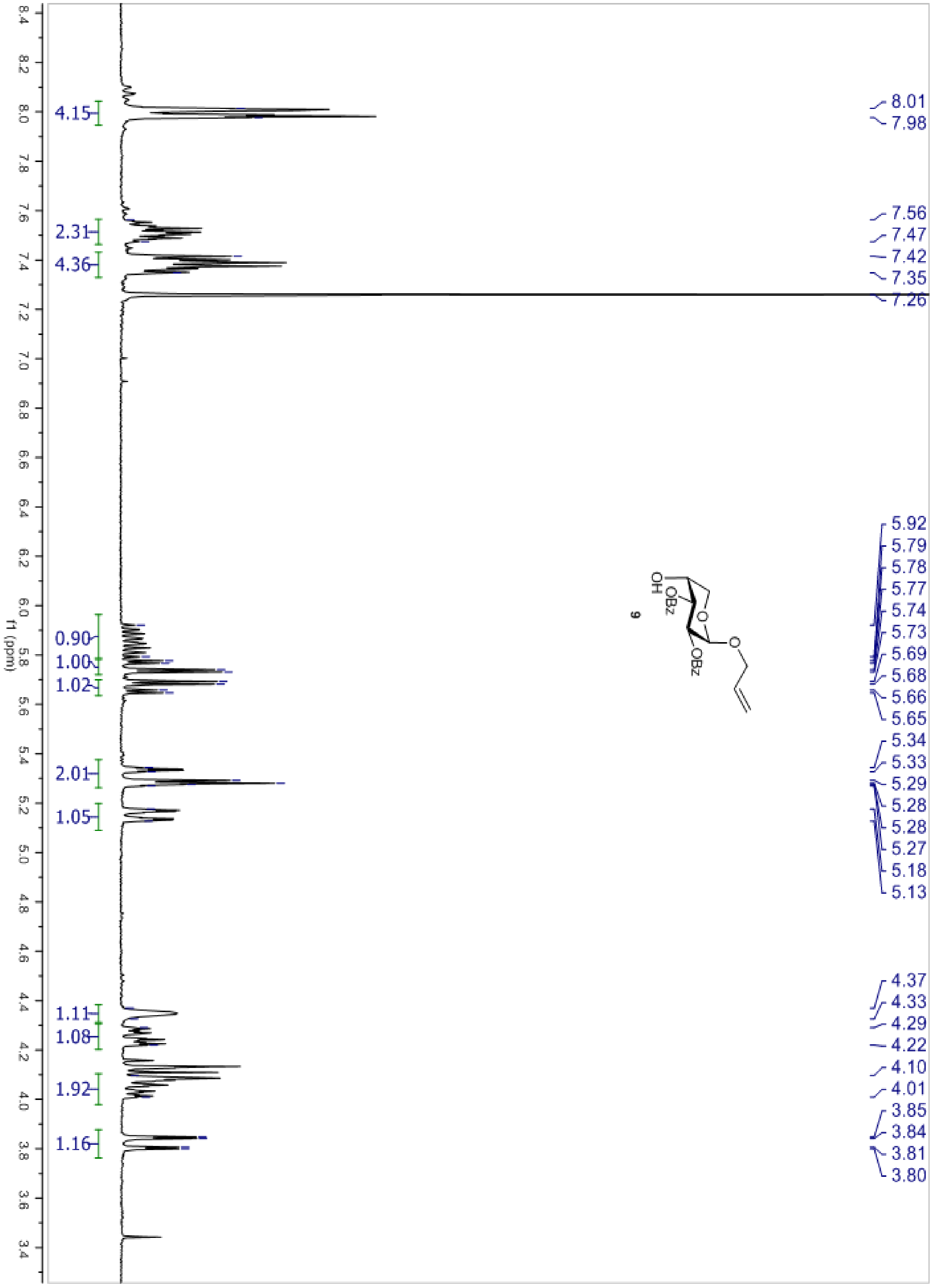

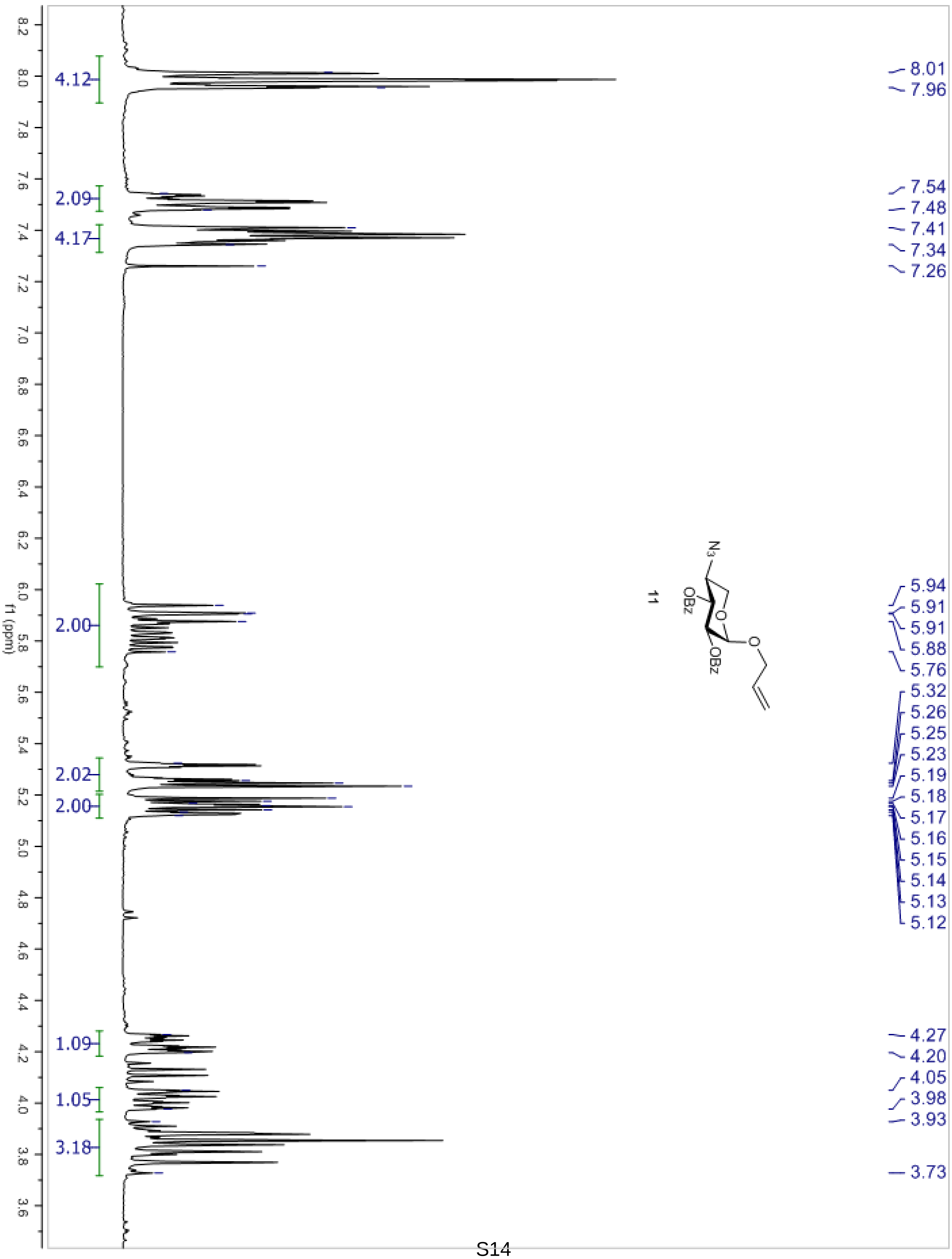
8.01
7.96
7.54
7.48
7.41
7.34
7.26
5.94
5.91
5.91
5.88
5.76
5.32
5.26
5.25
5.23
5.19
5.18
5.17
5.16
5.15
5.14
5.13
5.12
4.27
4.20
4.05
3.98
3.93
3.73
4.12
2.09
4.17
2.00
2.02
2.00
1.09
1.05
3.18
N3
OBz
O
OBz
O
11
f1 (ppm)
8.2
8.0
7.8
7.6
7.4
7.2
7.0
6.8
6.6
6.4
6.2
6.0
5.8
5.6
5.4
5.2
5.0
4.8
4.6
4.4
4.2
4.0
3.8
3.6

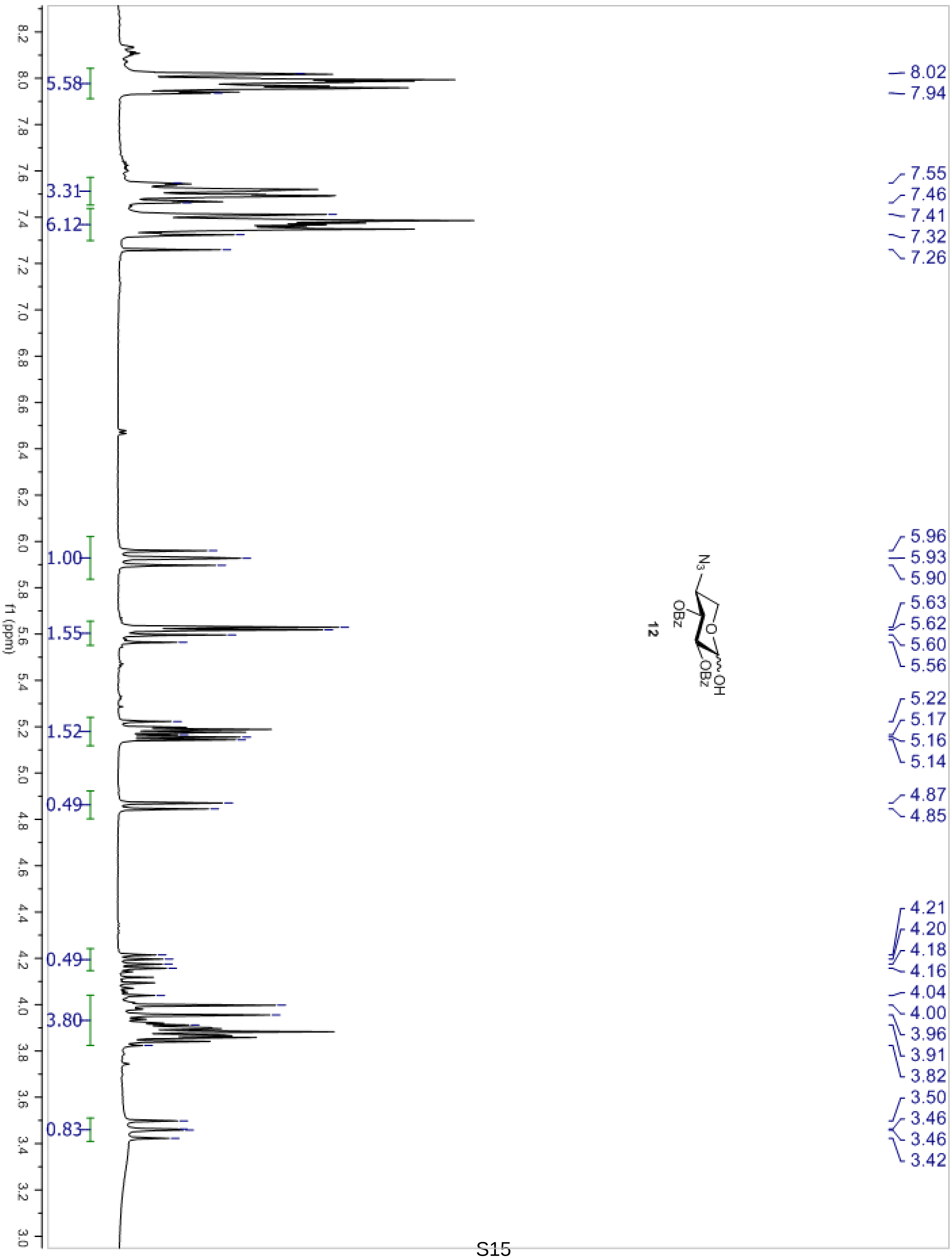
8.02
7.94
7.55
7.46
7.41
7.32
7.26
5.96
5.93
5.90
5.63
5.62
5.60
5.56
5.22
5.17
5.16
5.14
4.87
4.85
4.21
4.20
4.18
4.16
4.04
4.00
3.96
3.91
3.82
3.50
3.46
3.46
3.42
5.58
3.31
6.12
1.00
1.55
1.52
0.49
0.49
3.80
0.83
f1 (ppm)
N3
OBz
OBz
OH
O
12

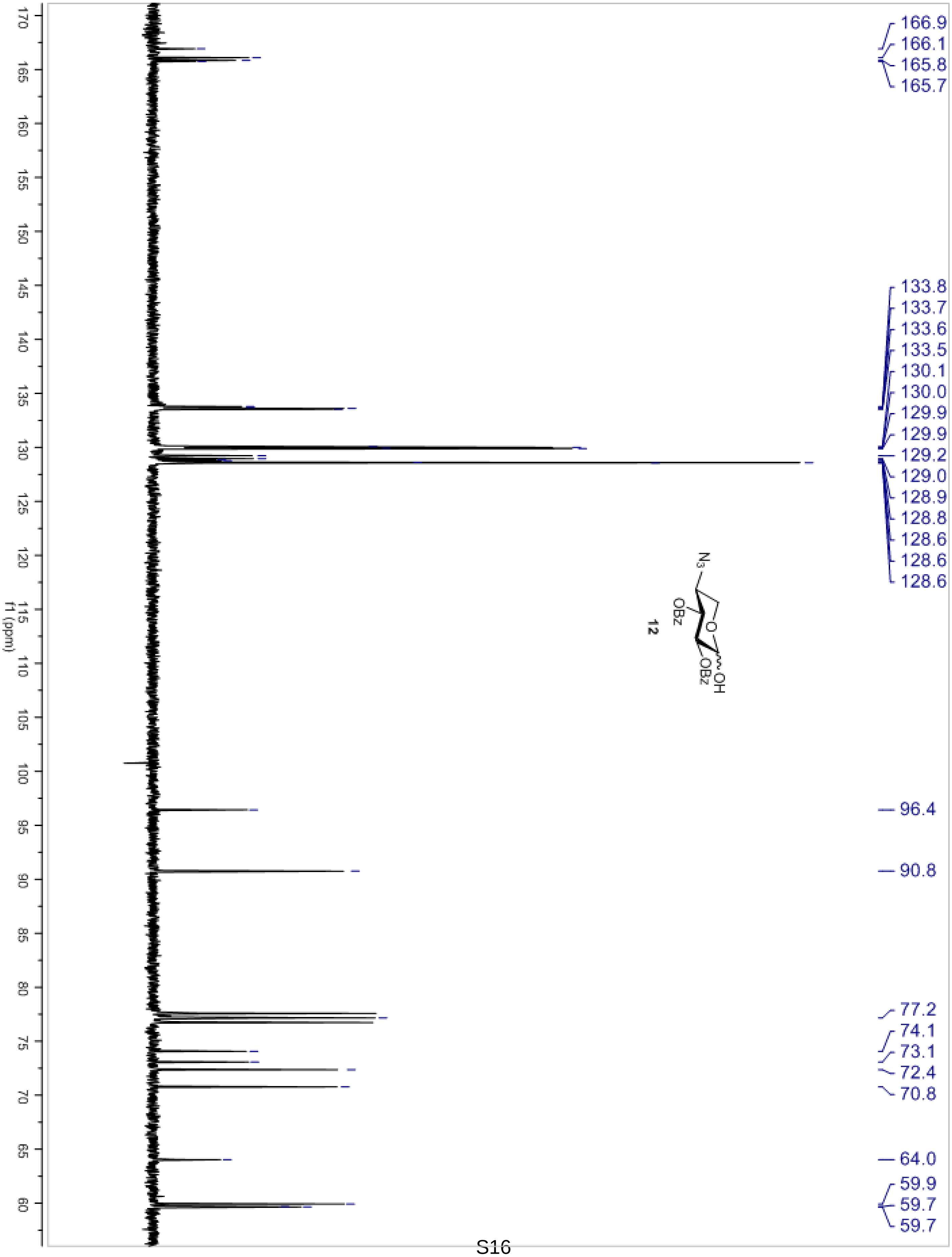
166.9
166.1
165.8
165.7
133.8
133.7
133.6
133.5
130.1
130.0
129.9
129.9
129.2
129.0
128.9
128.8
128.6
128.6
128.6
96.4
90.8
77.2
74.1
73.1
72.4
70.8
64.0
59.9
59.7
59.7
f1 (ppm)
N3
OBz
O
OBz
OH
12

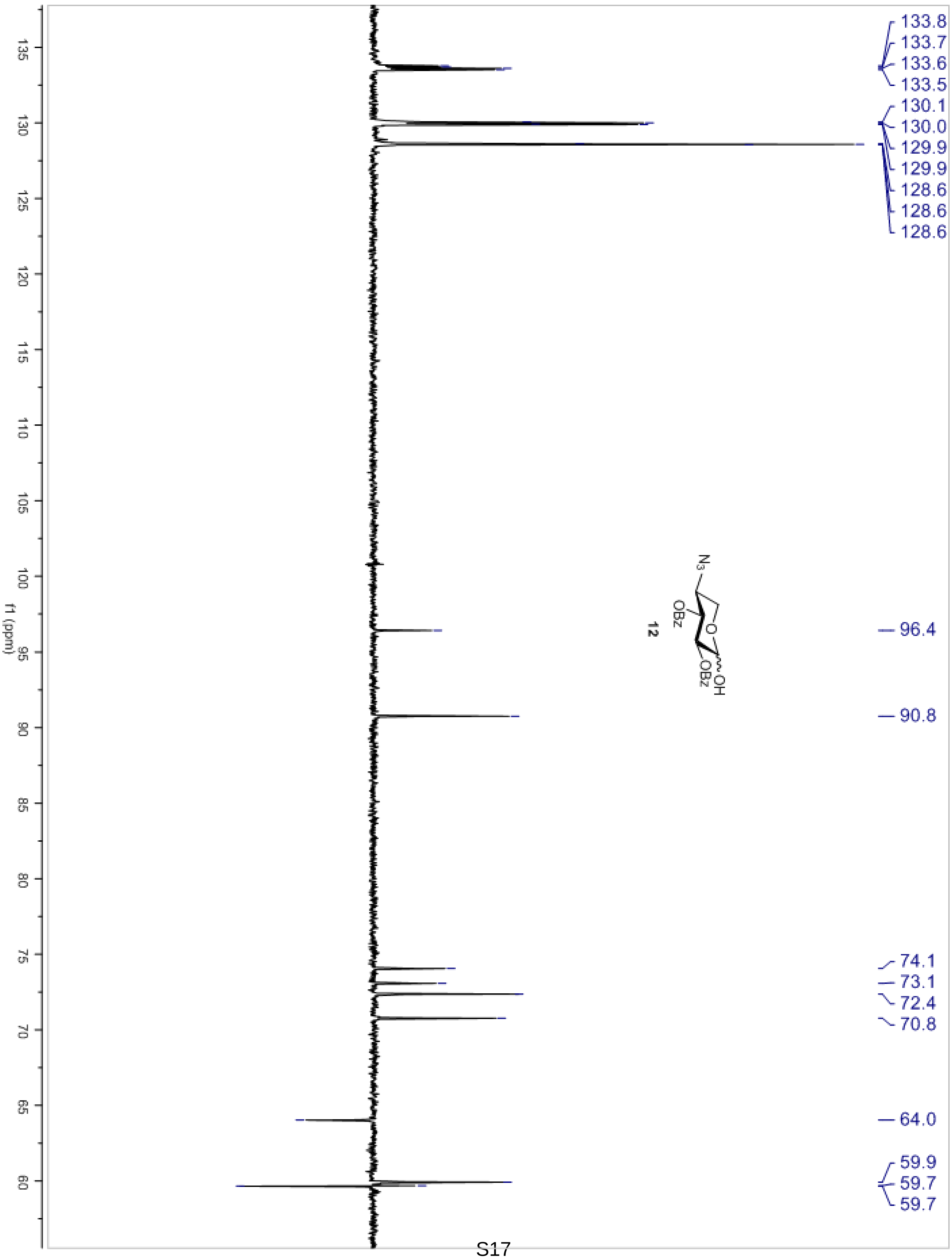
133.8
133.7
133.6
133.5
130.1
130.0
129.9
129.9
128.6
128.6
128.6
96.4
90.8
74.1
73.1
72.4
70.8
64.0
59.9
59.7
59.7
f1 (ppm)
N3
OBz
O
OH
OBz
12

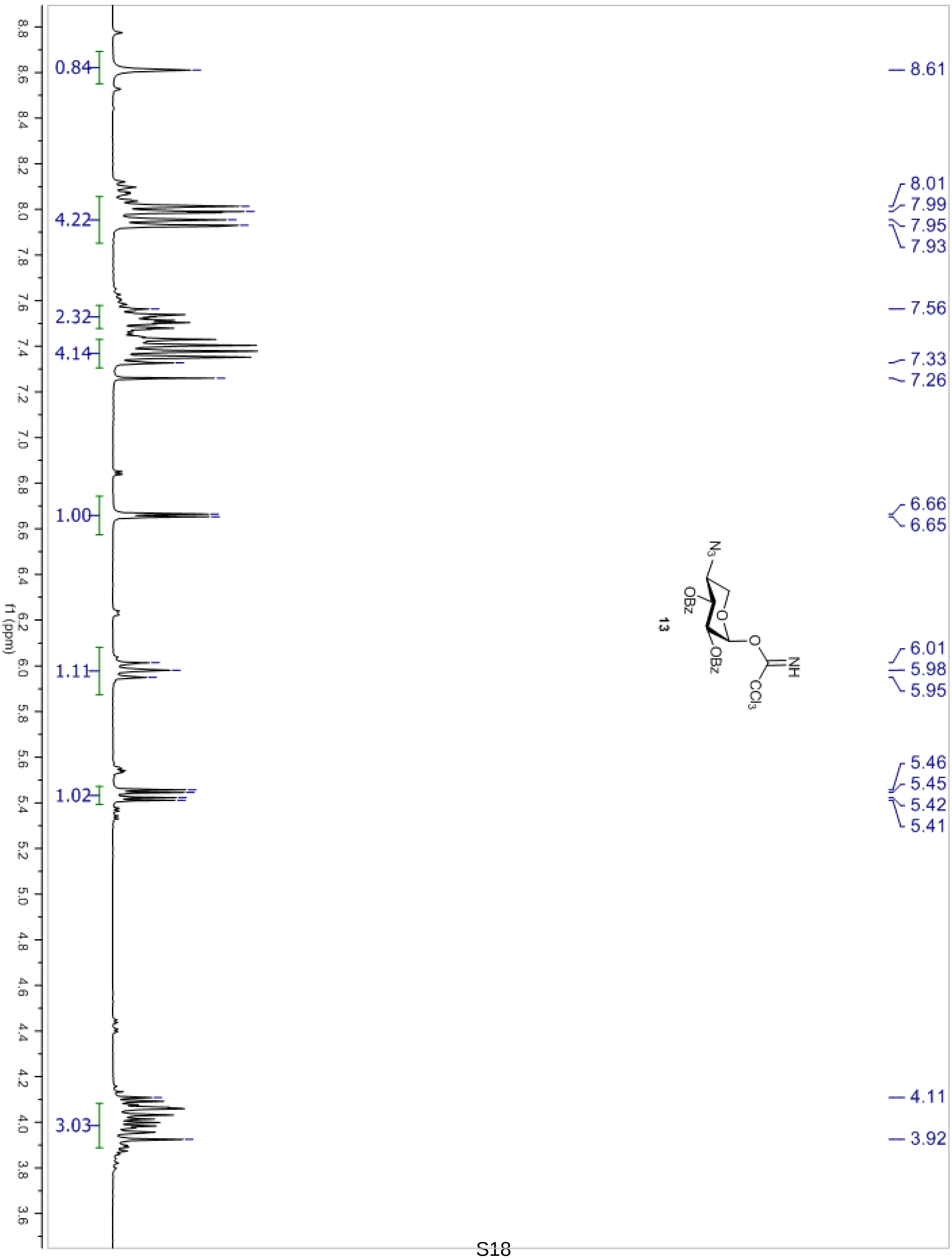

f1 (ppm)
0.84
4.22
2.32
4.14
1.00
1.11
1.02
3.03
8.61
8.01
7.99
7.95
7.93
7.56
7.33
7.26
6.66
6.65
6.01
5.98
5.95
5.46
5.45
5.42
5.41
4.11
3.92
N3
OBz
OBz
NH
CCl3
13

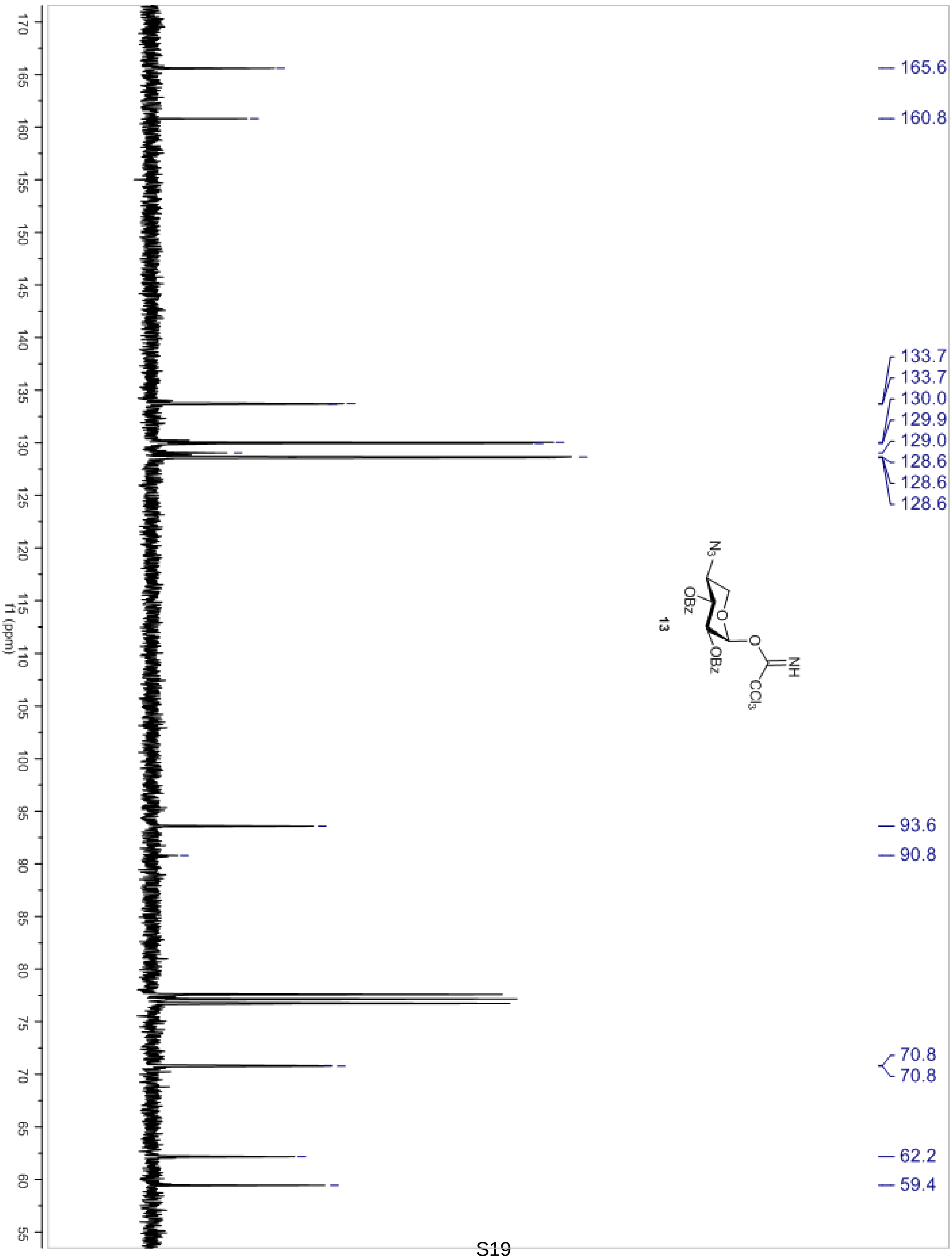
165.6
160.8
133.7
133.7
130.0
129.9
129.0
128.6
128.6
128.6
93.6
90.8
70.8
70.8
62.2
59.4
f1 (ppm)
N3
OBz
OBz
O
NH
CCl3
13

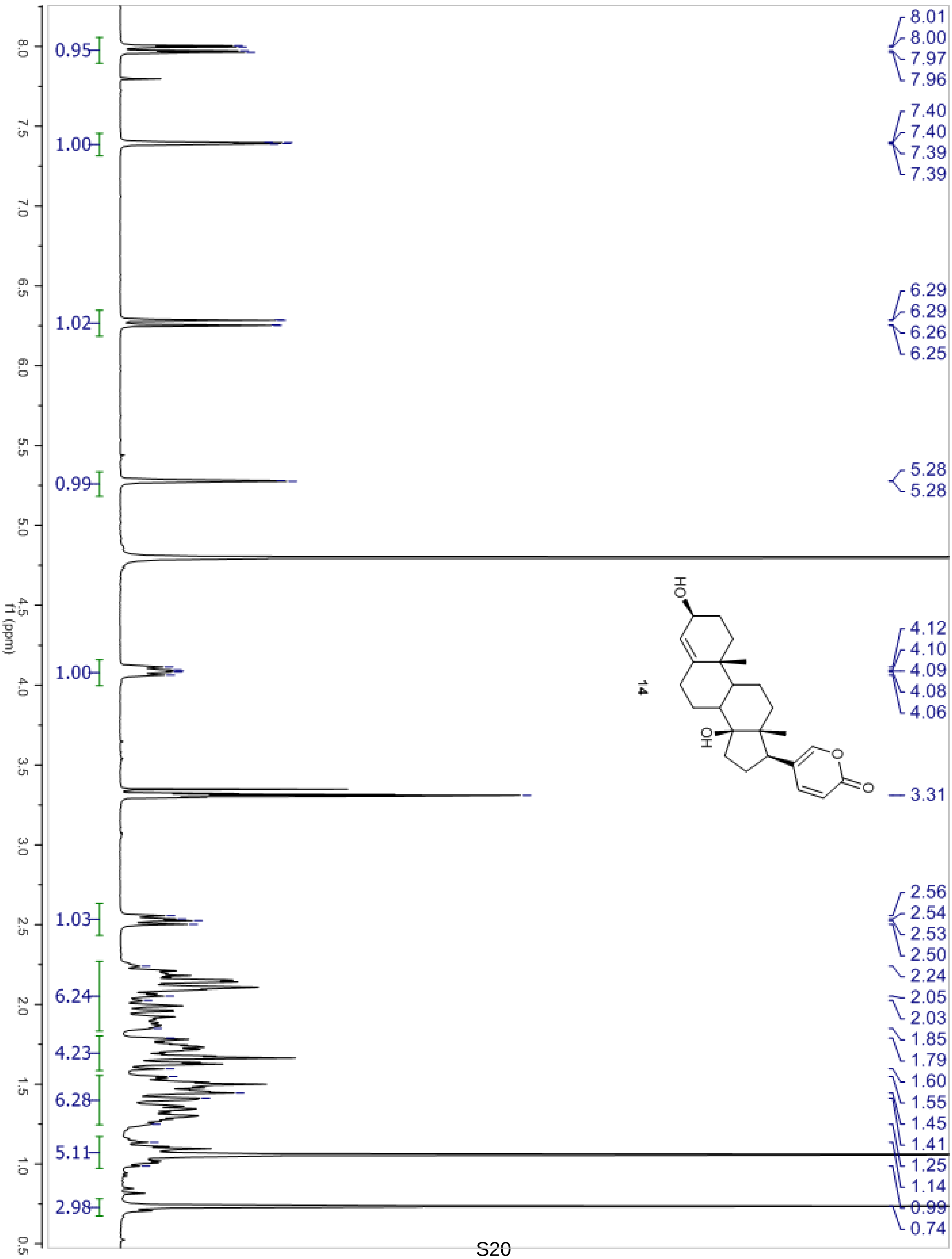
8.01
8.00
7.97
7.96
7.40
7.40
7.39
7.39
6.29
6.29
6.26
6.25
5.28
5.28
4.12
4.10
4.09
4.08
4.06
3.31
2.56
2.54
2.53
2.50
2.24
2.05
2.03
1.85
1.79
1.60
1.55
1.45
1.41
1.25
1.14
0.99
0.74
0.95
1.00
1.02
0.99
1.00
1.03
6.24
4.23
6.28
5.11
2.98
HO
OH
O
14
f1 (ppm)

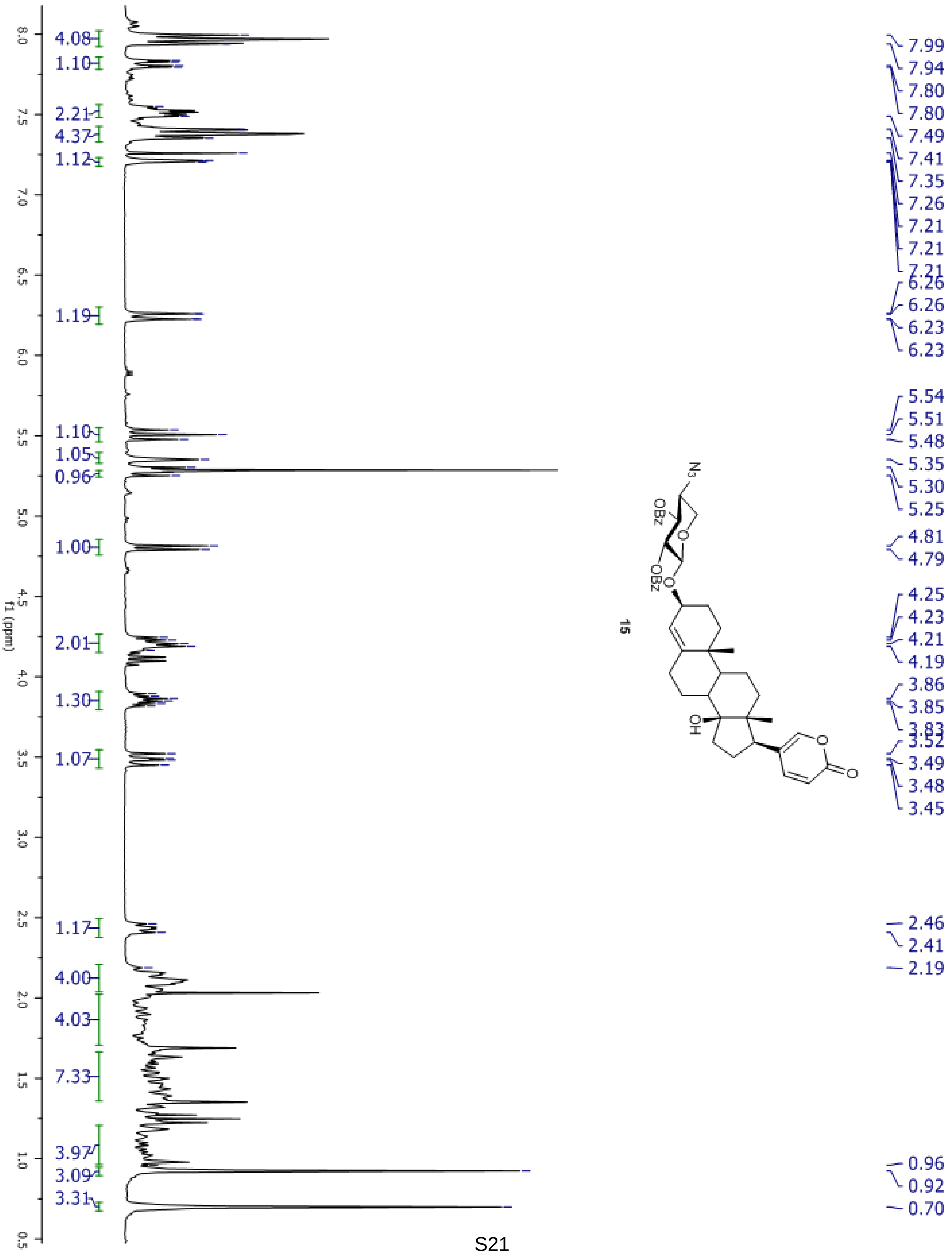
4.08
1.10
2.21
4.37
1.12
1.19
1.10
1.05
0.96
1.00
2.01
1.30
1.07
1.17
4.00
4.03
7.33
3.97
3.09
3.31
f1 (ppm)
7.99
7.94
7.80
7.80
7.49
7.41
7.35
7.26
7.21
7.21
7.21
6.26
6.26
6.23
6.23
5.54
5.51
5.48
5.35
5.30
5.25
4.81
4.79
4.25
4.23
4.21
4.19
3.86
3.85
3.83
3.52
3.49
3.48
3.45
2.46
2.41
2.19
0.96
0.92
0.70
N3
OBz
OBz
O
OH
15

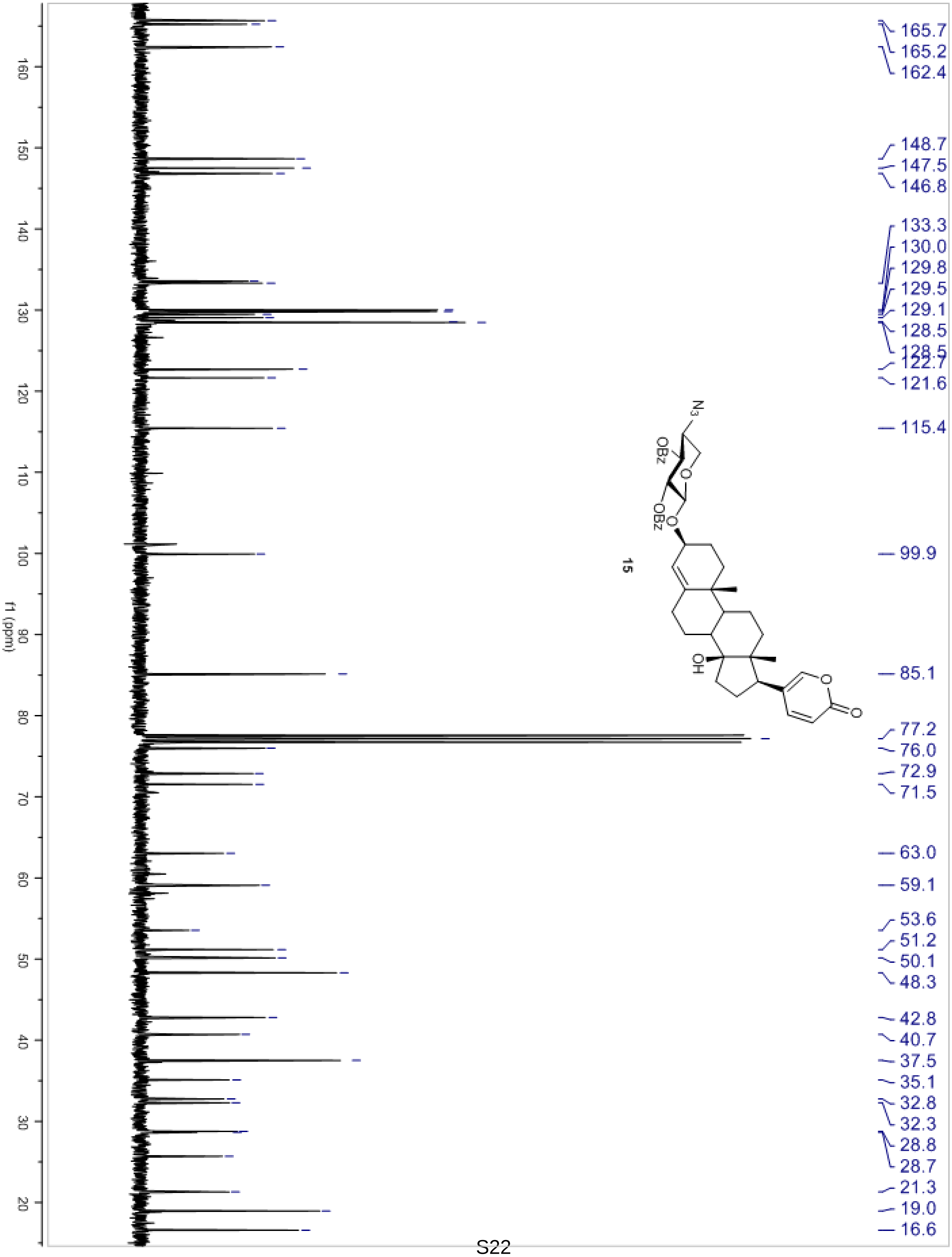
165.7
165.2
162.4
148.7
147.5
146.8
133.3
130.0
129.8
129.5
129.1
128.5
128.5
122.7
121.6
115.4
99.9
85.1
77.2
76.0
72.9
71.5
63.0
59.1
53.6
51.2
50.1
48.3
42.8
40.7
37.5
35.1
32.8
32.3
28.8
28.7
21.3
19.0
16.6
f1 (ppm)
15

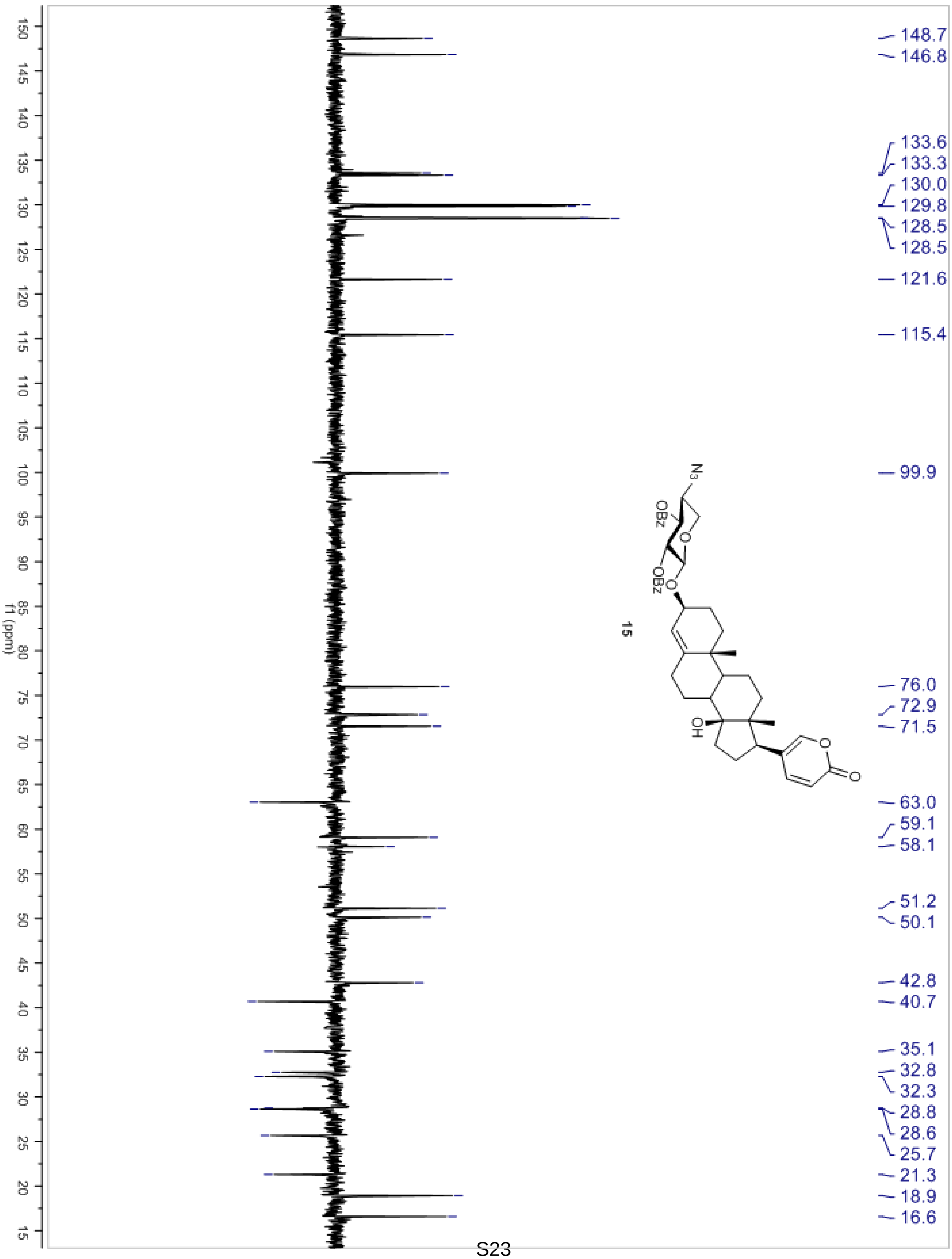
148.7
146.8
133.6
133.3
130.0
129.8
128.5
128.5
121.6
115.4
99.9
76.0
72.9
71.5
63.0
59.1
58.1
51.2
50.1
42.8
40.7
35.1
32.8
32.3
28.8
28.6
25.7
21.3
18.9
16.6
15
f1 (ppm)

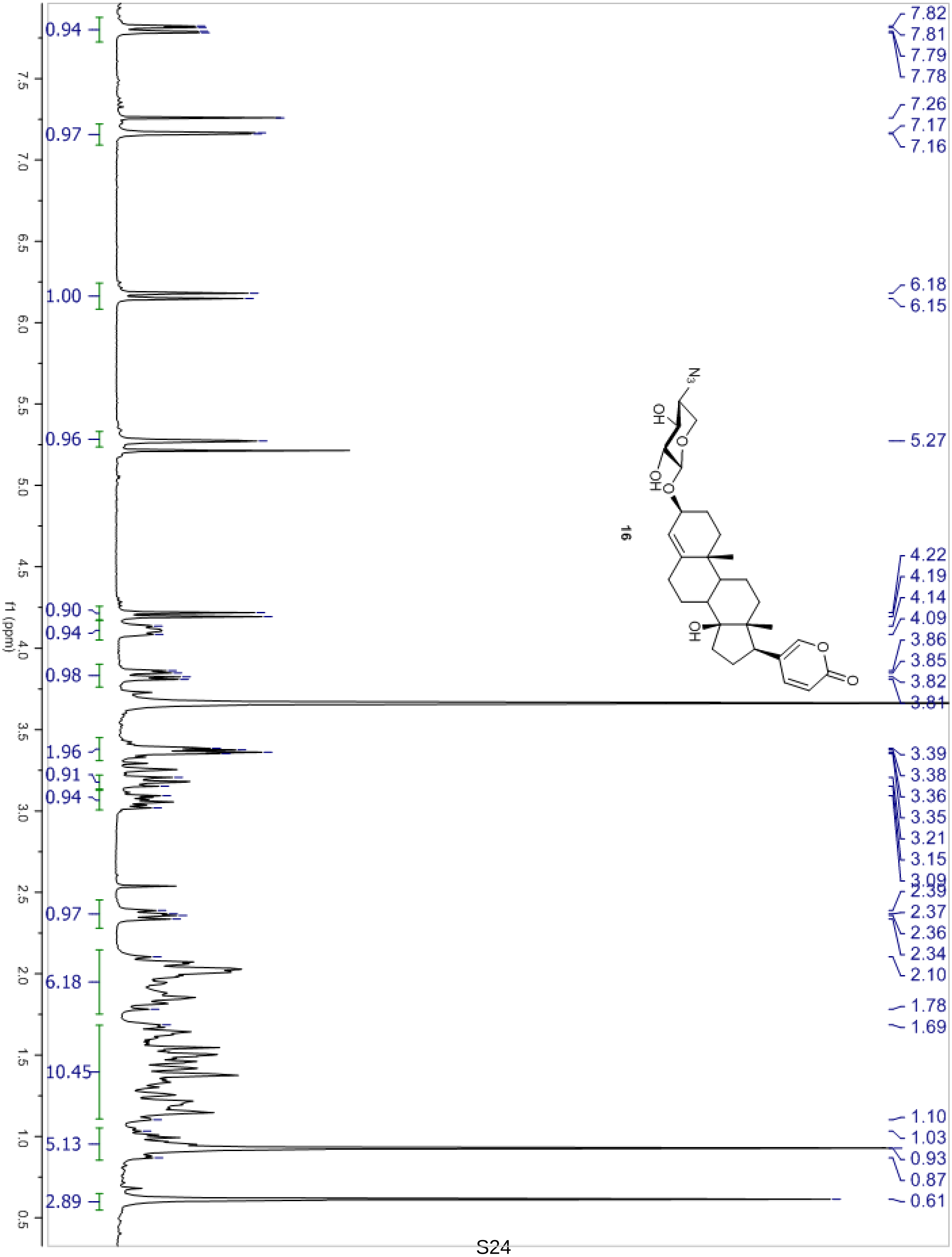
7.82
7.81
7.79
7.78
7.26
7.17
7.16
6.18
6.15
5.27
4.22
4.19
4.14
4.09
3.86
3.85
3.82
3.81
3.39
3.38
3.36
3.35
3.21
3.15
3.09
2.39
2.37
2.36
2.34
2.10
1.78
1.69
1.10
1.03
0.93
0.87
0.61
0.94
0.97
1.00
0.96
0.90
0.94
0.98
1.96
0.91
0.94
0.97
6.18
10.45
5.13
2.89
f1 (ppm)
$N_3$
OH
OH
OH
O
O
O
O
16

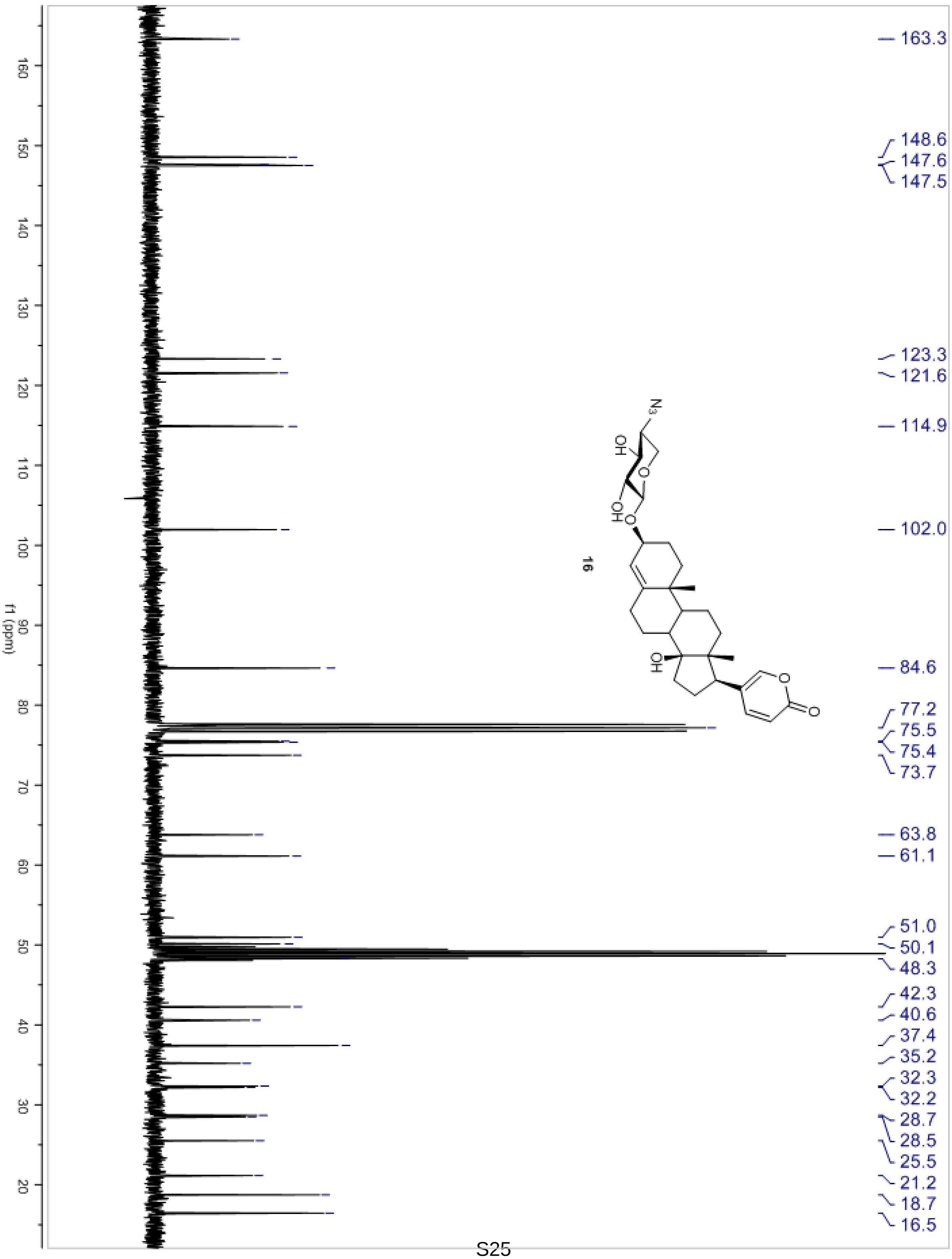
163.3
148.6
147.6
147.5
123.3
121.6
114.9
102.0
84.6
77.2
75.5
75.4
73.7
63.8
61.1
51.0
50.1
48.3
42.3
40.6
37.4
35.2
32.3
32.2
28.7
28.5
25.5
21.2
18.7
16.5
f1 (ppm)
16

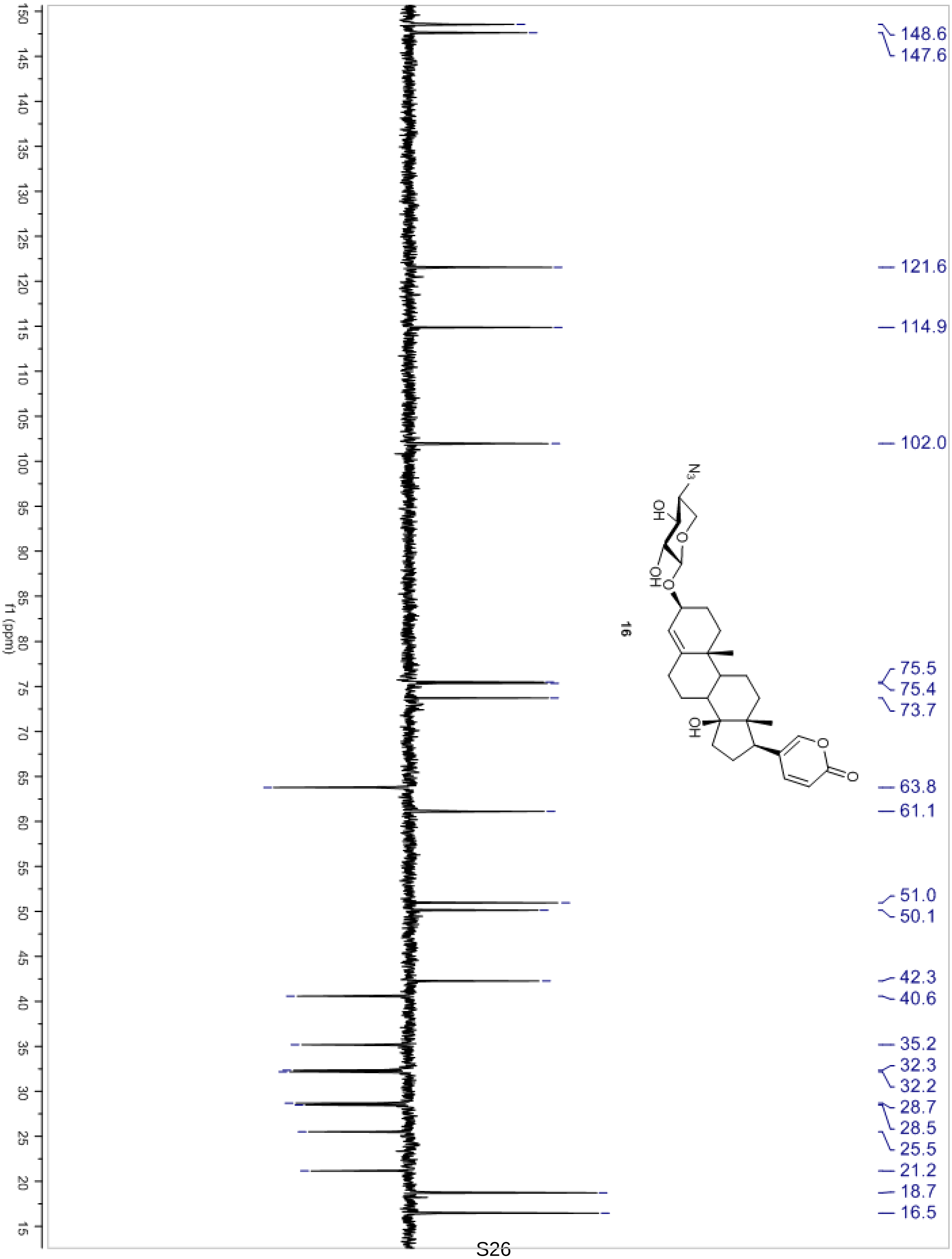
148.6
147.6
121.6
114.9
102.0
75.5
75.4
73.7
63.8
61.1
51.0
50.1
42.3
40.6
35.2
32.3
32.2
28.7
28.5
25.5
21.2
18.7
16.5
f1 (ppm)
16
N3
OH
OH
OH

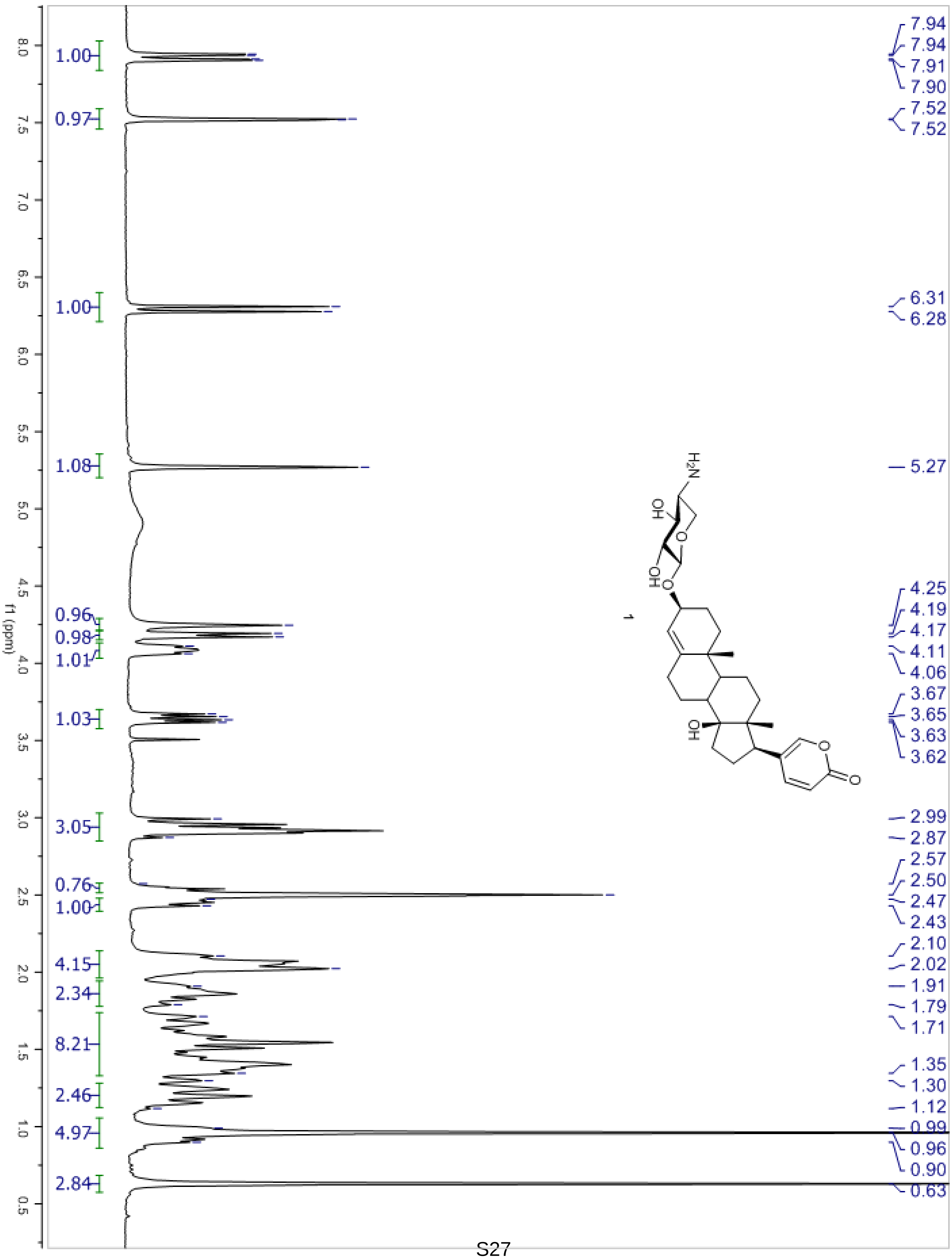
7.94
7.94
7.91
7.90
7.52
7.52
6.31
6.28
5.27
4.25
4.19
4.17
4.11
4.06
3.67
3.65
3.63
3.62
2.99
2.87
2.57
2.50
2.47
2.43
2.10
2.02
1.91
1.79
1.71
1.35
1.30
1.12
0.99
0.96
0.90
0.63
1.00
0.97
1.00
1.08
0.96
0.98
1.01
1.03
3.05
0.76
1.00
4.15
2.34
8.21
2.46
4.97
2.84
f1 (ppm)
1

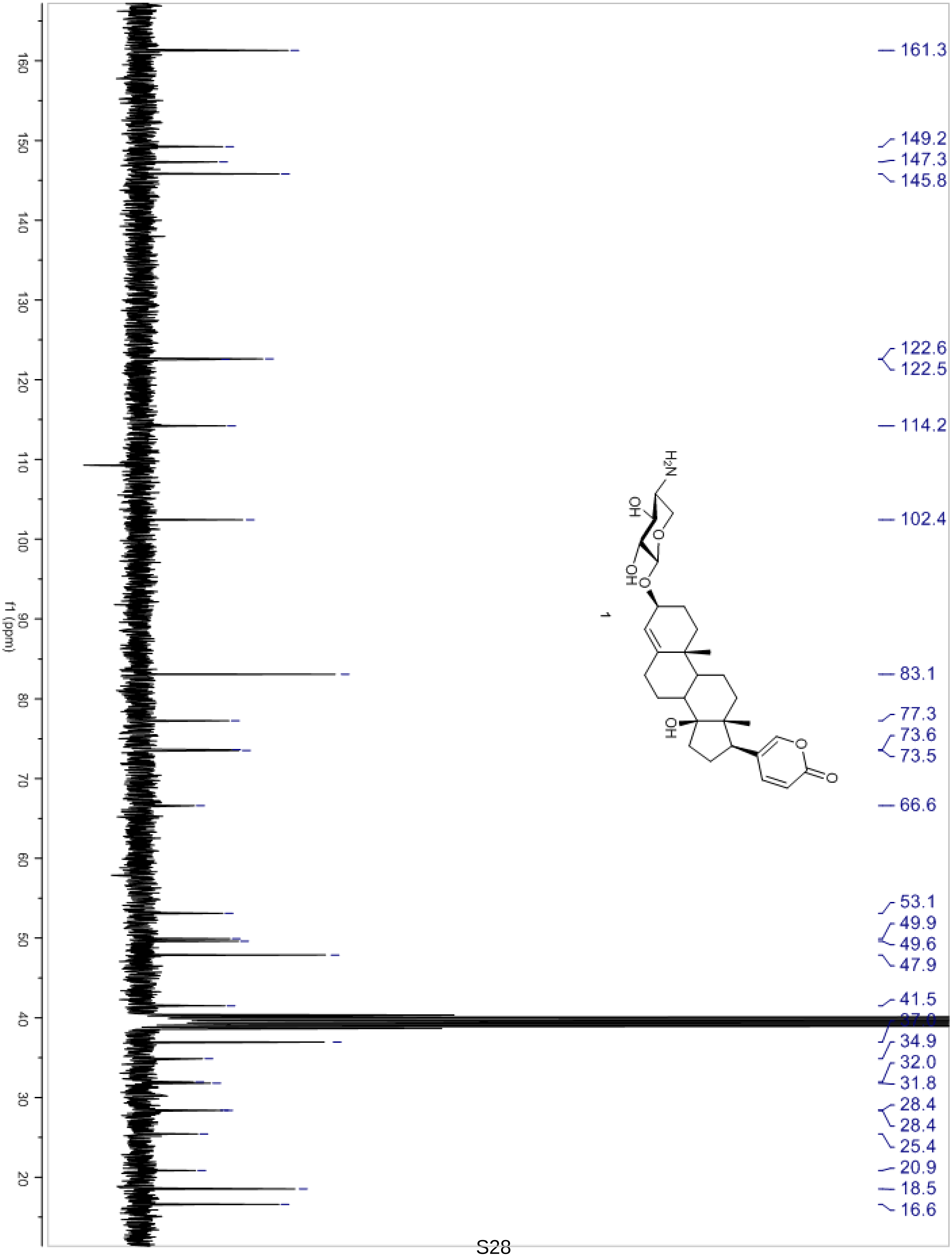
161.3
149.2
147.3
145.8
122.6
122.5
114.2
102.4
83.1
77.3
73.6
73.5
66.6
53.1
49.9
49.6
47.9
41.5
34.9
32.0
31.8
28.4
28.4
25.4
20.9
18.5
16.6
f1 (ppm)
1

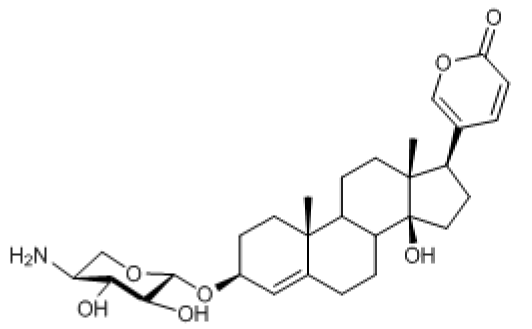
H2N
O
O
OH
OH
OH
O
O

1

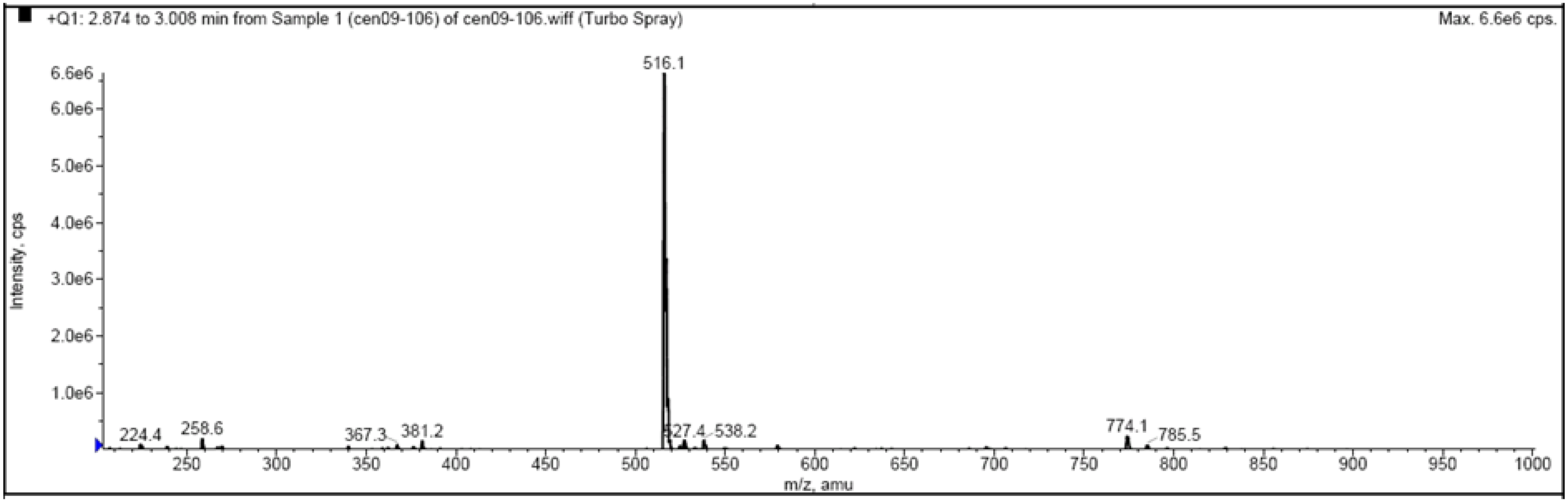
+Q1: 2.874 to 3.008 min from Sample 1 (cen09-106) of cen09-106.wiff (Turbo Spray)
Max. 6.6e6 cps.
Intensity, cps
6.6e6
6.0e6
5.0e6
4.0e6
3.0e6
2.0e6
1.0e6
516.1
224.4
258.6
367.3
381.2
527.4
538.2
774.1
785.5
250
300
350
400
450
500
550
600
650
700
750
800
850
900
950
1000
m/z, amu

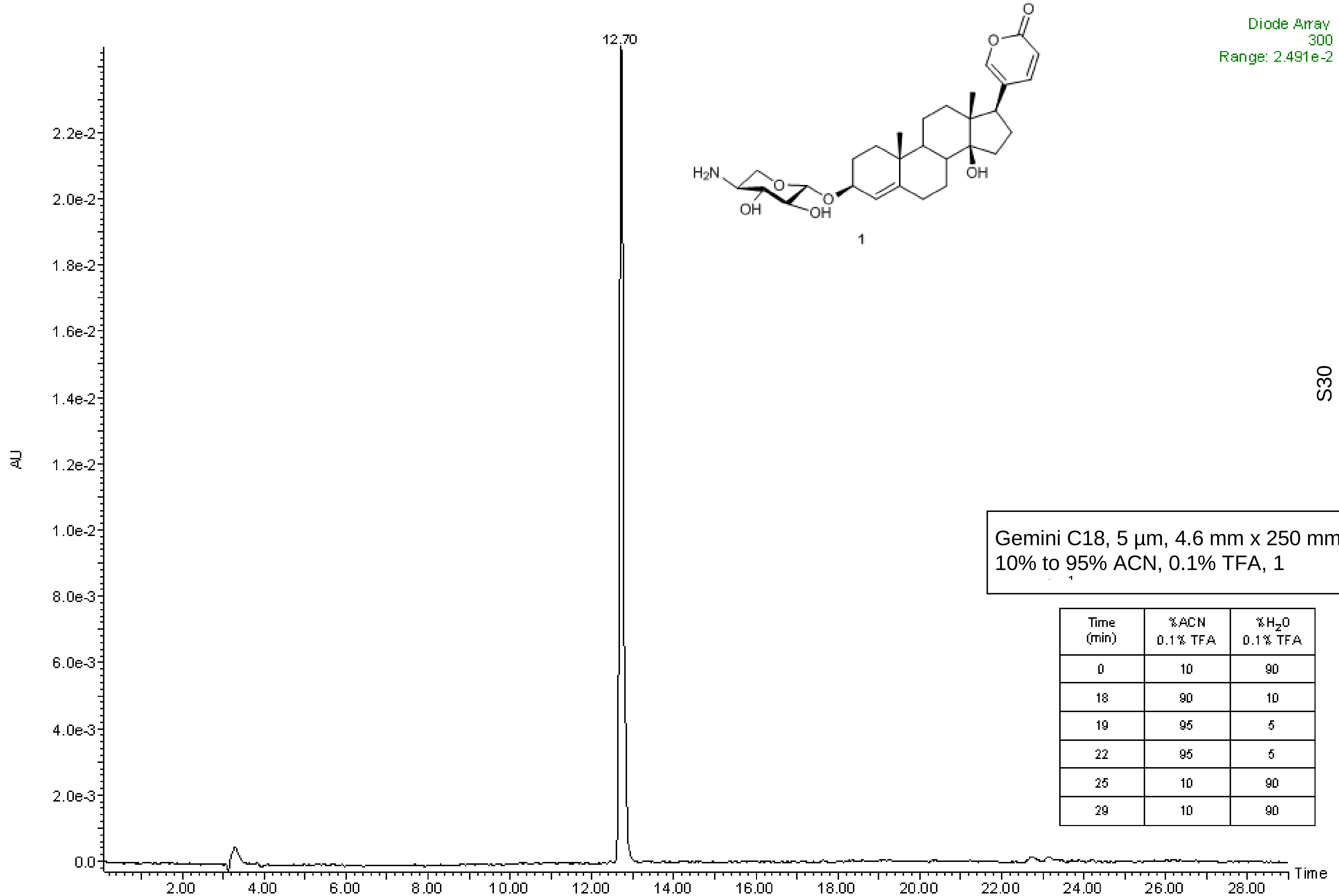


1

Gemini C18, 5 µm, 4.6 mm x 250 mm, 10% to 95% ACN, 0.1% TFA, 1

| Time (min) | %ACN 0.1% TFA | $\%H_2O$ 0.1% TFA |
|---|---|---|
| 0 | 10 | 90 |
| 18 | 90 | 10 |
| 19 | 95 | 5 |
| 22 | 95 | 5 |
| 25 | 10 | 90 |
| 29 | 10 | 90 |

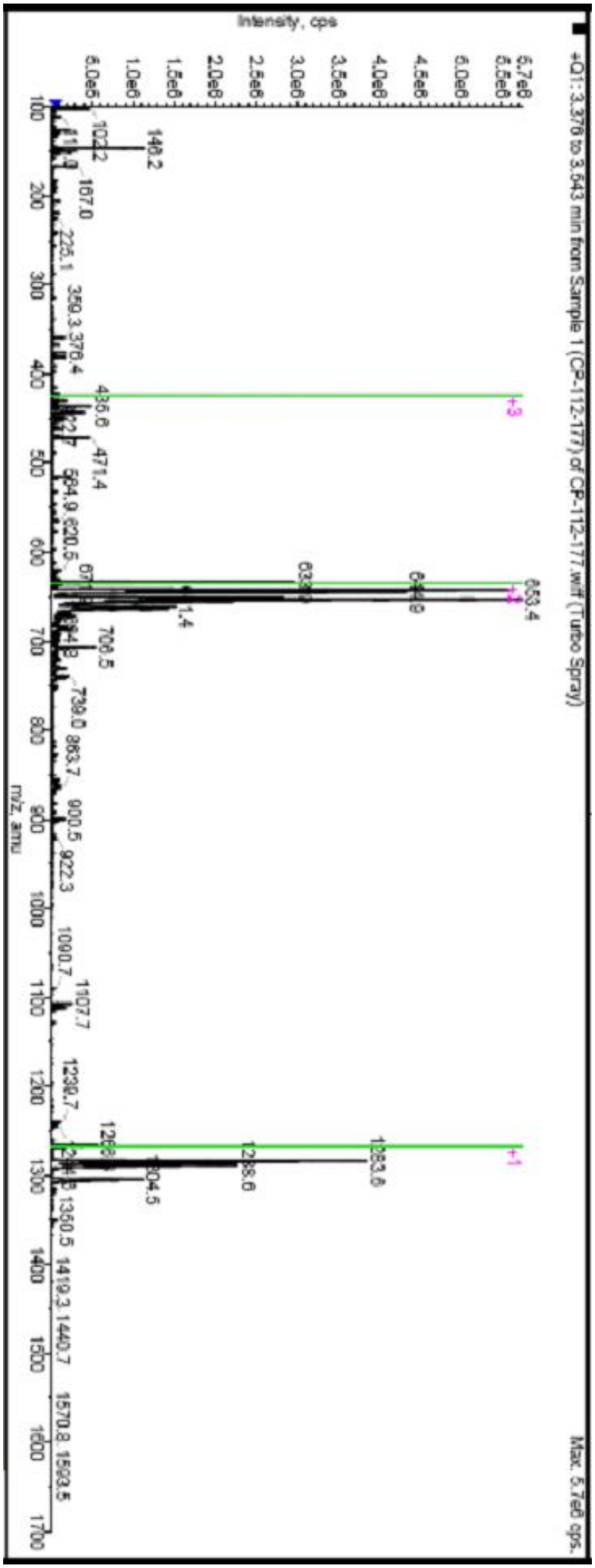
+Q1: 3.376 to 3.543 min from Sample 1 (CP-112-177) of CP-112-177.wiff (Turbo Spray)
Max. 5.7e6 cps.
Intensity, cps
m/z, amu
146.2
653.4
644.8
1283.6
1288.6
1304.5
706.5
+3
+2
+1

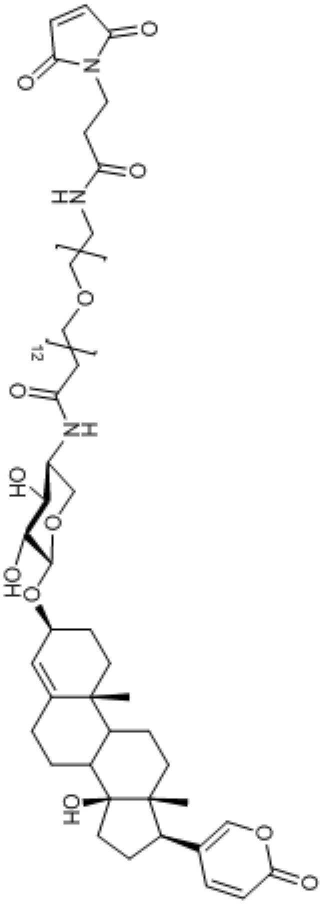
12

3a

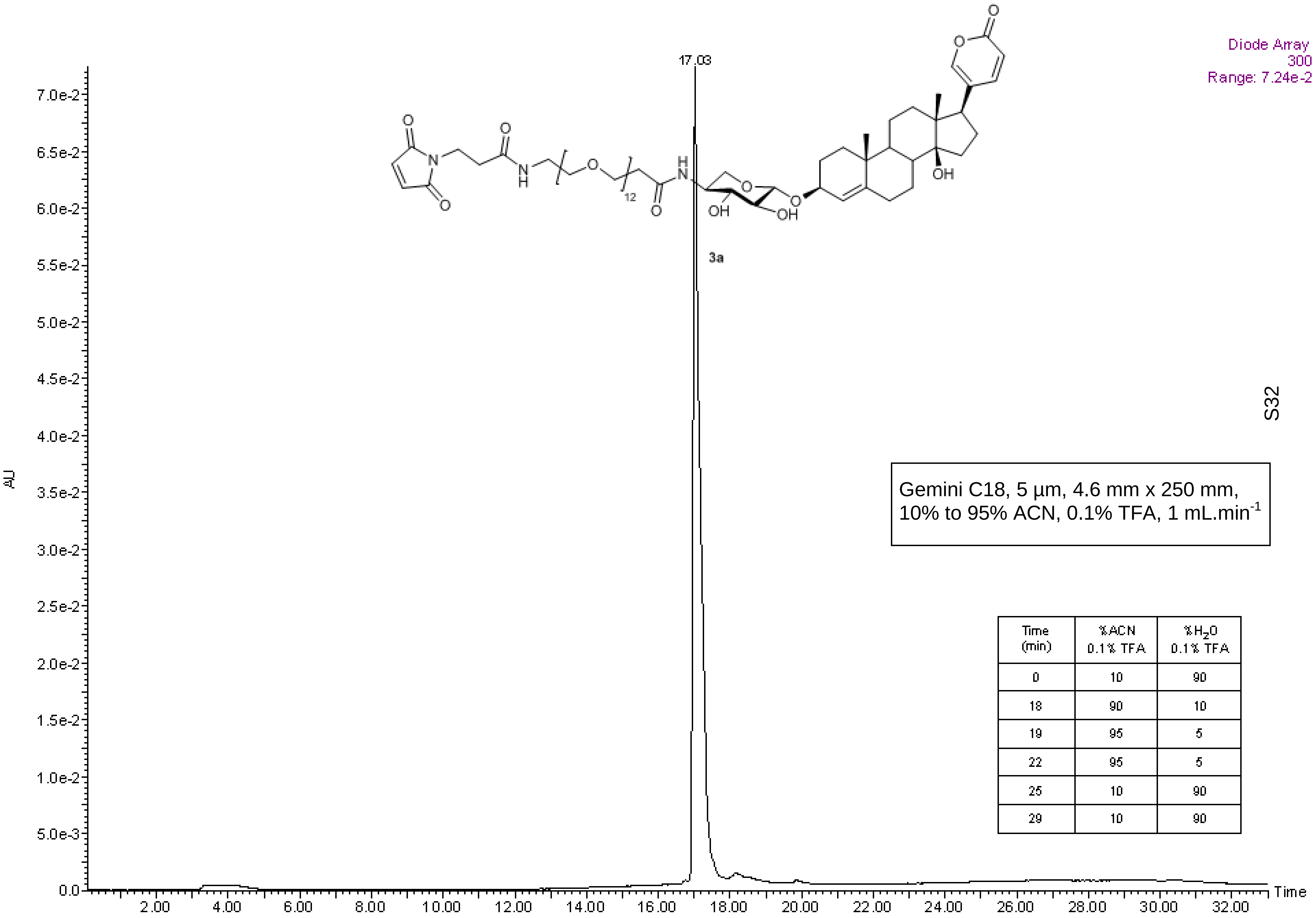


Gemini C18, 5 µm, 4.6 mm x 250 mm, 10% to 95% ACN, 0.1% TFA, 1 mL.min$^{-1}$

| Time (min) | %ACN 0.1% TFA | %$H_2O$ 0.1% TFA |
|---|---|---|
| 0 | 10 | 90 |
| 18 | 90 | 10 |
| 19 | 95 | 5 |
| 22 | 95 | 5 |
| 25 | 10 | 90 |
| 29 | 10 | 90 |

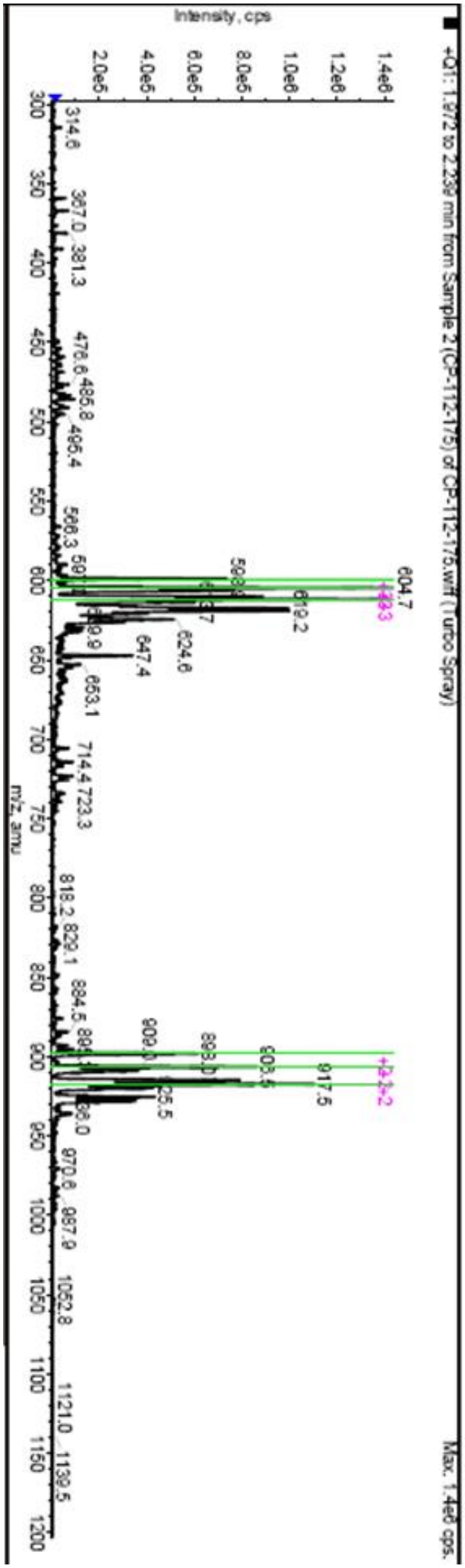

+Q1: 1.972 to 2.239 min from Sample 2 (CP-112-175) of CP-112-175.wiff (Turbo Spray)
Max. 1.4e6 cps.
Intensity, cps
m/z, amu
604.7
619.2
647.4
624.6
917.5


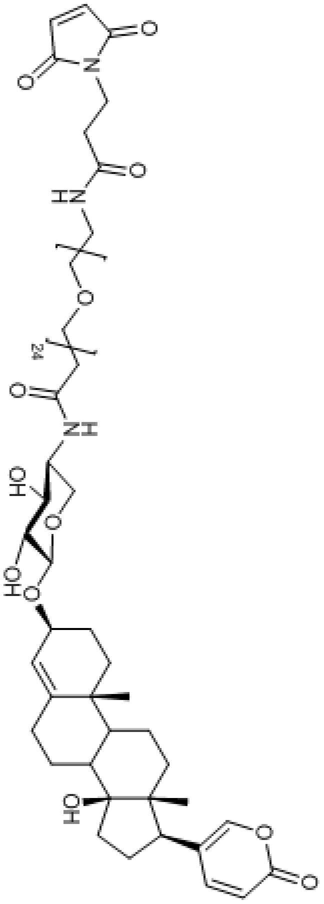

3b

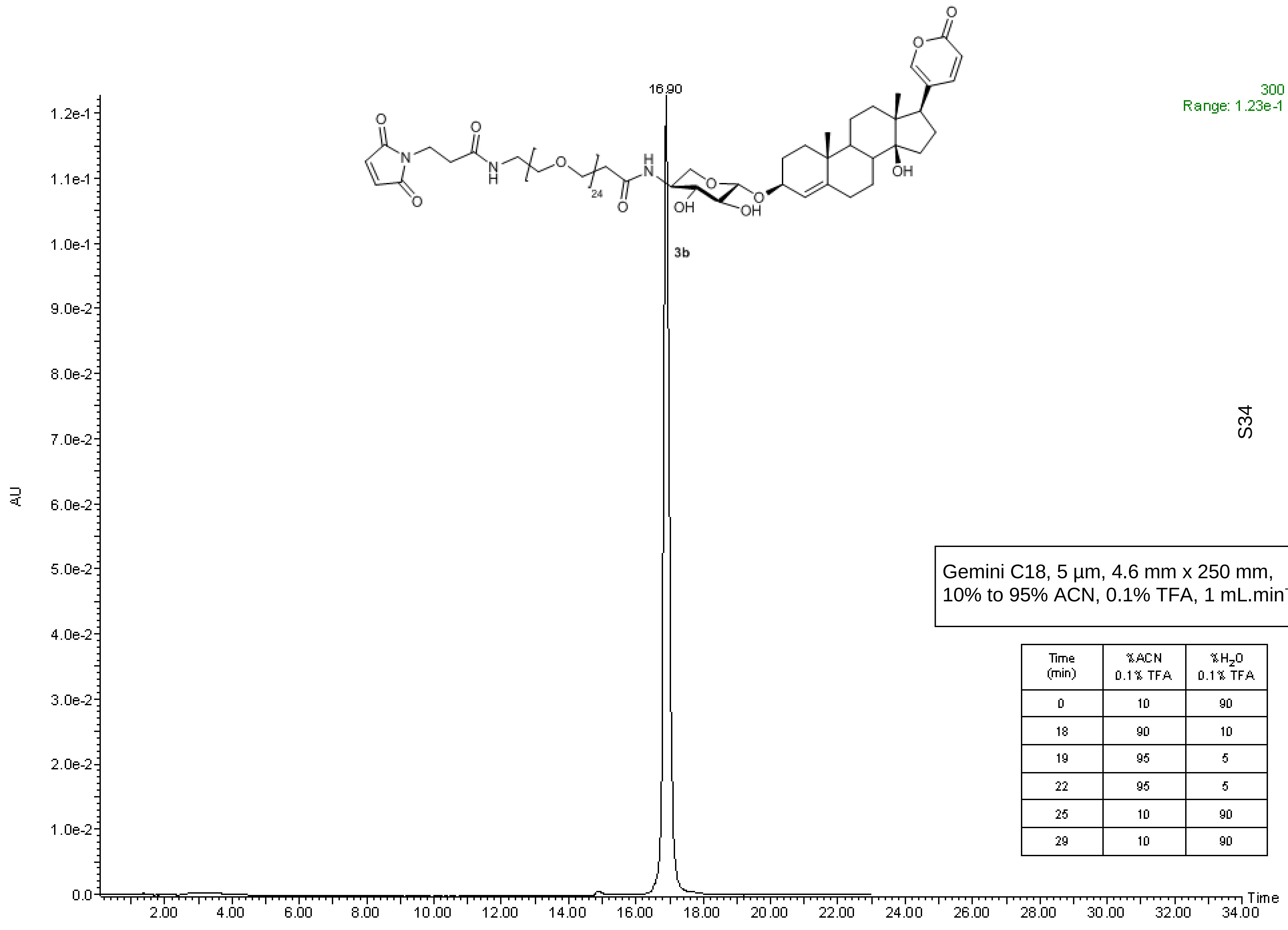


Gemini C18, 5 µm, 4.6 mm x 250 mm,
10% to 95% ACN, 0.1% TFA, 1 $mL.min^{-1}$

| Time (min) | %ACN 0.1% TFA | %$H_2O$ 0.1% TFA |
|---|---|---|
| 0 | 10 | 90 |
| 18 | 90 | 10 |
| 19 | 95 | 5 |
| 22 | 95 | 5 |
| 25 | 10 | 90 |
| 29 | 10 | 90 |
